# A Small-Volume, Low-Cost, and Versatile Continuous Culture Device

**DOI:** 10.1371/journal.pone.0133384

**Published:** 2015-07-21

**Authors:** Dominick Matteau, Vincent Baby, Stéphane Pelletier, Sébastien Rodrigue

**Affiliations:** 1 Département de biologie, Université de Sherbrooke, Sherbrooke, Québec, Canada; 2 Département de physique, Université de Sherbrooke, Sherbrooke, Québec, Canada; Glasgow University, UNITED KINGDOM

## Abstract

**Background:**

Continuous culture devices can be used for various purposes such as establishing reproducible growth conditions or maintaining cell populations under a constant environment for long periods. However, commercially available instruments are expensive, were not designed to handle small volumes in the milliliter range, and can lack the flexibility required for the diverse experimental needs found in several laboratories.

**Methodology/Principal Findings:**

We developed a versatile continuous culture system and provide detailed instructions as well as a graphical user interface software for potential users to assemble and operate their own instrument. Three culture chambers can be controlled simultaneously with the proposed configuration, and all components are readily available from various sources. We demonstrate that our continuous culture device can be used under different modes, and can easily be programmed to behave either as a turbidostat or chemostat. Addition of fresh medium to the culture vessel can be controlled by a real-time feedback loop or simply calibrated to deliver a defined volume. Furthermore, the selected light-emitting diode and photodetector enable the use of phenol red as a pH indicator, which can be used to indirectly monitor the bulk metabolic activity of a cell population rather than the turbidity.

**Conclusions/Significance:**

This affordable and customizable system will constitute a useful tool in many areas of biology such as microbial ecology as well as systems and synthetic biology.

## Introduction

Since their introduction in 1950 [1,2], continuous culture devices have been useful to study the biology of various species and were adopted in many industrial contexts such as brewing and waste treatment [3]. Their principle of operation essentially consists in using fresh medium to dilute cells and waste products as they accumulate in the growth chamber. The two main continuous culture devices are chemostats and turbidostats, but variants and other types of systems have been described [4–13]. Chemostats, for “chemical environment is static”, are used to maintain cells in a physiological steady state through dilution of a liquid culture at a specified rate with a medium limited for specific nutrients, thus restraining cell growth to a fixed rate. In contrast, turbidostats maintain cell density by adding fresh medium in response to an increased optical density of a culture. Turbidity is constantly monitored and the culture is refreshed through a feedback control loop to constrain cell concentration within a narrow range. Turbidostats are more appropriate than chemostats for experiments that require cells to proliferate at their maximum growth rate, since no limitation of nutrients is applied.

Continuous culture conditions result in a stable and controllable set of physico-chemical conditions in which growth rate, pH, biomass, as well as the concentrations of dissolved oxygen, proteins, and metabolites reach a dynamic equilibrium and remain approximately constant over an extended period of time [3,9,14,15]. These highly-reproducible parameters contribute to the establishment of a physiological steady state in cell populations, which decreases variability in quantitative experiments such as RNA sequencing and mass spectrometry [14–17]. Systems biology that aims at analyzing and integrating large amounts of data to understand complex interactions between components of biological systems would thus particularly benefit from controlled and highly-reproducible growth parameters provided by continuous culture devices [14,18].

Despite of its multiple advantages, the use of continuous culture, more specifically in the context of systems biology, is not common. This is not surprising since commercially available continuous culture instruments are expensive, not designed to handle small volumes, rarely customizable, and can lack the flexibility required to accommodate a large diversity of experimental conditions. As a consequence, most studies are thus conducted using batch cultures, a simple, easy to implement, low cost, and highly scalable cultivation procedure that however results in a continuously fluctuating chemical environment [17,19]. Some laboratories have developed continuous culture instruments for their specific needs. However, the design and utilization of these custom instruments is often limited to particular experimental conditions and/or specific types of organisms [3]. In addition, relatively few publications have described these devices, and even fewer provide detailed technical information along with a software to operate a device [7,20–22]. To address this issue, we have conceived and built the Versatile Continuous Culture Device (VCCD), a flexible, open-source, small-volume, and low-cost continuous cultivation system that can easily be programmed to act either as a turbidostat or chemostat. Here, we describe the features and capabilities of the VCCD. We also provide the required information for the construction, operation and modification of this system along with the source code of the associated software. We anticipate that the VCCD will be particularly useful in the fields of microbial ecology, functional genomics, synthetic biology, and any other discipline in which constant and controllable growth conditions are advantageous or crucial.

## Materials and Methods

### Strains and growth conditions

*E*. *coli* strain BW25113 was obtained from the Coli Genetic Stock Center (CGSC) (strain 7636) and was grown at 30°C in LB broth. *Mesoplasma florum* strain L1 (ATCC 33453) was grown at 34°C in ATCC1161 medium containing 200 U/mL penicillin. *Saccharomyces cerevisiae* strain VL6-48 (ATCC MYA-3666) was grown at 30°C in Yeast Extract-Peptone-Adenine-Dextrose (YPAD) medium with 2% glucose. All strains were preserved at -80°C in their respective growth medium containing 25% (vol/vol) glycerol. Batch cultures were grown from frozen stocks in an orbital shaker incubator and used to inoculate VCCD culture vessels (55 mL PYREX tubes, see CS-L1 in S2 Table). VCCD cultures were grown in the same conditions as described for batch cultures in a temperature-controlled chamber.

### VCCD hardware

Complete construction, assembly and operation instructions of the VCCD are regrouped in S1 Manual (user manual). All frame, culture system, and electronics parts were purchased from various sources and are listed in S1–S3 Tables, respectively. Reference codes for every VCCD components are listed in S1–S3 Tables and are used for part identification in S1–S8 Figs. VCCD Frame parts listed in S1 Table were designed using SolidWorks education edition 2014 and can be machined as described in S1 Appendix using standard academic or commercial fabrication services. Machined Frame parts are also available in 3D CAD file format in S1 File. Frame and culture system assemblies are depicted in S1 and S2 Figs, respectively. Electronics schematic diagrams (S5 and S6 Figs) and Printed circuit boards (PCB) layouts (S7 and S8 Figs) were designed using the National Instruments (NI) Circuit Design Suite 11.0 software. PCBs were created using double-sided presensitized copper clad boards and a Kinsten KVB-30 UV exposure box according to manufacturer’s specifications (see EL-A1, FR-O1 to O3, and FR-P1 to P3 in S3 Table). Soldering of components through PCBs holes (see S3 Table, S7 and S8 Figs) was done by hand using a Weller EC1002 soldering iron and Kester 44 Rosin Sn63Pb37 0.031” diameter solder wire (Digi-Key KE1102-ND). Transmittance data acquisition is performed by a NI USB-6008 DAQ (see EL-C1 in S3 Table) and reported to the VCCD software by a 3.0 USB port. Assembly of the electronics box is depicted in S3 and S4 Figs An example of the complete VCCD assembly is available in 3D CAD file format in S2 File (assembly without electronics details).

### VCCD software

VCCD software (version 1.0) was designed using the NI LabVIEW Professional Development System 2009 SP1 software. Executable version of the software (VCCD 1.0.exe) and its dependencies were compiled in an installation package using the LabVIEW 2009 application builder. The installation package and the source code of the software are available at: http://lab-rodrigue.recherche.usherbrooke.ca/VCCD_en/#Software_Download

Current version of the VCCD software was tested on Windows 7 Enterprise Service Pack 1 (64 bits).

### VCCD calibration

VCCD calibration procedure is described in S1 Manual. Briefly, the system was first calibrated by setting the 0% transmittance to correspond to total obscurity. LB broth, water or YPAD medium with 2% glucose were used to set the 100% transmittance value in experiments involving *E*. *coli*, *M*. *florum*, and *S*. *cerevisae* respectively.

### Phenol red as a pH indicator for growth measurement

25 ml of ATCC 1161 medium was progressively acidified by adding increasing volumes of HCl 1N. Transmittance at 560nm was measured at 34°C using the VCCD system calibrated with water for 100% transmittance and total obscurity for 0% transmittance. Acquisition rate and averaging parameters were set to 200 msec and 5 data points, respectively. pH was measured using a VWR SB20 SympHony pH meter calibrated at 34°C.

### Batch culture monitoring

30 mL of *E*. *coli*, *M*. *florum*, and *S*. *cerevisae* were separately grown in a VCCD culture chamber and 560nm transmittance was monitored using the VCCD system calibrated with the appropriate solutions. Growth of *E*. *coli* and *M*. *florum* was monitored with the acquisition rate and averaging parameters respectively set to 1 sec and 5 data points, while 5 sec and 1 data point were used for *S*. *cerevisae*. For the *E*. *coli* culture, absorbance at 600nm was also measured periodically by a GE Healthcare Ultrospec 2100 pro UV/Visible Spectrophotometer calibrated with LB broth. To calculate cell concentrations, 10 μL aliquots were taken at different transmittance values throughout the experiment and then diluted serially with the appropriate culture medium. Dilutions were plated in triplicates on LB, ATCC 1161 with 200 U/mL penicillin or on YPAD 2% glucose plates for *E*. *coli*, *M*. *florum*, and *S*. *cerevisae* respectively. Plates were incubated at the appropriate temperature until colonies were visible. Colonies were counted and colony-forming units (CFU) were calculated according to the corresponding dilution.

### Continuous culture experiments

20 mL of *E*. *coli*, *M*. *florum*, and *S*. *cerevisae* were grown in VCCD culture chambers and 560nm transmittance was monitored using the VCCD system calibrated with the appropriate solutions. Growth of *E*. *coli* and *S*. *cerevisae* was monitored with the acquisition rate and averaging parameters respectively set to 5 sec and 1 data point, while 0.5 sec and 2 data points were used for *M*. *florum*. *E*. *coli* and *S*. *cerevisae* cultures were refreshed using the Threshold-activated mode with a minimum transmittance set to 50% and a pinch time set to 3 sec for 1 cycle. *M*. *florum* culture was refreshed using the Real-time feedback loop mode with minimum and a maximum transmittance values set to 11.5% and 12%, respectively. For all experiments, the refresh flow rate was previously adjusted to approximately 1 mL/sec using sterilized water instead of fresh medium. Cell concentrations were calculated as described in the batch culture monitoring section.

## Results

### VCCD fabrication and principle of operation

**Fig 1:**
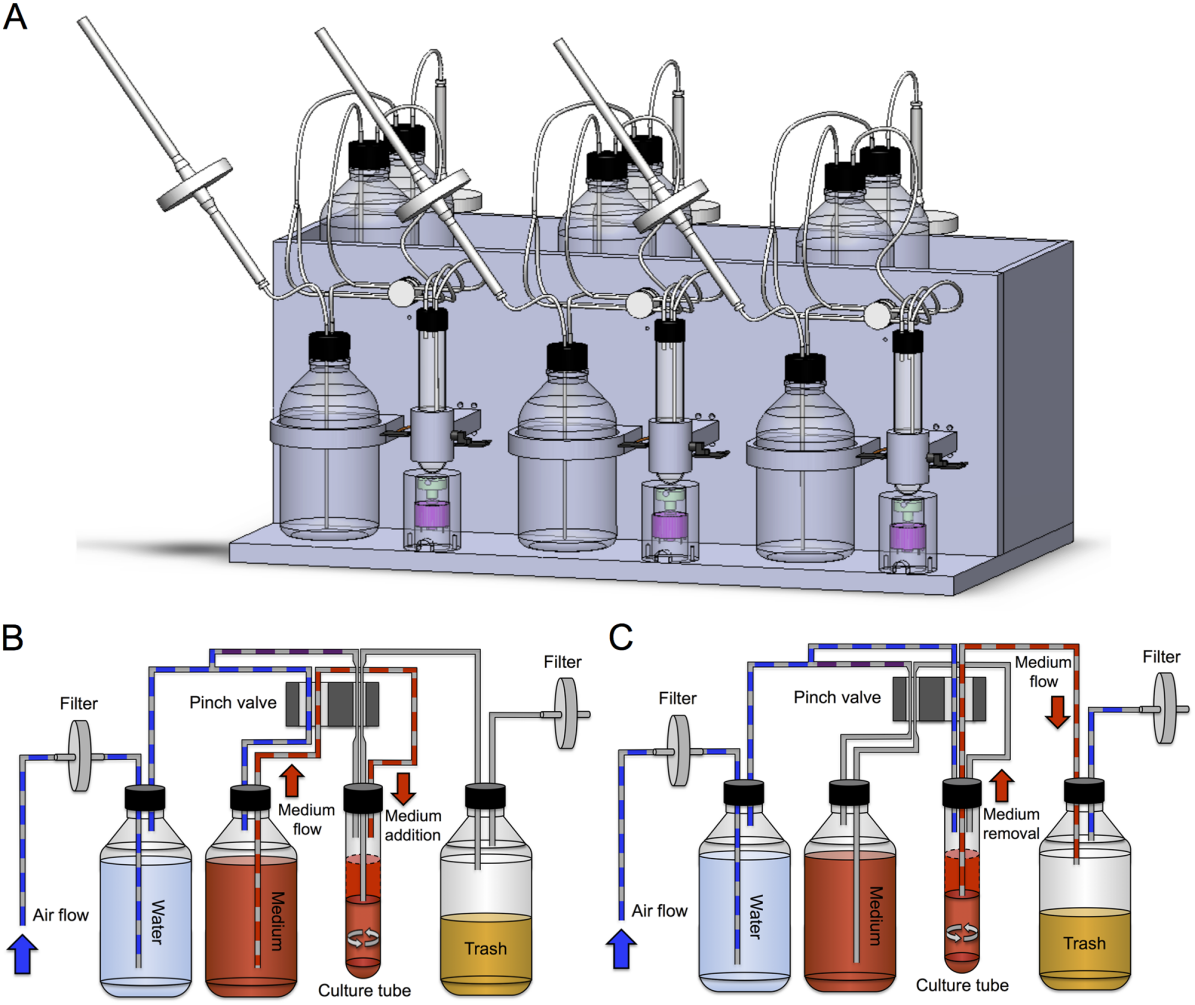
Hardware configuration and schematic depiction of culture-refreshing steps. (A) Three-dimensional representation of the versatile continuous cultivation device (VCCD). The system consists of three independently controlled continuous culture units supported by a plexiglass acrylic structure. For each unit, transmittance is measured through the culture chamber by a light-emitting diode coupled to a photo receiver and then reported to a user interface control system. The culture refreshing capability is provided by computer-controlled pinch valves that manage air and liquid flows inside each culture unit. (B) First step of a culture refresh cycle (culture dilution). Upon pinch valve activation, two tubes of the culture unit get pinched, and the air flow is diverted to the medium bottle resulting in the addition of medium into the culture tube. (C) Second step of a culture refresh cycle (excess culture removal). By returning the pinch valve to its original position, two tubes of the culture unit are pinched, which then redirects the air flow to the culture tube and causes the excess of volume to be evacuated into the trash bottle.

We developed the VCCD, a low-cost and open-source device composed of three independently controlled continuous culture units mounted on a plexiglass acrylic frame (Fig 1A, S1 and S2 Figs). Each culture unit includes a culture vessel whose turbidity is measured using a conventional 560nm light emitting diode (LED) paired with a photo receiver (PHR). Transmittance at 560nm is monitored in real-time while cultures are gently agitated with adjustable speed magnetic stir bars. To establish continuous cultures, silicone tubing connects each culture vessel to an adjustable air pump that provides a low air pressure sufficient for liquid displacement inside the system (Fig 1A and 1B, S1 and S2 Figs). The culture vessel is connected between a bottle containing fresh medium (in which water saturated air is injected to displace the liquid when needed) and a trash bottle. The silicone tubing network passes through a four way computer-controlled pinch valve that, once specified conditions are reached, changes the air flow path resulting in culture dilution (Fig 1B). When culture dilution is completed, the pinch valve returns to its original position and the excess volume is removed and disposed inside the trash bottle (Fig 1C). The entire continuous culture unit is flanked by two 0.2 μm filters, and can be autoclaved for sterilization (see S1 Manual). With the proposed frame configuration (see S1 Appendix), the VCCD is most likely to be used in a temperature-controlled room, but could easily be adapted to fit in a common incubator.

Electrical alimentation of LEDs, PHRs, mixing motors and pinch valves are supported by low-cost PCBs that can be easily fabricated and assembled using standard procedures (S3 Table, S3–S8 Figs). Transmittance measurement components were designed to use circular connectors so that different sets of LEDs and PHRs could be rapidly exchanged without requiring any specialized tool. Since the frequency of light emission of LEDs and the acquisition rate of PHRs are synchronized, the impact of ambient light on the PHR is nearly abolished, thus eliminating the need to use the VCCD in total obscurity to acquire accurate transmittance signals. Data acquisition is performed by a multifunction NI USB-6008 DAQ (see EL-C1 in S3 Table) connected by a standard 3.0 USB port to a computer executing the VCCD software, which is used to display transmittance signal graphs and control culture dilution cycles.

**Fig 2:**
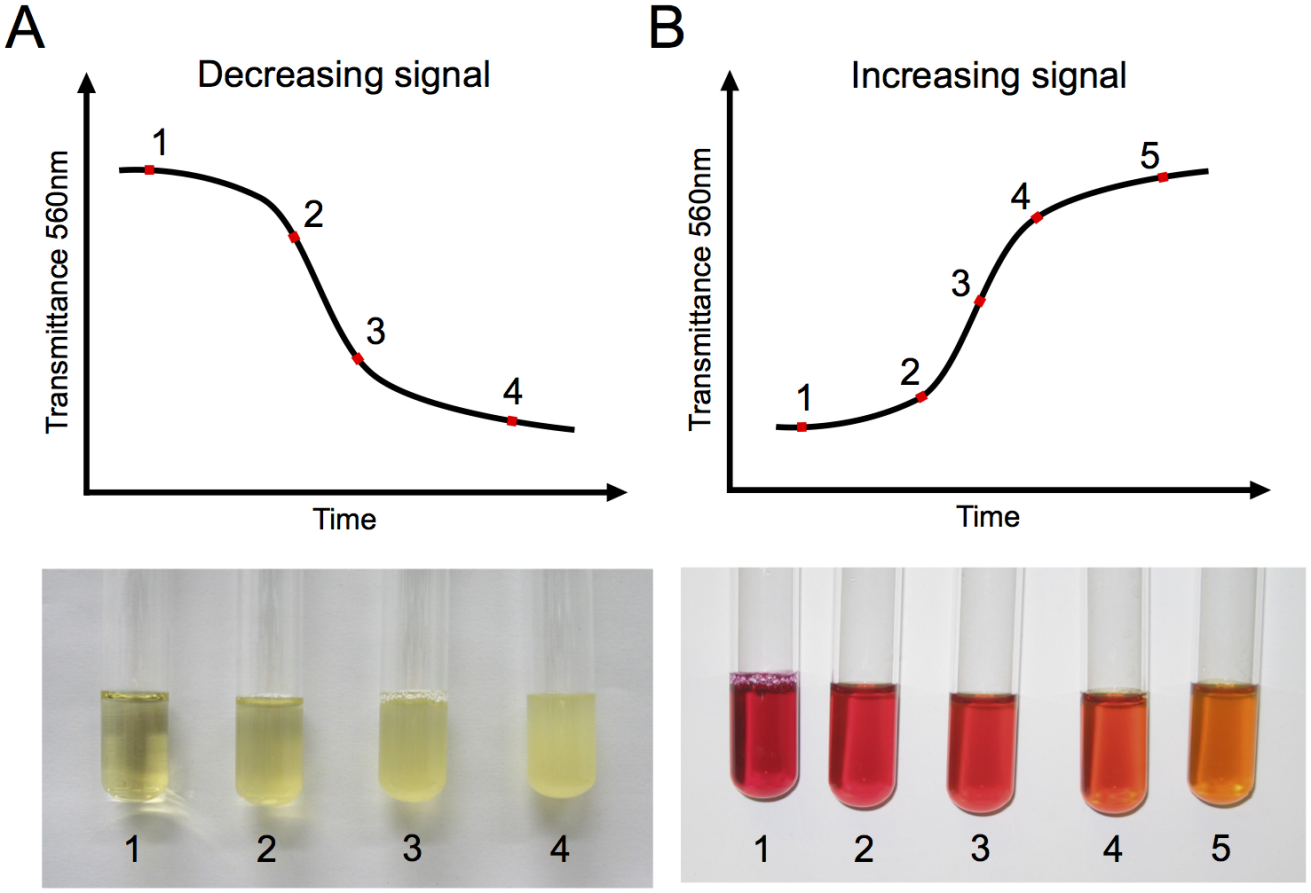
Typical transmittance curves associated with bacterial growth. (A) Increasing opacity observed when monitoring transmittance of an *E*. *coli* culture in LB broth, where the growing cells increasingly block the incident light generated by a light emitting diode. (B) In some cases, growth can alternatively be measured by adding phenol red to the growth medium as a pH indicator. This method is especially employed to follow the growth of *M*. *florum* in ATCC 1161 medium, where medium acidification caused by metabolic activity is reported by an increase in the 560nm transmittance.

In general, culture transmittance declines as the cell population grows and gradually blocks the incident light. This phenomenon is due to an increase in turbidity that is easily visible when following the growth of *E*. *coli* in LB broth (Fig 2A). However, some microorganisms do not possess the physiological characteristics required to proportionally increase culture opacity when growing. For instance, very small cells (<500 μm) such as mollicutes (e.g. *Mycoplasma sp* and *Mesoplasma sp*) do not absorb or scatter light efficiently, and as a consequence, are not properly quantified using a turbidity based approach [23,24]. Under these circumstances, our selected LED and PHR allow using phenol red as a growth medium pH indicator to monitor the acidification caused by metabolic activity and proliferation of cells. As pH decreases, the absorbance of phenol red at 560 nm drops (which results in a concomitant increase of the 560nm transmittance), and is visible through a color change of the medium from red to orange (Fig 2B).

### The VCCD can measure the growth of various microorganisms

**Fig 3:**
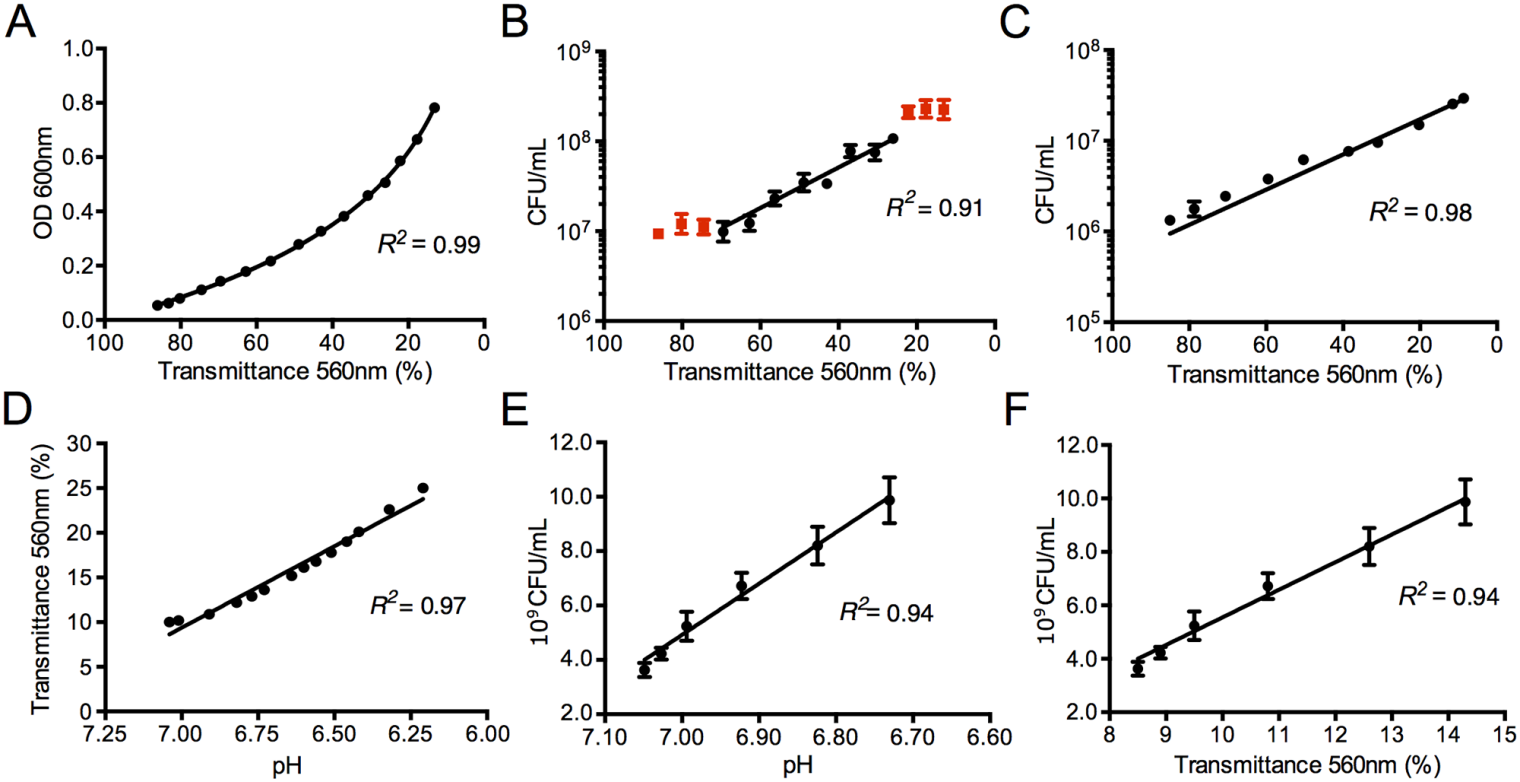
Calibration of the Versatile continuous culture device (VCCD) using batch cultures. (A) Comparison of 560nm transmittance measured by the VCCD and 600nm absorbance measured by a conventional spectrophotometer of an *E*. *coli* culture in LB broth. (B) Relationship between relative 560nm transmittance measured by the VCCD and cell density of an *E*. *coli* culture grown in LB broth. Red squares are excluded from the correlation determination because they are not part of the exponential growth phase. (C) Relationship between relative 560nm transmittance measured by the VCCD and cell density of *S*. *cerevisae* growing in YPAD 2% glucose medium. (D) Relative 560nm transmittance of ATCC 1161 medium through different pH values generally observed during *M*. *florum* growth. (E) Cell density of *M*. *florum* growing in ATCC 1161 medium through pH decrease. (F) Relationship between relative 560nm transmittance measured by the VCCD and cell density of an *M*. *florum* culture grown in ATCC 1161 medium.

In order to use the VCCD to keep cell populations at a constant density for long periods, we first verified that the instrument could accurately measure the growth of different model microorganisms. Using the VCCD to monitor the growth of an *E*. *coli* BW25113 [25] batch culture in LB broth, we observed a decreasing 560nm transmittance signal as cells grew (S9A and S10A Figs), and more importantly, we noticed a strong correlation according to the Beer-Lambert law between 560nm transmittance values acquired by our system and 600nm absorbance measured by a conventional spectrophotometer (Fig 3A). This suggests that transmittance quantification by our LED-PHR approach offers performances comparable to commercially available instruments, even for measurements performed under ambient light. We also observed a strong linear correlation between log of cell concentrations and relative 560nm transmittance ranging from approximately 10^7^ to 10^8^ CFU/mL and 70% to 25%, respectively (Fig 3B). This range of transmittance roughly corresponds to an OD_600nm_ signal between 0.2 and 0.5 (Fig 3A), an optical density interval typically associated with *E*. *coli* exponential growth phase. A similar pattern was also observed with *S*. *cerevisae* growing in YPAD medium with 2% glucose (Fig 3C, S10C and S10D Fig), clearly showing that the selected LED and PHR for the VCCD are suitable to follow the growth of commonly studied microorganisms.

We then sought to establish that our system is suitable to monitor the growth of microorganisms whose characteristics impose the use of phenol red as a growth indicator. To do so, we measured the transmittance at 560nm of ATCC 1161 medium employed to grow diverse bacteria belonging to the class of mollicutes, and observed that the color change caused by acidification of the medium was accurately detected by the VCCD. A linear correlation was observed between transmittance at 560nm and pH from ~6 to 7 (Fig 3D). Next, we monitored the growth of a batch culture of *M*. *florum*, a mollicute closely related to mycoplasmas [26,27]. We observed that the transmittance signal followed a typical pattern for this bacterium (S9B and S10E Figs), in which 560nm transmittance initially increases from about 8% to 16% and then decreases due to cell agglomeration. Importantly, medium acidification caused by metabolic activity, as well as measured transmittance at 560nm, were very well correlated with cell concentrations from approximately 10^9^ and 10^10^ CFU/mL (Fig 3E and 3F), showing that our device is suitable to indirectly monitor the growth of microorganisms using phenol red.

### Available culture refresh modes

After establishing a strong correlation between cell density and measured 560nm transmittance for different microorganisms, we investigated whether the VCCD could maintain cell populations at a desired concentration for extended periods. To achieve this goal, we first verified that our continuous culture system configuration could accurately dilute cell population to perform culture refreshing. As expected, volume of liquid added to the culture vessel was well correlated with pinch valve opening time (S11 Fig), at a flow rate of ~1 mL/sec in our assay. Because selected culture vessels have a maximal volume capacity of 55 mL, we designed our system to support cultures of about 20 mL to allow the possibility of diluting them in half in a single refresh cycle. If a greater dilution factor is required, multiple dilution cycles could be programmed with the software to avoid any overflow (see below).

**Fig 4:**
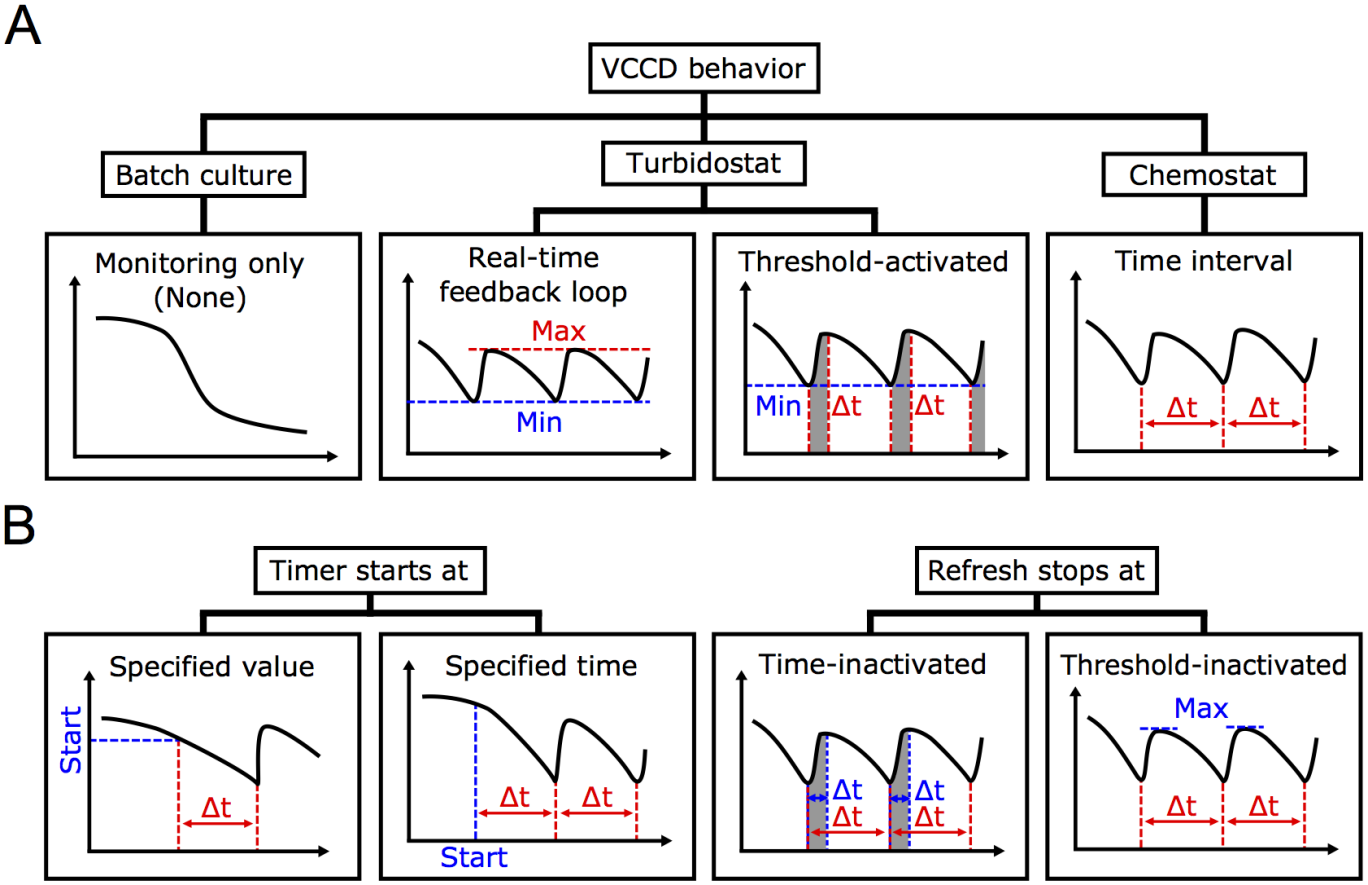
Illustration of available continuous culture modes used to maintain cell growth. (A) Under its present software configuration, the versatile continuous cultivation device (VCCD) can behave like a turbidostat or a chemostat, or simply be used to measure the transmittance of a batch culture without performing any culture refresh. In the turbidostat mode, the culture is refreshed when a desired transmittance value is detected until: 1) a second value is reached [Real-time feedback loop] or 2) a specified refresh time has elapsed [Threshold-activated]. Alternatively, the chemostat behavior uses a Time interval mode to refresh the culture with a constant specified dilution rate. (B) In the Time interval mode, additional options are available to choose if the interval timer starts at a specified value or at a specified time, and to decide if refreshes are stopped in a time-dependent manner or using a transmittance threshold.

In order to establish continuous cultures, we included different options and parameters in the VCCD software to accommodate various experimental parameters. Among those, one major setting that has to be considered before executing a continuous culture experiment is the desired general behavior of the system, which is defined according to the selected culture refresh mode (Fig 4A). For example, the Real-time feedback loop mode makes the VCCD behave like a turbidostat, in which the culture is diluted by fresh medium in response to a specific transmittance threshold until a second threshold is reached. In that mode, a maximum culture refresh time (pinch time) can be set to avoid culture overflow during a refresh cycle, thus forcing the system to execute multiple dilution cycles to obtain the desired transmittance value (see S1 Manual). If a chemostat behavior is more convenient for a specific experiment, the Time interval mode can be chosen to refresh the culture with a constant dilution rate. In that context, different options must be selected to specify whether the interval timer starts at a specified transmittance value or at a specified time, and if culture refreshing is stopped according to a defined pinch time or a transmittance threshold (Fig 4B).

### The VCCD can establish continuous cultures of different microorganisms

**Fig 5:**
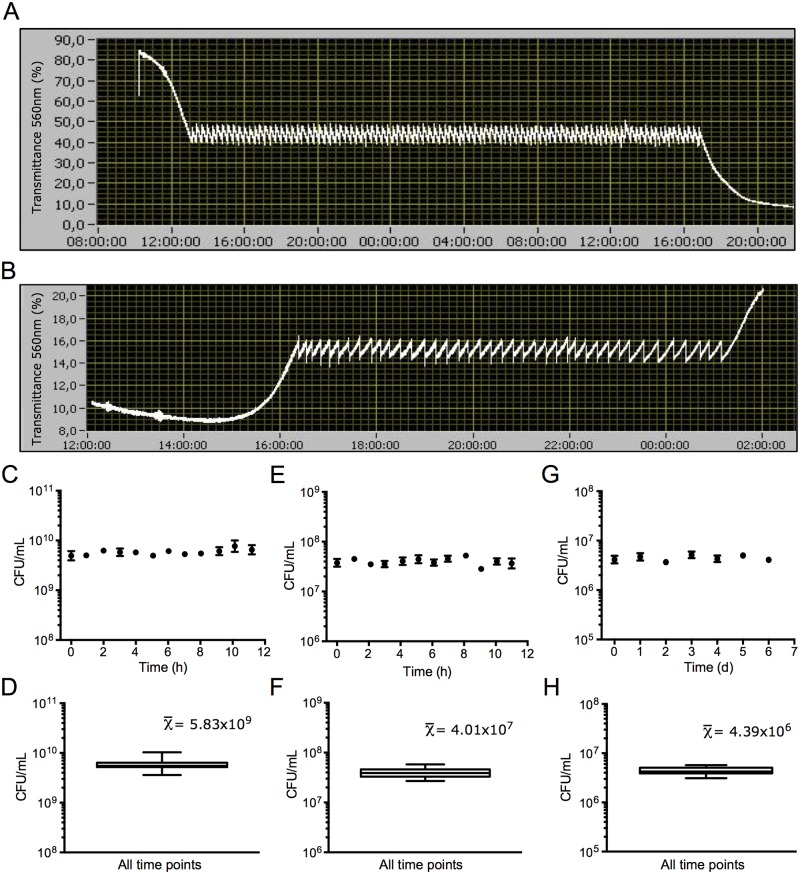
Establishment of continuous cultures using the versatile continuous culture device (VCCD). Example of transmittance curves monitored by the VCCD of an *E*. *coli* culture growing in LB broth (A) and a *M*. *florum* culture growing in ATCC 1161 medium (B) maintained for several hours at 40% and 16% of transmittance, respectively. Culture refreshes were performed using the Real-time feedback loop mode. Cell concentrations measured throughout continuous culture experiments carried out on *M*. *florum* (C), *E*. *coli* (E), and *S*. *cerevisae* cultures (G) using different culture refresh modes, as well as box and whiskers plots of the whole experiments (D), (F), and (H) respectively.

To verify if our VCCD modes can effectively maintain cell populations at a desired concentration, we conducted continuous culture experiments on the three microorganisms previously chosen for batch culture monitoring experiments, that is *E*. *coli*, *M*. *florum* and *S*. *cerevisae*. When performing continuous culture experiments on *E*. *coli* and *M*. *florum* using the Real-time feedback loop refresh mode, we observed that both cultures never exceeded the selected transmittance thresholds specified to the VCCD software (Fig 5A and 5B), except when refreshes were manually disabled to let them grow to their maximal density. As expected, a very high stability in cell concentration was obtained for *M*. *florum* continuous culture experiments (Fig 5C and 5D). *E*. *coli* continuous cultures using the Threshold-activated refresh mode provided similar results (Fig 5E and 5F). Furthermore, the VCCD allowed us to maintain a *S*. *cerevisae* continuous culture in tight range of cell concentrations for almost a week (Fig 5G and 5F), and could be used to maintain cell density for longer periods. Taken together, these results demonstrate that the VCCD is capable of reliably maintaining cell culture densities under a narrow and stable range for various microorganisms and under different operating modes.

## Discussion

We have designed the VCCD, a continuous cultivation device that allows reaching and maintaining a constant cell density in a stable growth environment for various types of microorganisms such as bacteria and yeast. The complete documentation to build the VCCD along with the GUI software to operate the system is also provided for any potential user. The system is modular with three growth chambers that can each independently be programmed to operate as a turbidostat or chemostat. The instrument can be built at a relatively affordable cost of ~$1,400 US. We expect that biologists will be able not only to take advantage of the system to perform their experiments but also to customize it according to their experimental requirements and make the modifications publicly available. For example, different growth chamber configurations could be created and alternative sensors could be implemented such as a pH meter, thermometer, dissolved oxygen probe, light meter, etc. These modifications may require an upgrade to a data acquisition card containing additional ports but this would require a minimal effort. The VCCD software was designed with the NI Lab View platform to facilitate such changes and allow the rapid and easy integration of new features. As a first step, a fluorescence acquisition mode that could be useful for the validation of synthetic gene circuits is already included in the software although this functionality has not been tested yet (see S1 Manual). There is clearly ample room to significantly extend the capabilities of the VCCD. This could allow the cultivation of new organisms such as photosynthetic microbes, algae, or insect and animal cells. Ultimately, the VCCD could become an interesting small-scale bioreactor used in metabolic engineering, synthetic biology and in several other disciplines.

In systems biology as well as other fields, reaching stable growth conditions is particularly important when trying to decipher the precise mechanisms of cell functioning. Steady state conditions should greatly reduce interference or noise arising from a constantly fluctuating environment. In principle, steady state conditions can be maintained indefinitely, which could be particularly useful to study the evolution of a cell population under selective conditions or to follow the fate of a group of mutants. However, in practice establishing steady state conditions does not necessarily require extended incubations under constant growth conditions. For example, as shown in Fig 5E, an *E*. *coli* population was maintained at maximal growth rate under controlled growth conditions for ~25 generations over a 12 hour period based on the estimated growth rate in batch culture (S10B Fig). Similarly, *M*. *florum* was subjected to ~20 cell divisions during a 12 hour incubation in the VCCD (Fig 5C and S10F Fig). It is fair to assume that cells can reach a steady state during this incubation, which would be important and achievable within a manageable time frame for systems biology studies. For longer experiments, the use of continuous culture device is also interesting but often limited by biofilm formation on the growth chambers walls, which compromises optical density measurements and dilution operations. This limitation is not specific to the VCCD but could be countered by the use of new coating agents that abolish or significantly reduce biofilm formation [28–30]. Alternatively, users can simply transfer the culture to a new tube to prolong the experiment each time biofilm formation becomes problematic.

In sum, the description and publication of this initial version of the VCCD represents a first step in the development of an open-source laboratory platform for continuous cultivation that we hope will be embraced by the scientific community.

## Supporting Information

S1 Appendix Frame machining details. (PDF)

S1 Fig Frame assembly. (PDF)

S2 Fig Culture system assembly. (PDF)

S3 Fig Electronics box assembly. (PDF)

S4 Fig Assembly of the main electronics components. (PDF)

S5 Fig Schematic diagram of the main board electronics. (PDF)

S6 Fig Schematic diagrams of the electronics (A), and circular connectors (B) of the photo emitter, photo receiver, mixer, and pinch valve. (PDF)

S7 Fig Details of the main PCB. (PDF)

S8 Fig Details of the photo emitter and receiver PCBs. (PDF)

S9 Fig Typical batch culture growth curves displayed on the graphical user interface (GUI) software for *E*. *coli* (A) and *M*. *florum* (B). (PDF)

S10 Fig Bacterial and yeast batch culture growth curves. (PDF)

S11 Fig Volume of liquid added to a culture vessel during a refresh cycle of a specified time. (PDF)

S1 File Frame parts in 3D CAD format. (ZIP)

S2 File Example of a complete VCCD assembly in 3D CAD format. (ZIP)

S1 Manual VCCD User Manual. Contains all construction and assembly instructions, a system and software utilization guide, as well as important operation notes.
(PDF)

S1 Table Frame material list. Contains detailed description for each part belonging to the VCCD frame, as well as their reference code in S1 Fig and S1 Appendix.
(XLSX)

S2 Table Culture system material list. Contains detailed description for each part belonging to the VCCD culture system, as well as their reference code on S2 Fig.
(XLSX)

S3 Table Electronics material list. Contains detailed description for each part belonging to the VCCD Electronics, as well as their reference code on S3–S8 Figs.
(XLSX)
