# Supplementary figures and images for: A Small-Volume, Low-Cost, and Versatile Continuous Culture Device
Source: PLoS One. 2015 Jul 21;10(7):e0133384. doi: 10.1371/journal.pone.0133384 (PMC4510131; doi:10.1371/journal.pone.0133384)

FR-A1

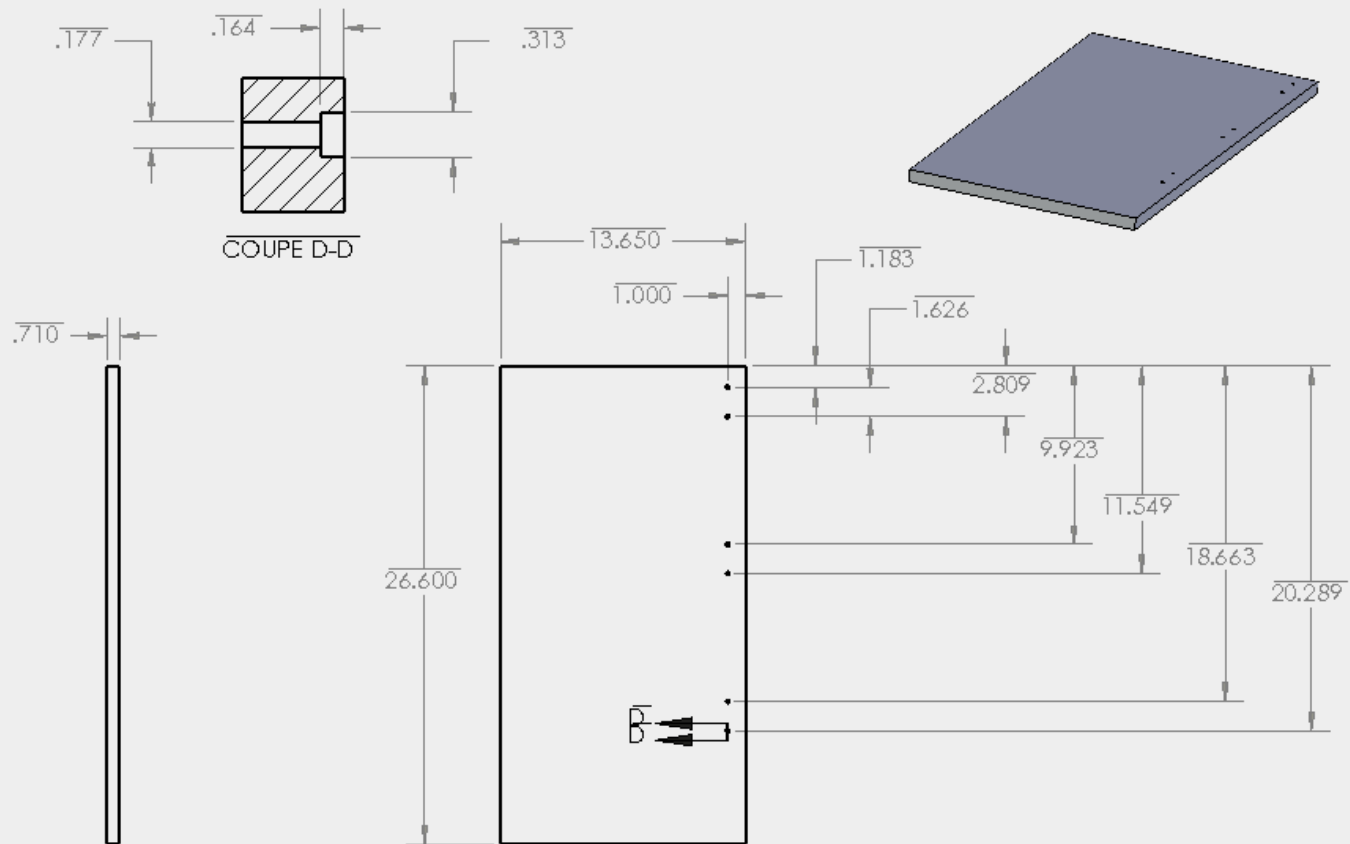

FR-B1

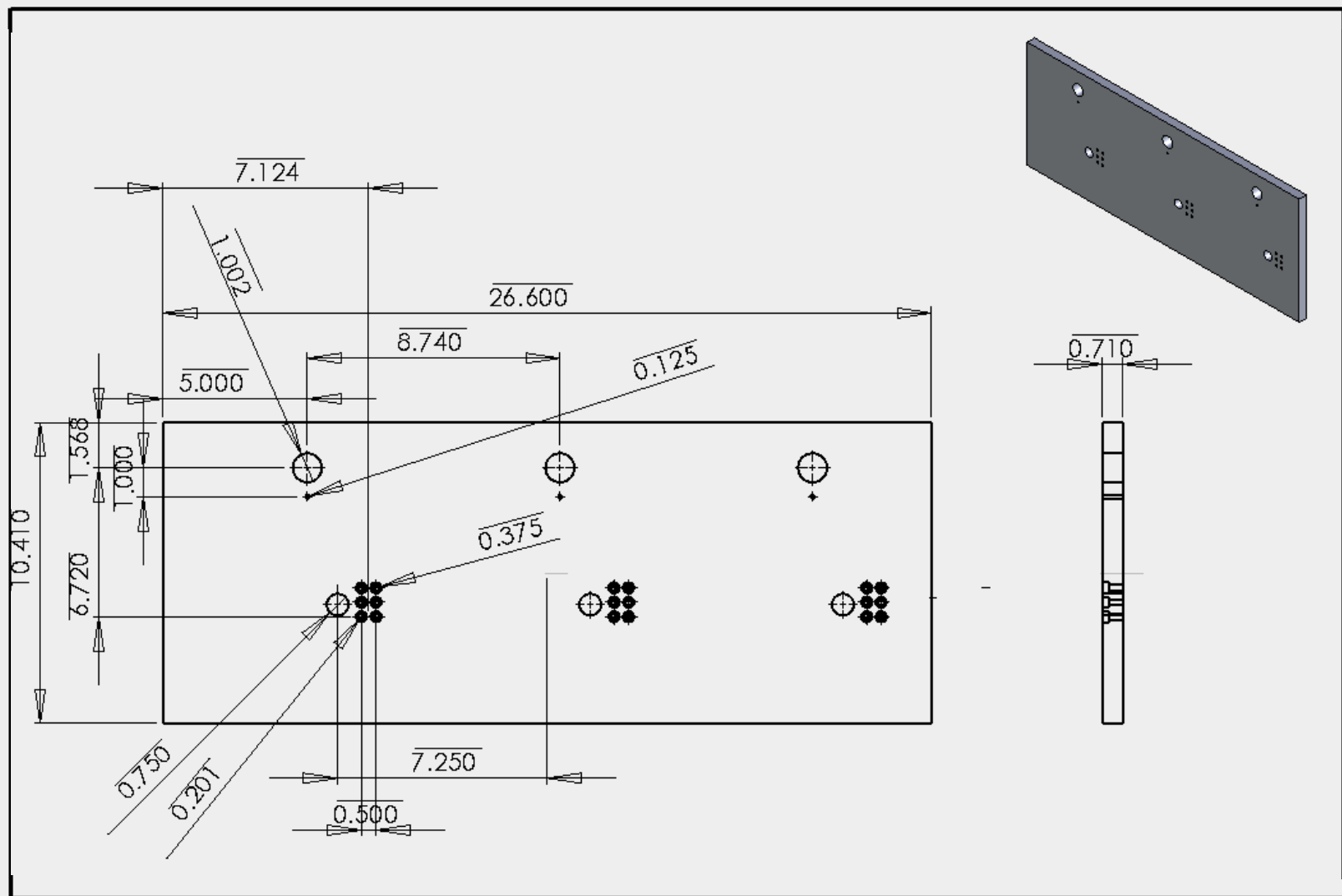

FR-C1

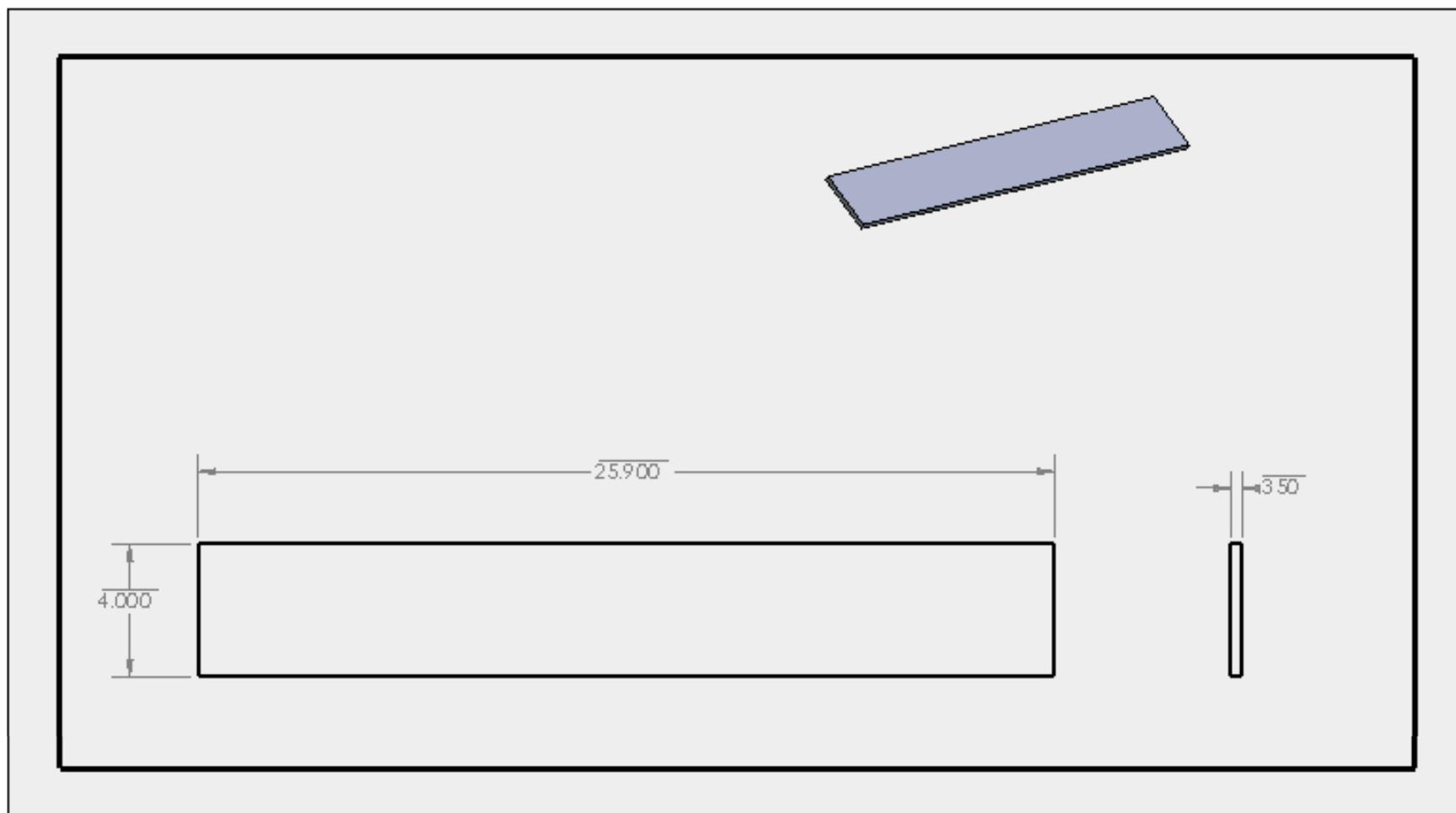

FR-D1; D2

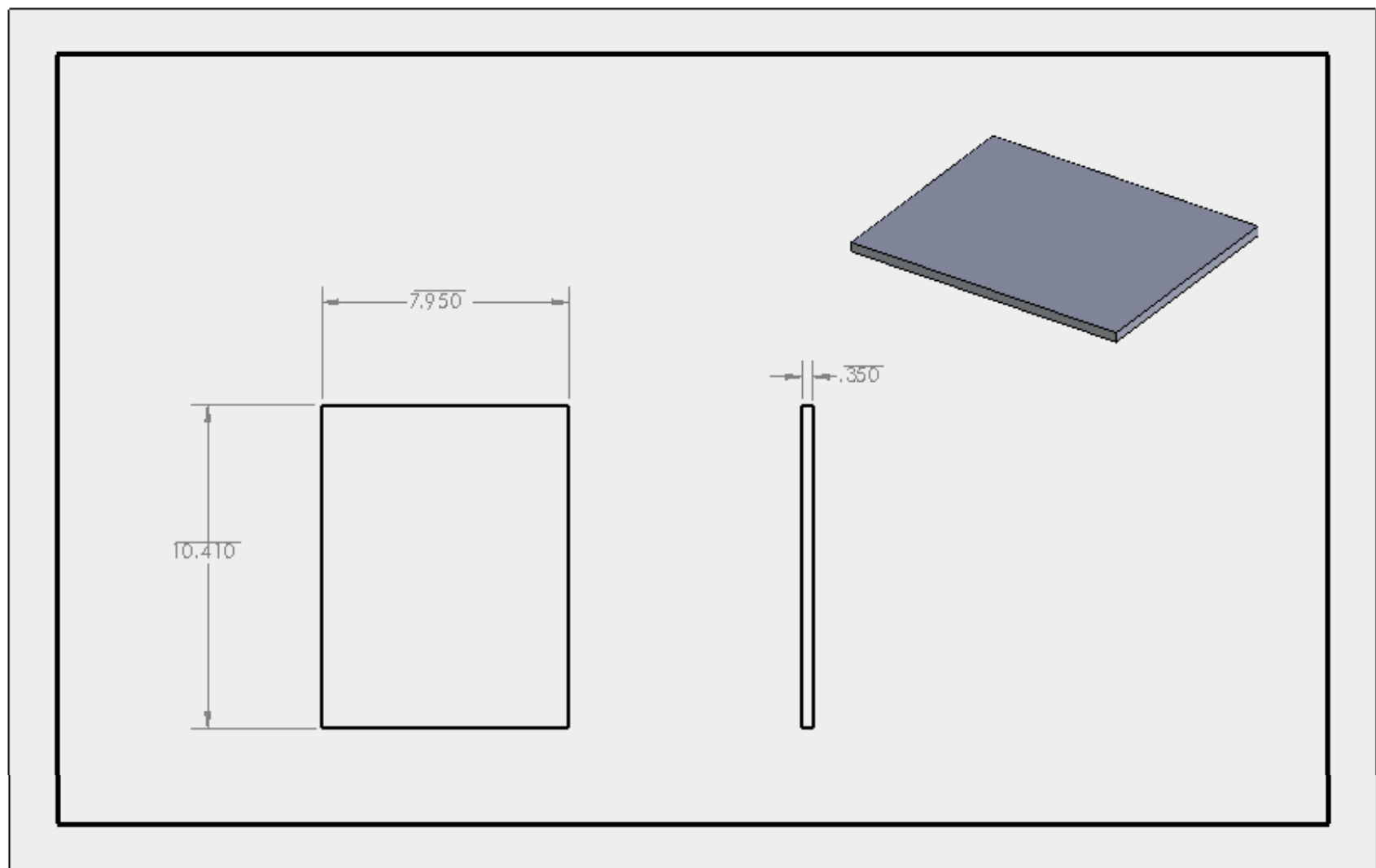

FR-E1

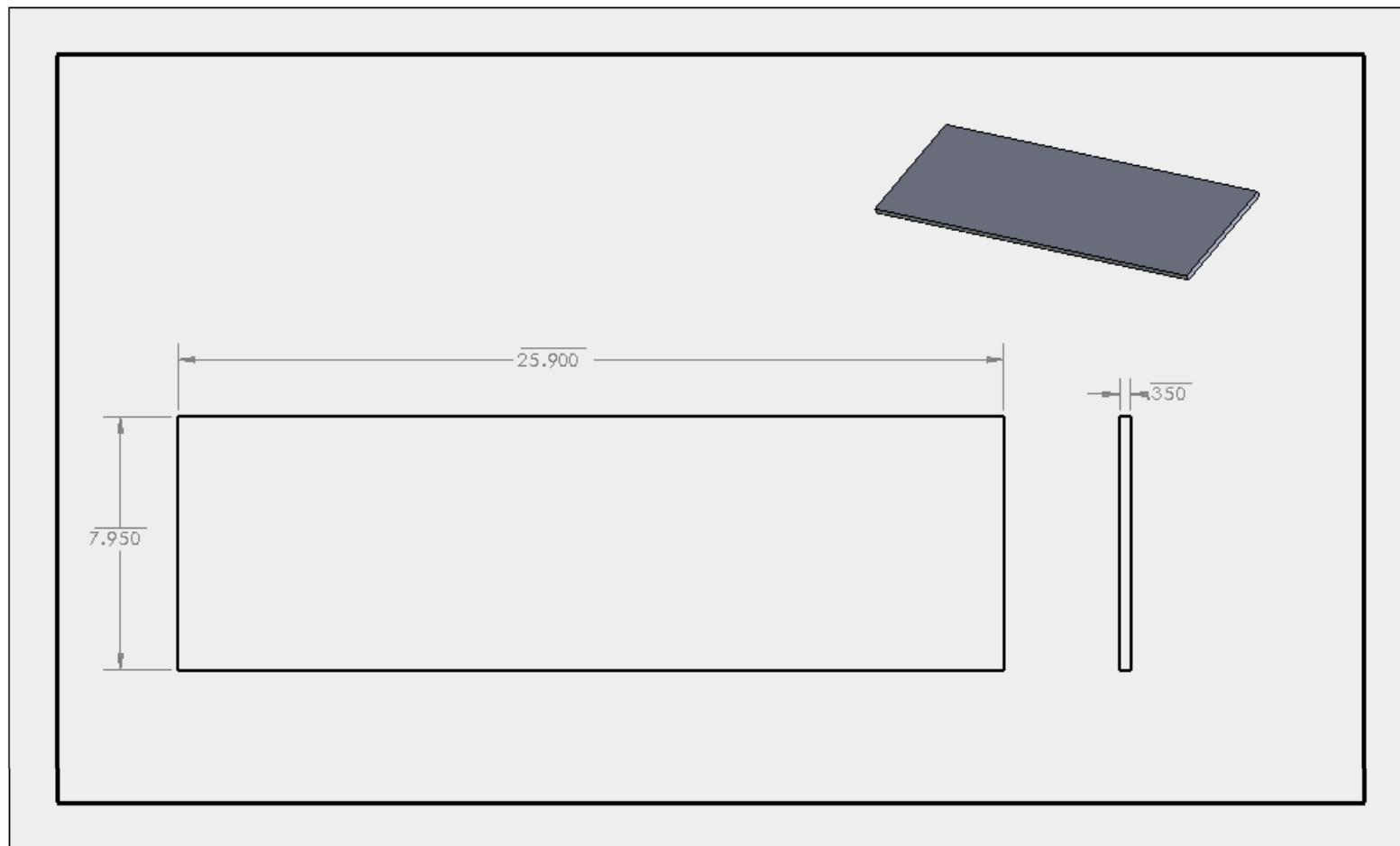

FR-F1

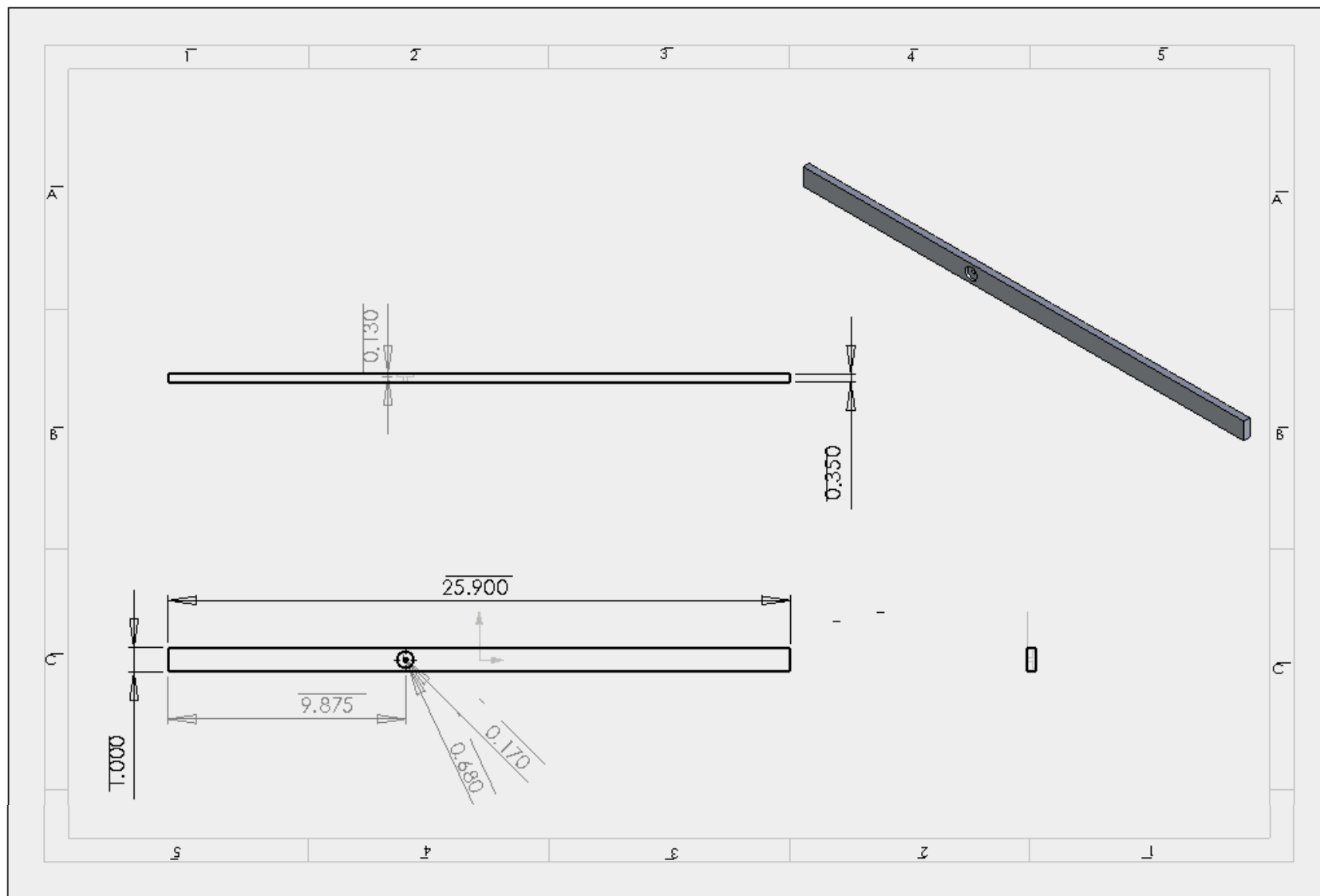

FR-G1; G2; G3

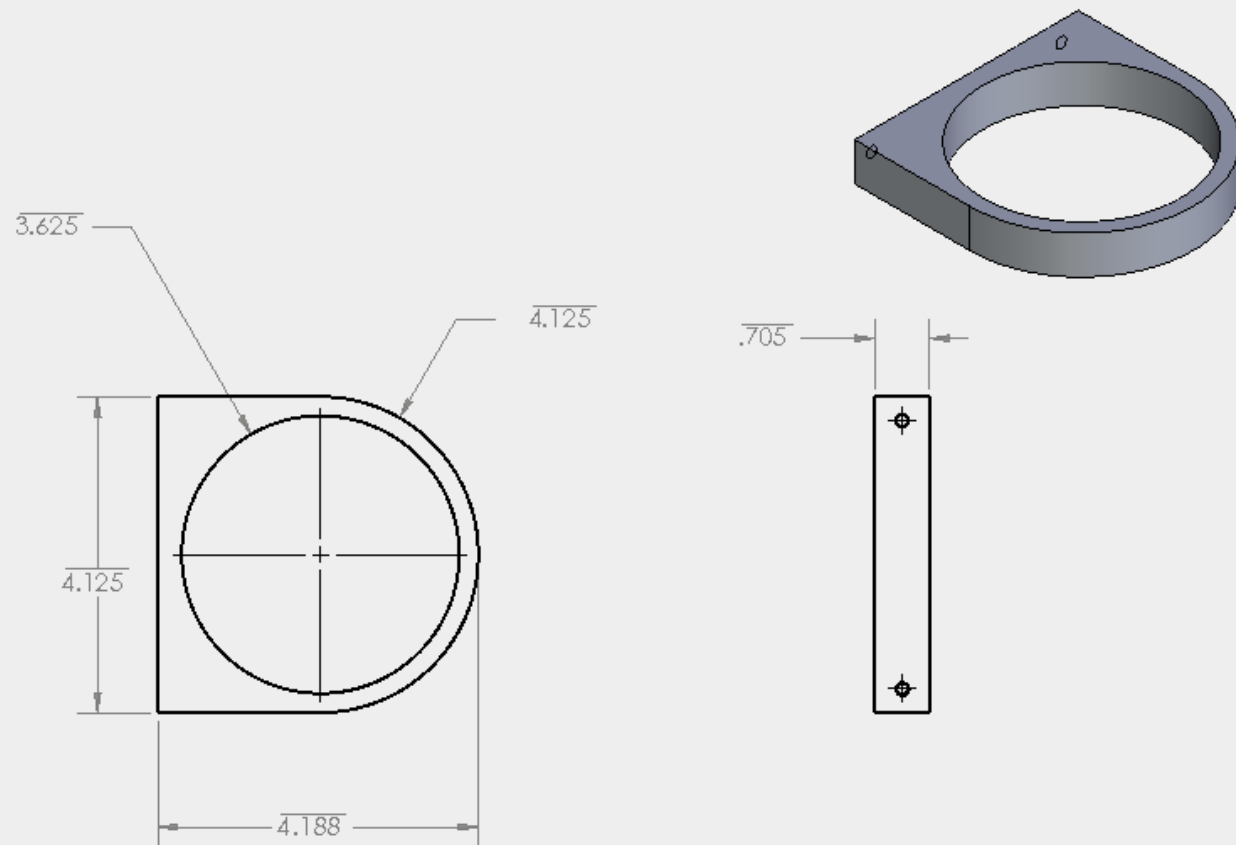

FR-H1; H2; H3

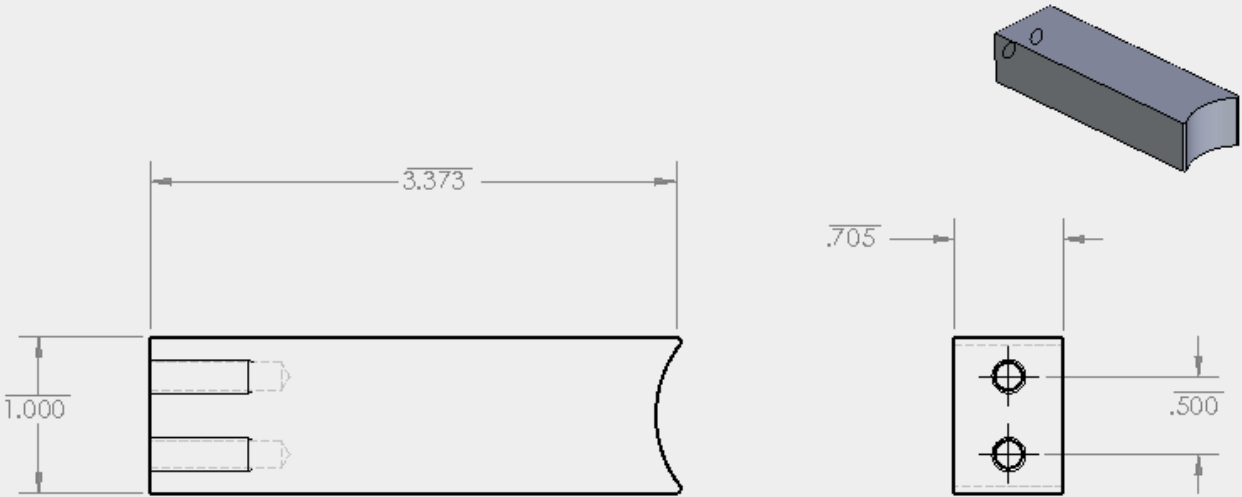

FR-I1; I2; I3

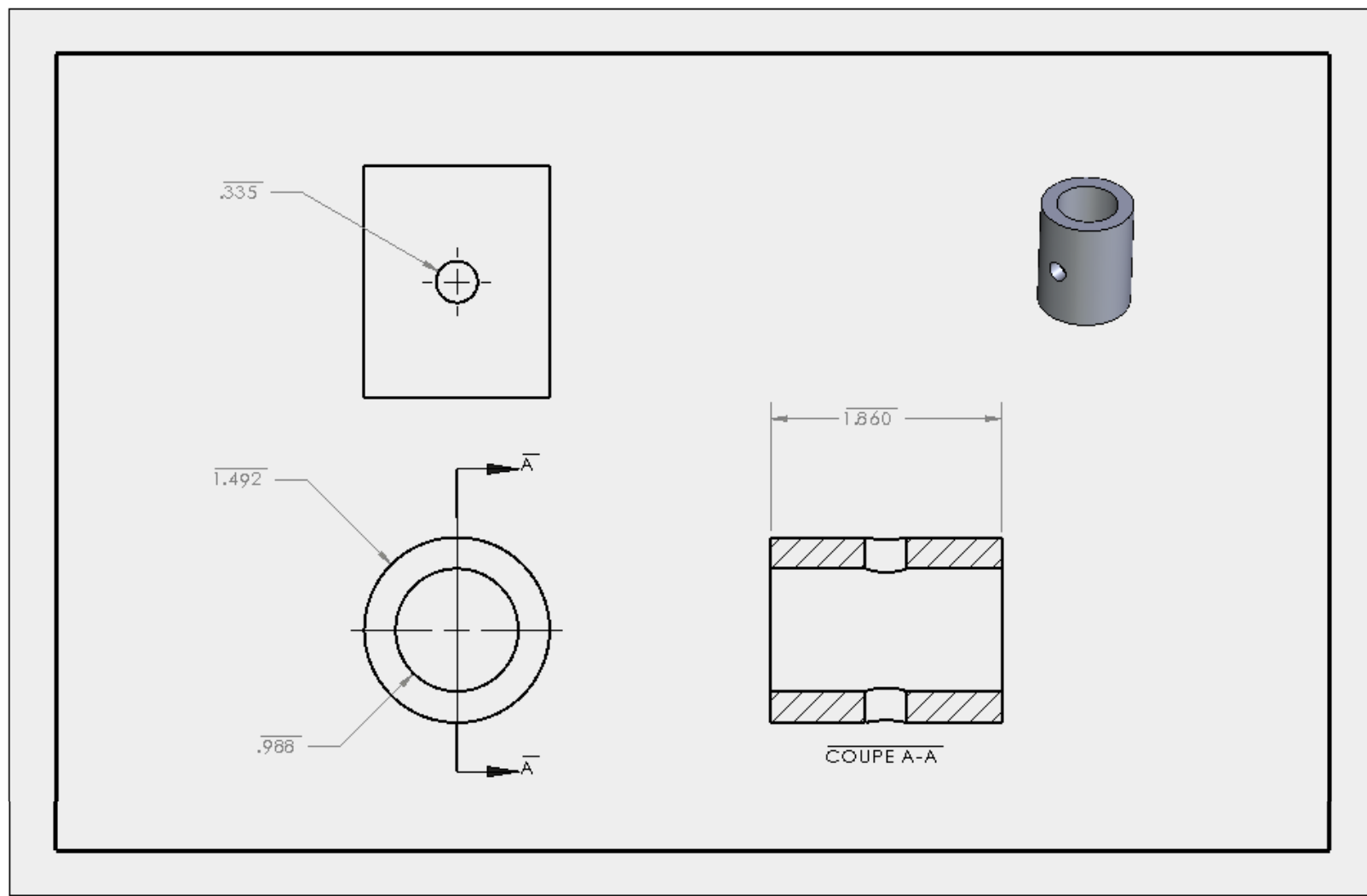

FR-J1; J2; J3

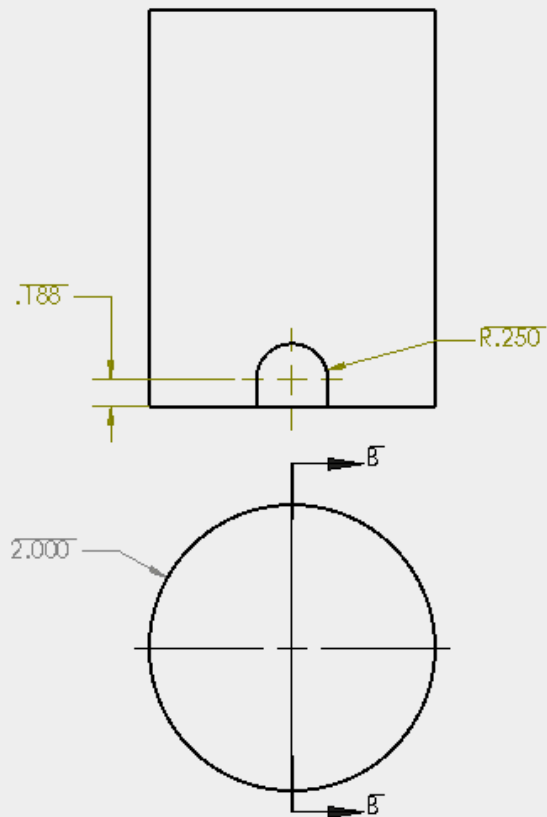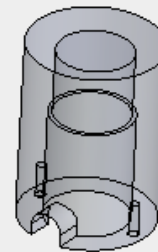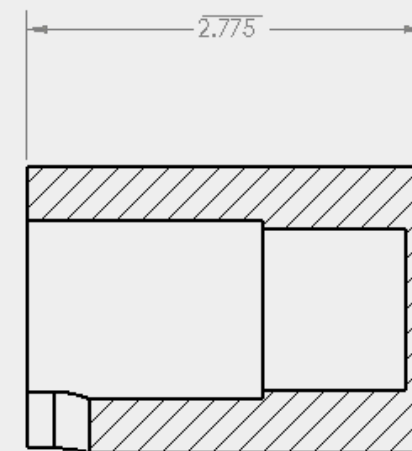

COUPE B-B  
ECHELLE 1 : 1

FR-L1; L2; L3

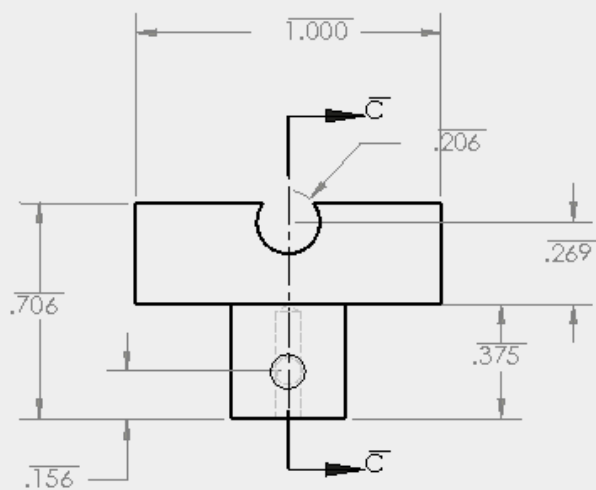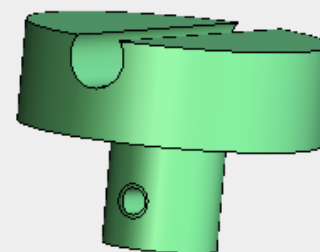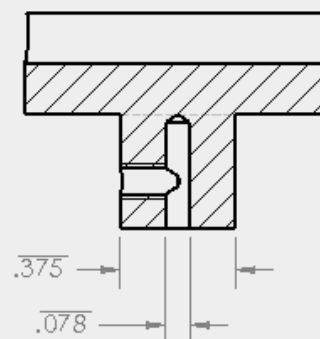

COUPE C-C  
ECHELLE 2:1

Supplement: S1 Appendix — (PDF) [file pone.0133384.s001.pdf]

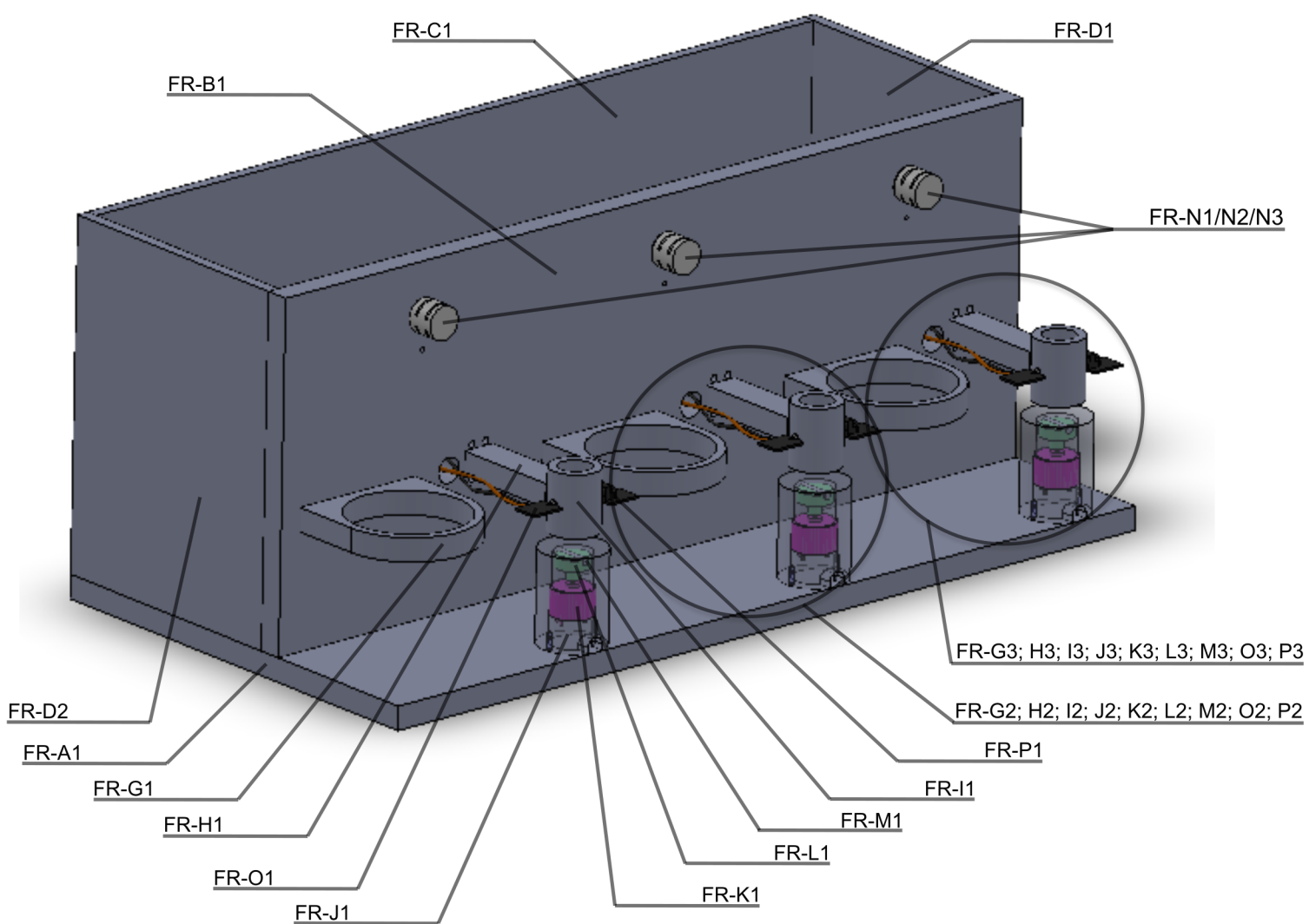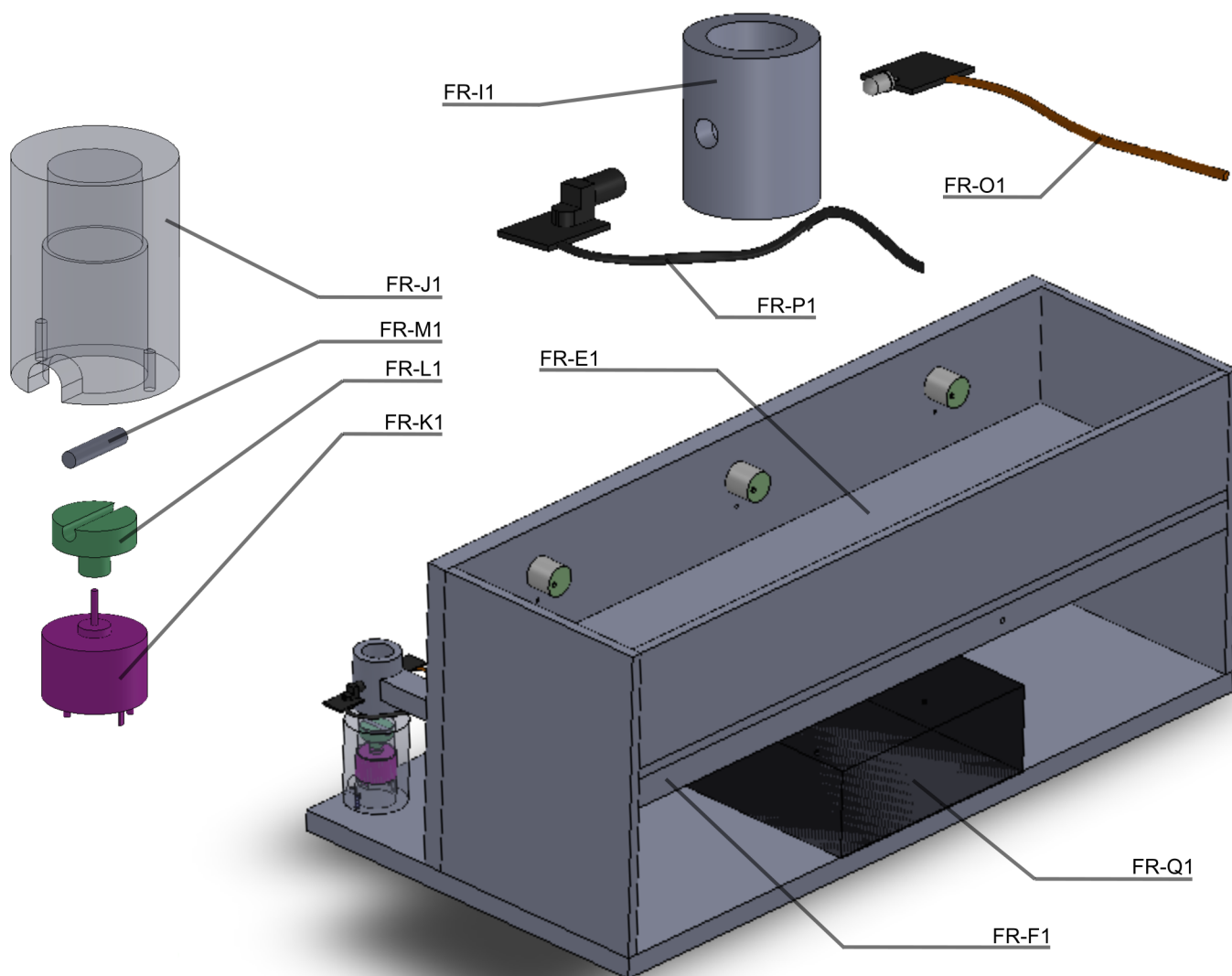

Supplement: S1 Fig — (PDF) [file pone.0133384.s002.pdf]

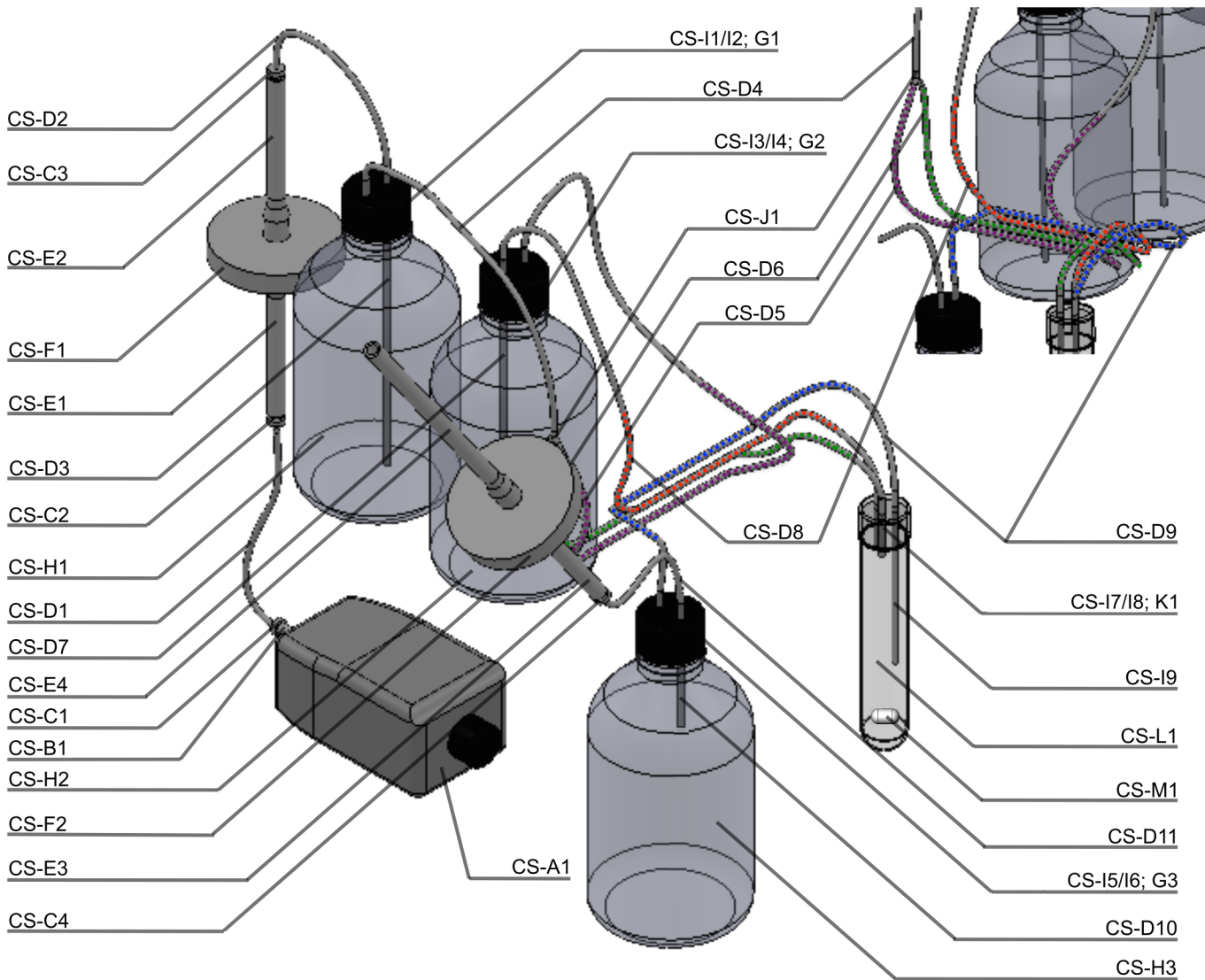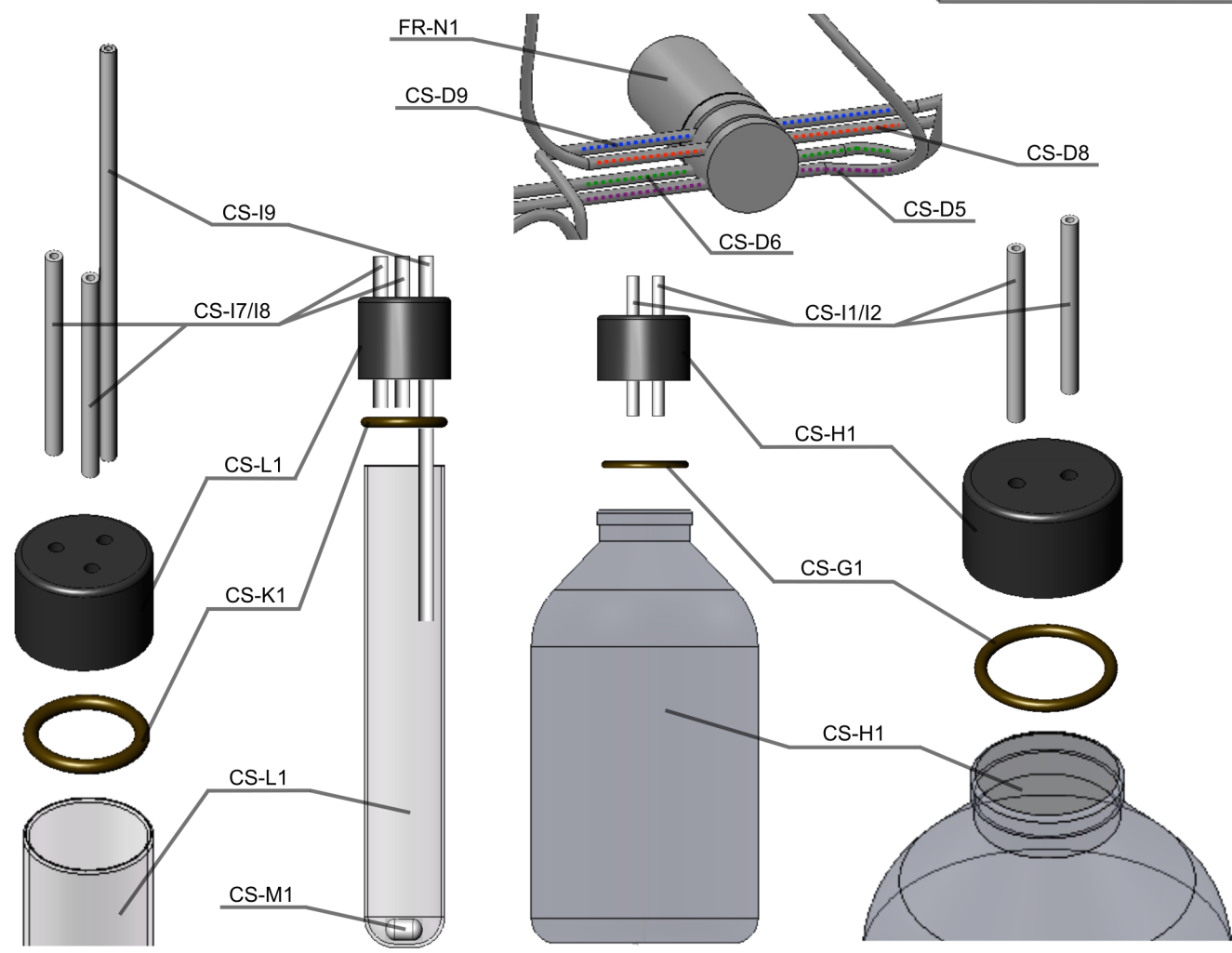

Supplement: S2 Fig — (PDF) [file pone.0133384.s003.pdf]

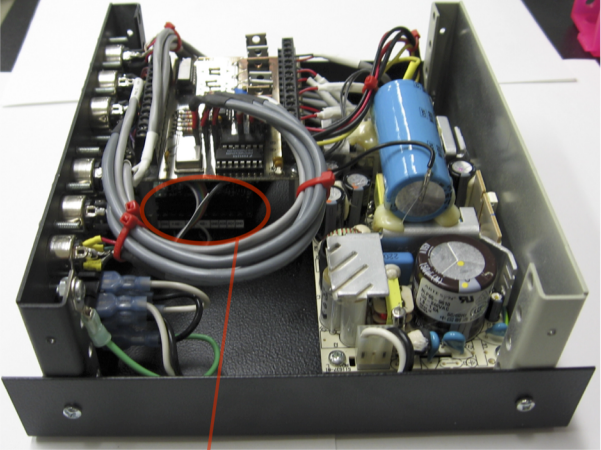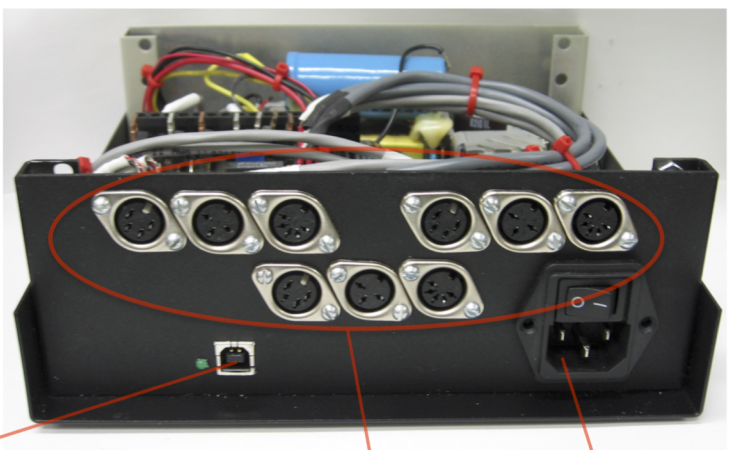

EL-C1

EL-D1

EL-E1/E2/E3; F1/F2/F3; G1/G2/G3

EL-H1

EL-B1

EL-A1

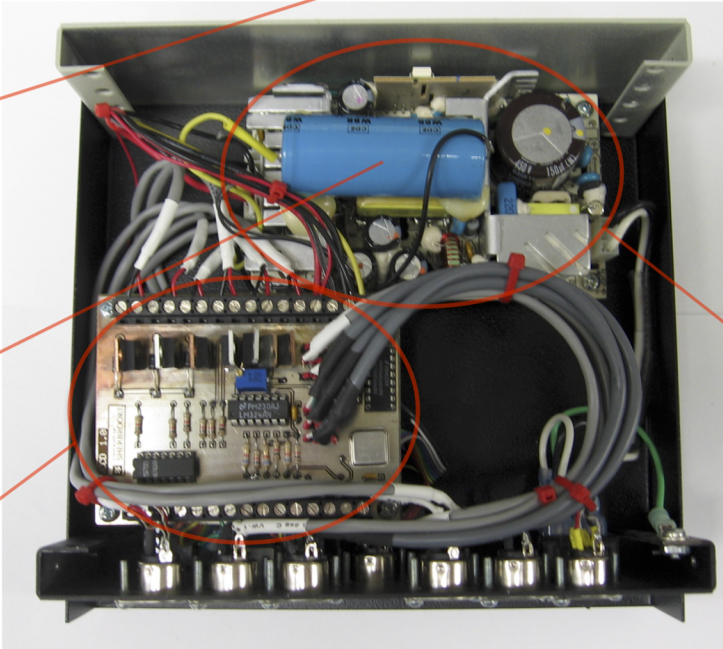

Supplement: S3 Fig — (PDF) [file pone.0133384.s004.pdf]

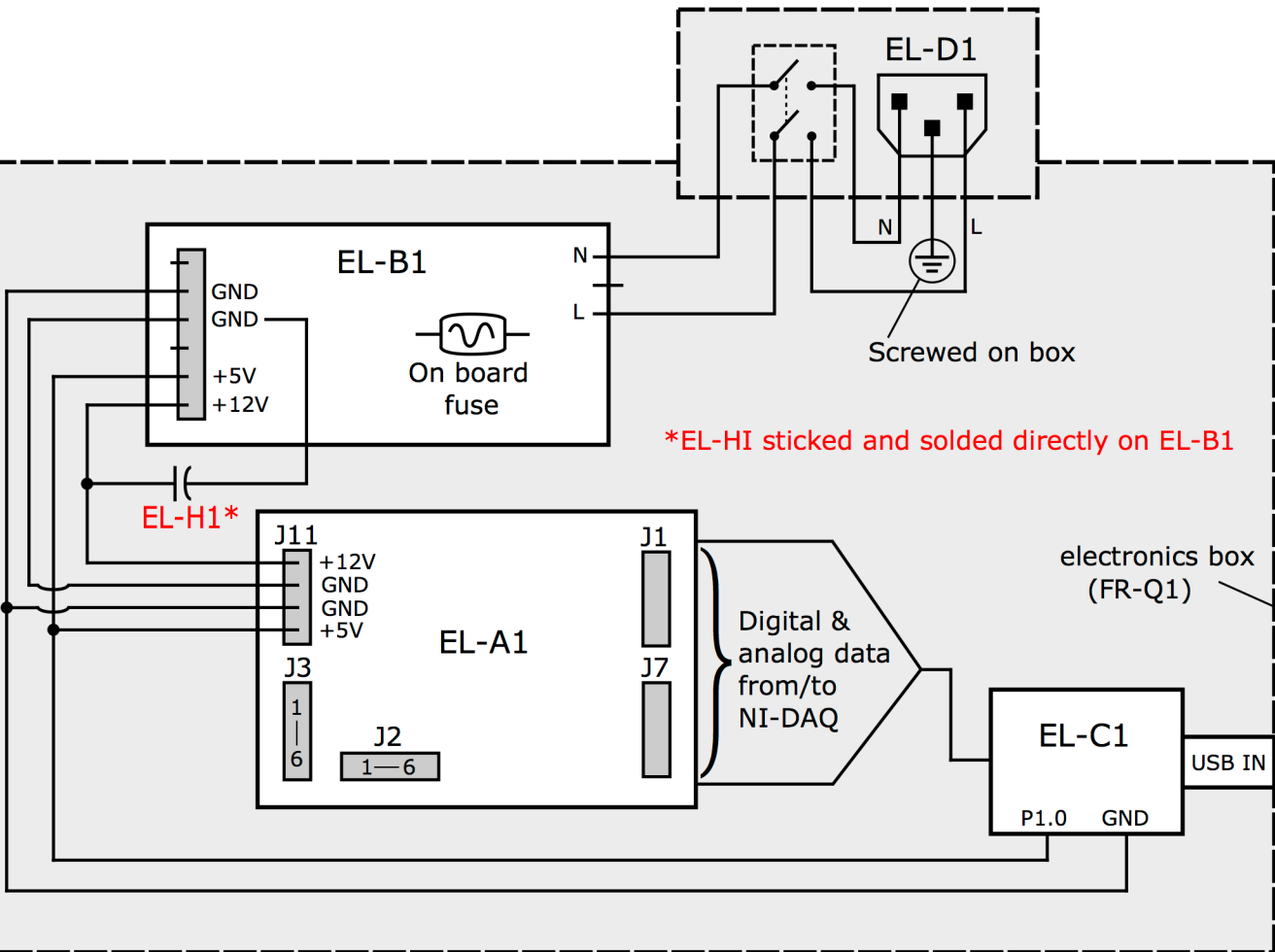

Supplement: S4 Fig — (PDF) [file pone.0133384.s005.pdf]

# Schematic diagram of the main board electronics

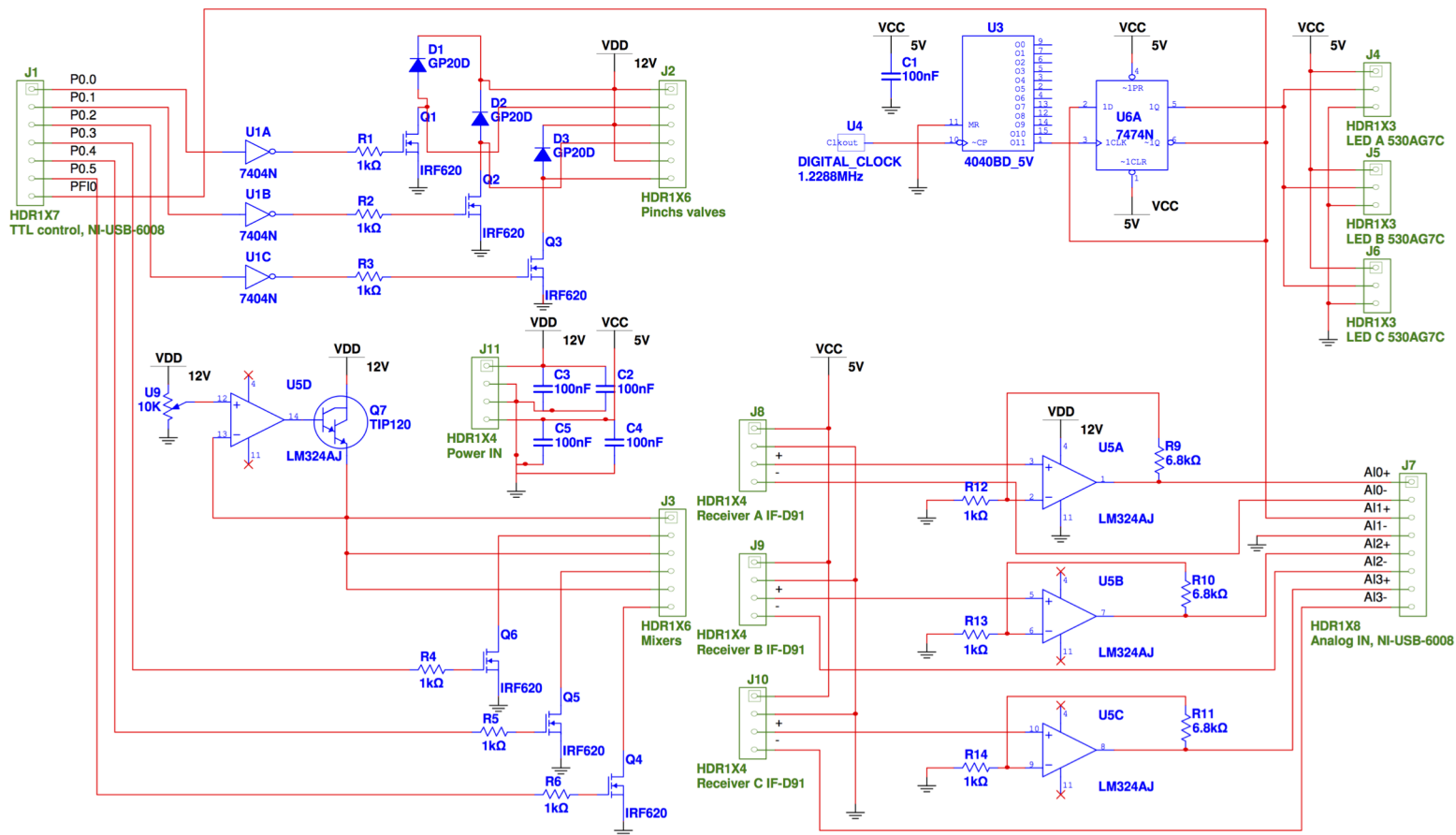

Supplement: S5 Fig — (PDF) [file pone.0133384.s006.pdf]

A

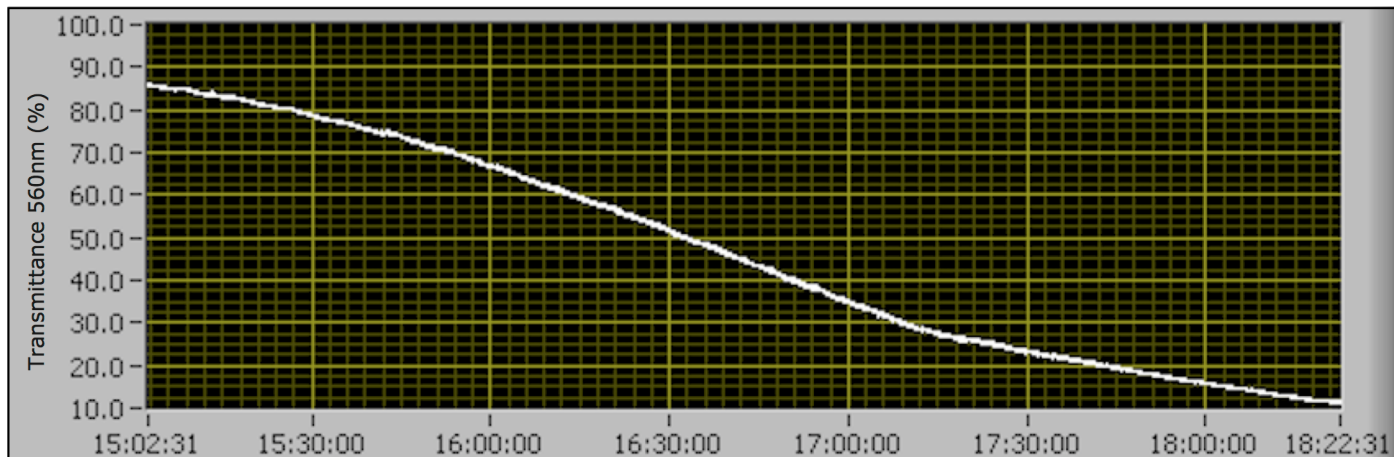

B

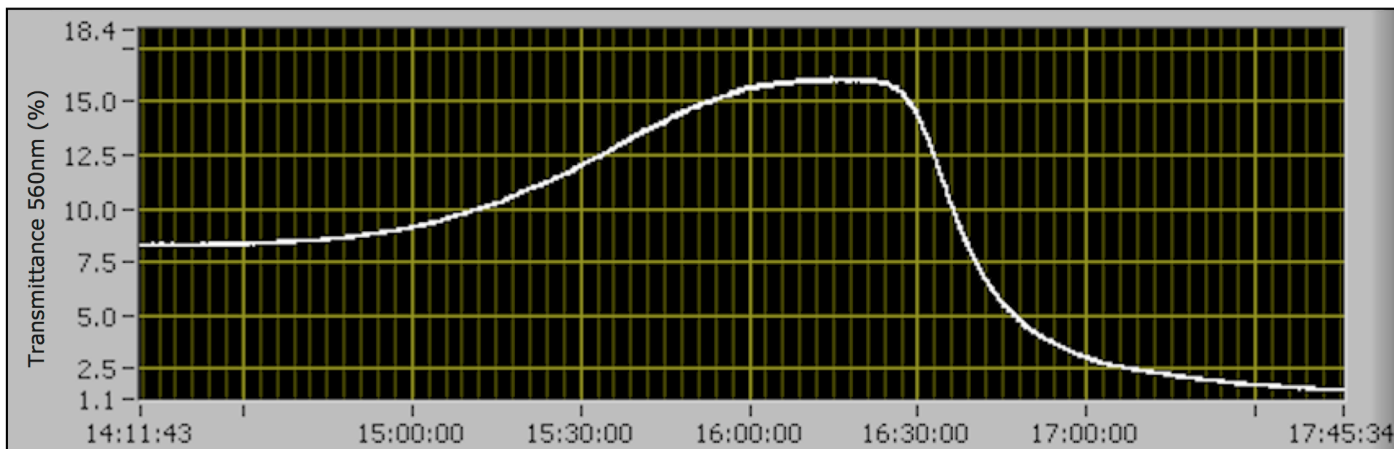

Supplement: S9 Fig — (PDF) [file pone.0133384.s010.pdf]

**A**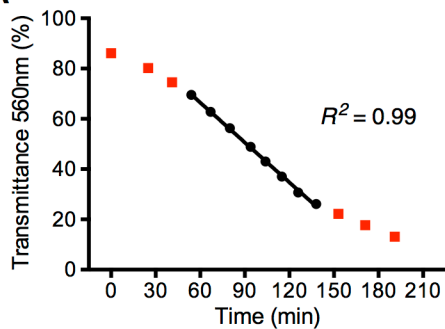**B**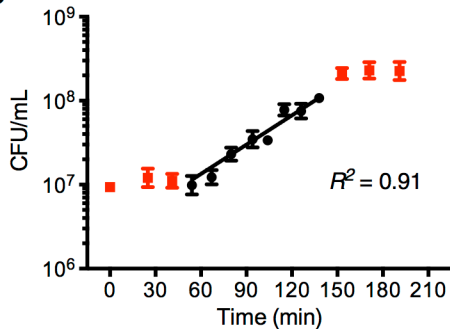**C**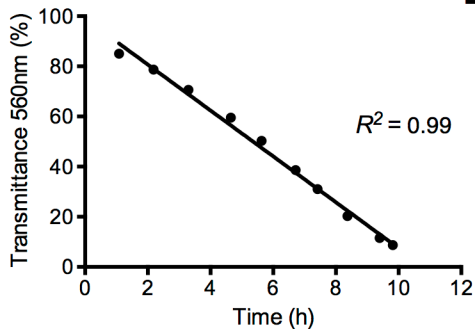**D**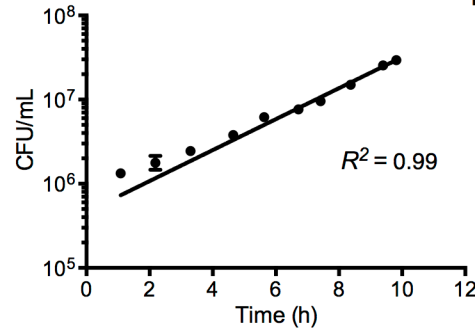**E**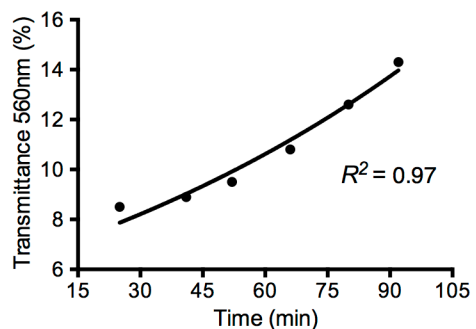**F**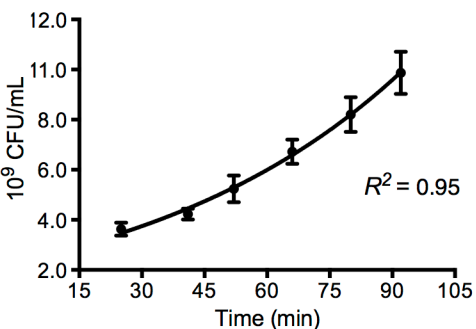

Supplement: S10 Fig — (PDF) [file pone.0133384.s011.pdf]

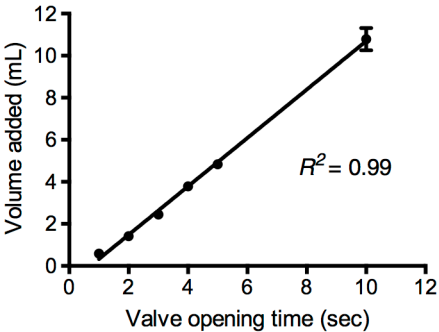

Supplement: S11 Fig — (PDF) [file pone.0133384.s012.pdf]
