# Supplementary material for: A Small-Volume, Low-Cost, and Versatile Continuous Culture Device
Source: PLoS One. 2015 Jul 21;10(7):e0133384. doi: 10.1371/journal.pone.0133384 (PMC4510131; doi:10.1371/journal.pone.0133384)
Supplement: S6 Fig — (PDF) [file pone.0133384.s007.pdf]

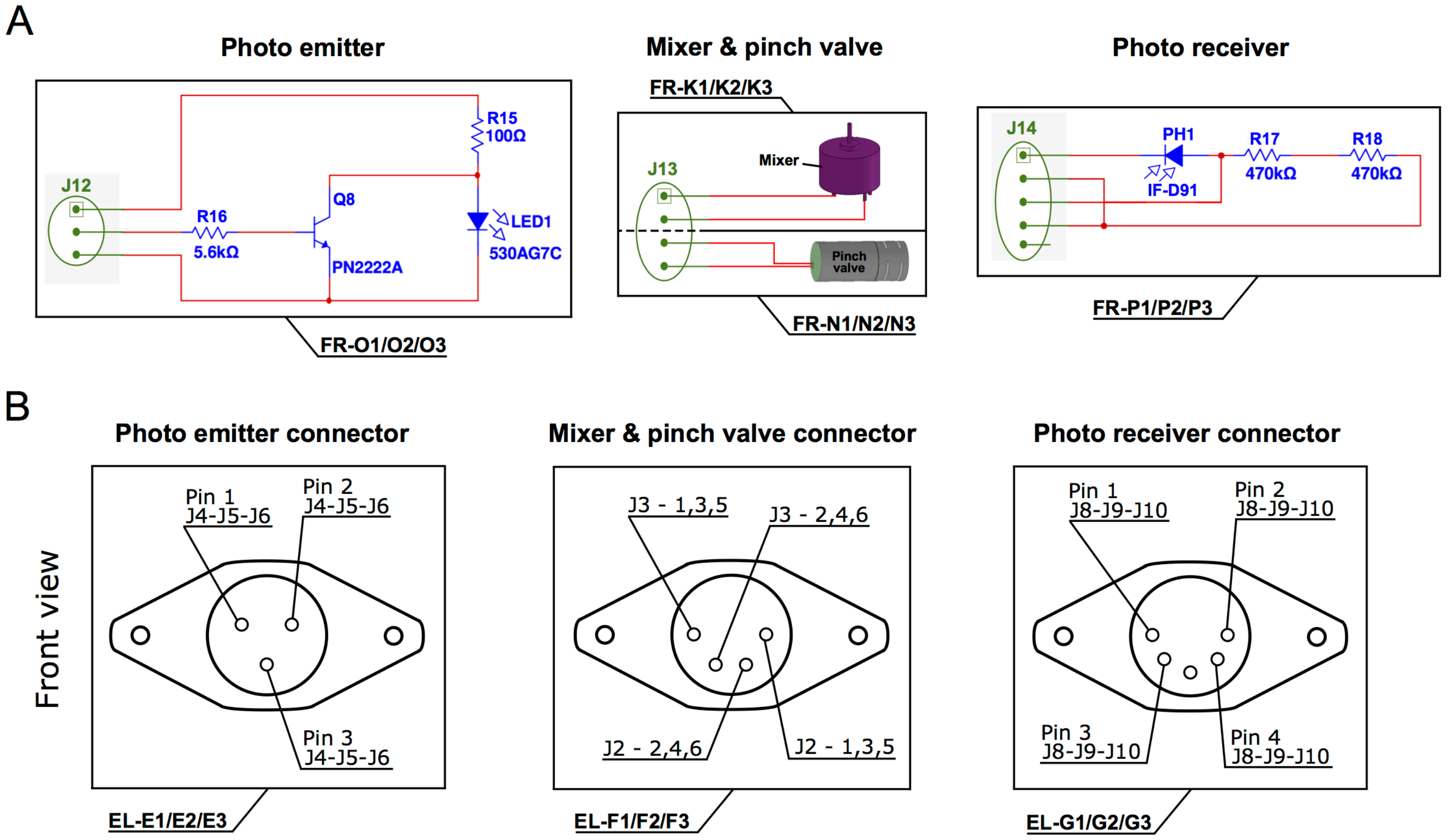
A
Photo emitter
J12
R16
5.6kΩ
Q8
PN2222A
R15
100Ω
LED1
530AG7C
FR-O1/O2/O3
Mixer & pinch valve
FR-K1/K2/K3
J13
Mixer
Pinch valve
FR-N1/N2/N3
Photo receiver
J14
PH1
IF-D91
R17
470kΩ
R18
470kΩ
FR-P1/P2/P3
B
Front view
Photo emitter connector
Pin 1 J4-J5-J6
Pin 2 J4-J5-J6
Pin 3 J4-J5-J6
EL-E1/E2/E3
Mixer & pinch valve connector
J3 - 1,3,5
J3 - 2,4,6
J2 - 2,4,6
J2 - 1,3,5
EL-F1/F2/F3
Photo receiver connector
Pin 1 J8-J9-J10
Pin 2 J8-J9-J10
Pin 3 J8-J9-J10
Pin 4 J8-J9-J10
EL-G1/G2/G3
