# Supplementary material for: A Small-Volume, Low-Cost, and Versatile Continuous Culture Device
Source: PLoS One. 2015 Jul 21;10(7):e0133384. doi: 10.1371/journal.pone.0133384 (PMC4510131; doi:10.1371/journal.pone.0133384)

Main board 3D representation

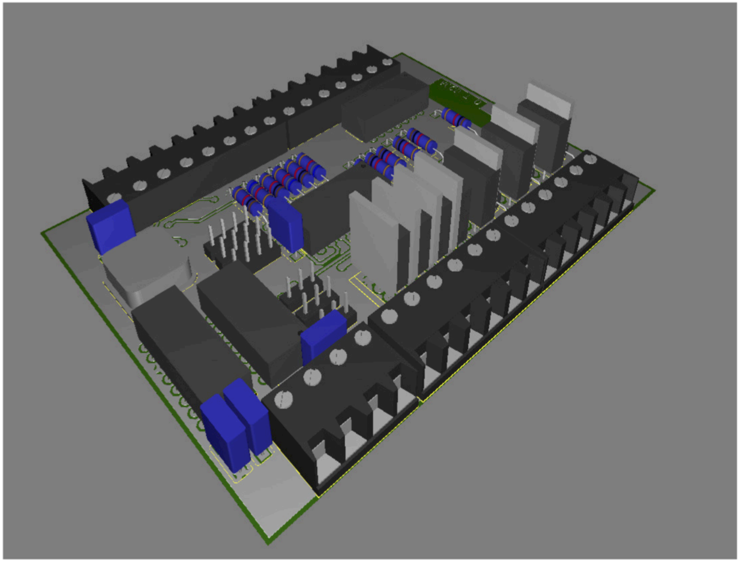

Main board silkscreen

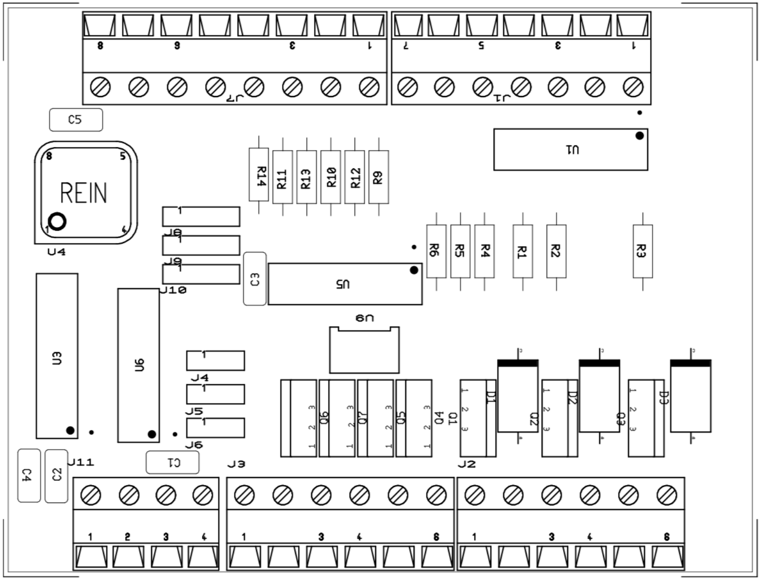

Main board solder side

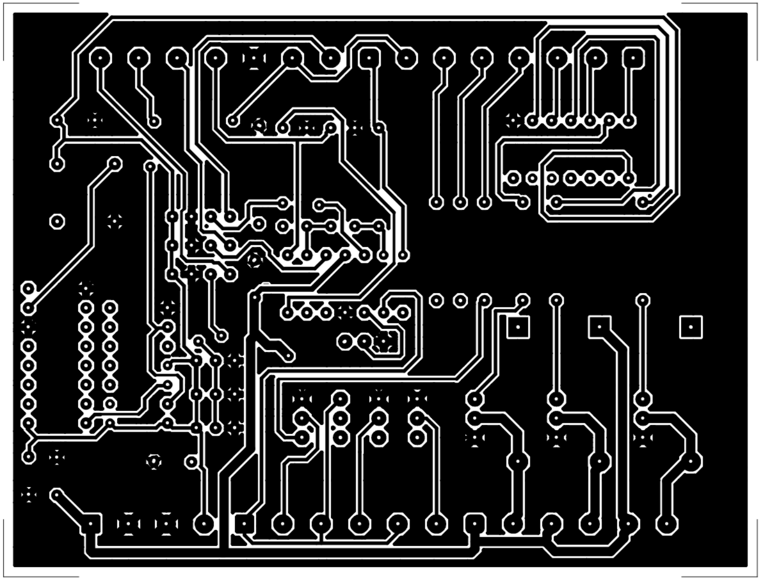

Main board component side

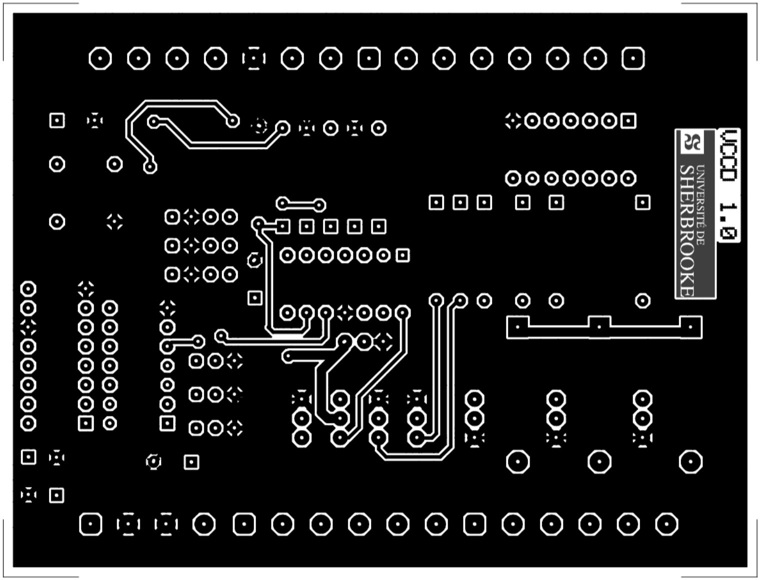

Supplement: S7 Fig — (PDF) [file pone.0133384.s008.pdf]
