# Supplementary material for: A Small-Volume, Low-Cost, and Versatile Continuous Culture Device
Source: PLoS One. 2015 Jul 21;10(7):e0133384. doi: 10.1371/journal.pone.0133384 (PMC4510131; doi:10.1371/journal.pone.0133384)

**Photo receiver silkscreen**

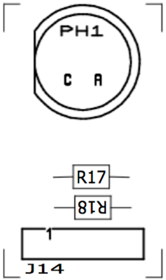


**Photo receiver solder side**

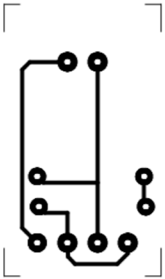

**Photo emitter silkscreen**

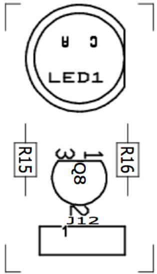


**Photo emitter solder side**

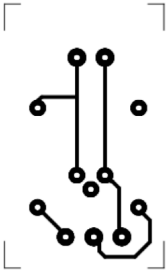

**Photo emitter component side**

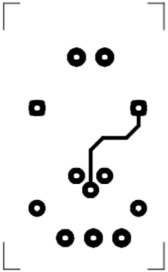

Supplement: S8 Fig — (PDF) [file pone.0133384.s009.pdf]
