# Supplementary material for: A Small-Volume, Low-Cost, and Versatile Continuous Culture Device
Source: PLoS One. 2015 Jul 21;10(7):e0133384. doi: 10.1371/journal.pone.0133384 (PMC4510131; doi:10.1371/journal.pone.0133384)
Supplement: S1 Manual — Contains all construction and assembly instructions, a system and software utilization guide, as well as important operation notes. (PDF) [file pone.0133384.s015.pdf]

Versatile Continuous Culture Device (VCCD)

User Manual - Version 1.0

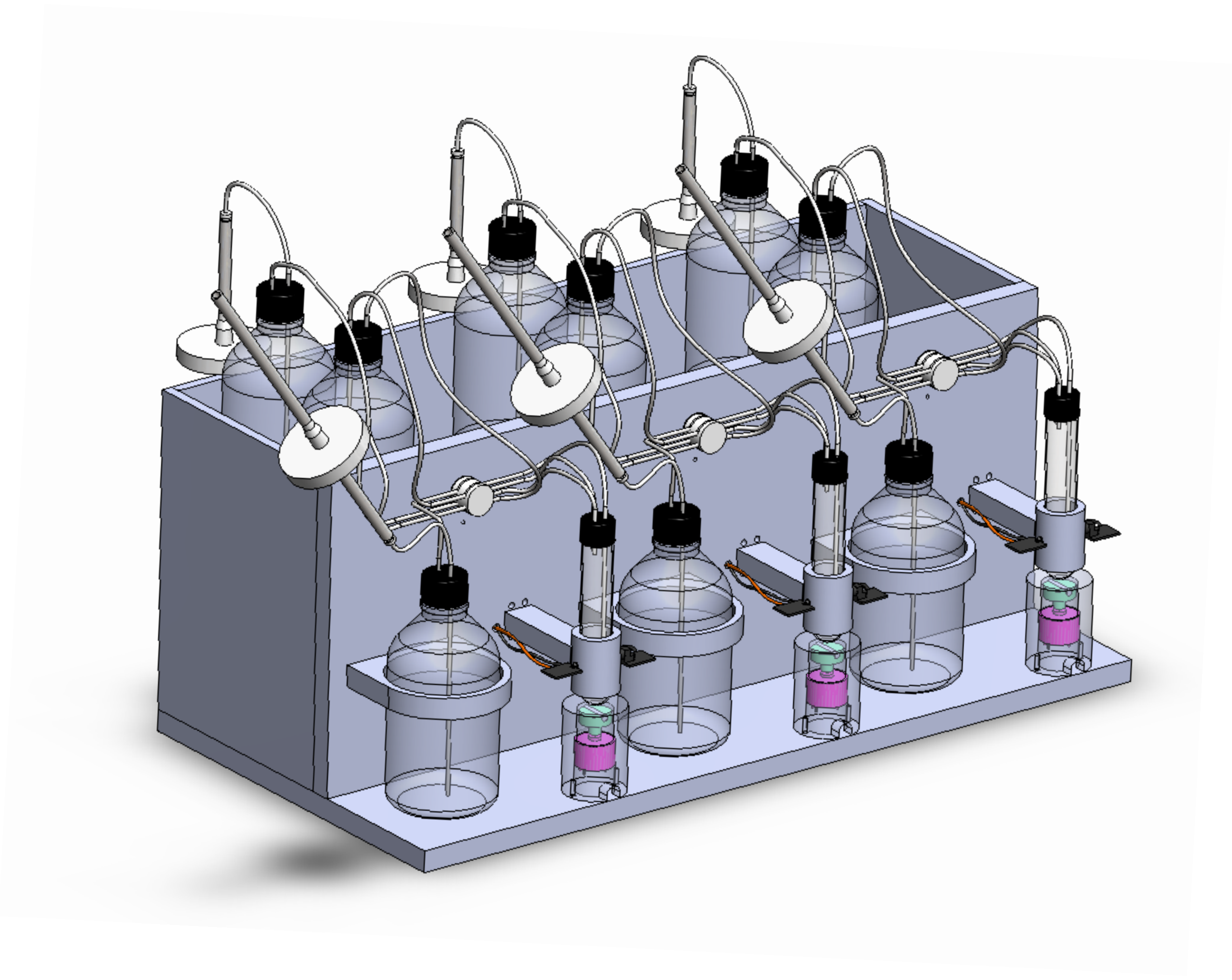

VCCD software and hardware by:

Dominick Matteau[1], Vincent Baby[1], Stéphane Pelletier[2] & Sébastien Rodrigue[1].

1- Département de biologie, Université de Sherbrooke, Sherbrooke, Québec, Canada.

2- Département de physique, Université de Sherbrooke. Sherbrooke, Québec, Canada.

Table of contents

I. Preface 4

1. Hardware description 5

1.1 Required materials 5

1.1.1 Frame material list 5

Table 1 - Frame material list (S1 Table) 5

1.1.2 Culture system material list 6

Table 2 - Culture system material list (S2 Table) 6

1.1.3 Electronics material list 7

Table 3 - Electronics material list (S3 Table) 7

1.2 VCCD assembly 10

1.2.1 Frame assembly 10

Fig. 1 - Frame assembly (S1 Fig.) 11

1.2.2 Culture system assembly 12

Fig. 2 - Culture system assembly (S2 Fig.) 13

1.2.3 Electronics assembly 14

Fig. 3 - Electronics box assembly (S3 Fig.) 15

Fig. 4 - Assembly of the main electronics components (S4 Fig.) 16

Fig. 5 - Schematic diagram of the main board electronics (S5 Fig.) 18

Fig. 6 - Schematic diagrams of the photo emitter, photo receiver, mixer, and pinch valve. A: electronics diagram; B: connectors diagram (S6 Fig.) 19

Fig. 7 - Details of the main PCB (S7 Fig.) 20

Fig. 8 - Details of the photo emitter and receiver PCBs (S8 Fig.) 20

1.3 VCCD adjustments and maintenance 21

1.3.1 Changing culture mixing speed 21

1.3.2 Adjusting pumps air flow 21

1.3.3 Adjusting height of the turbidity-measuring unit support 22

1.3.4 Washing culture system 23

1.3.5 Sterilizing the culture system 23

2. Software overview 24

2.1 Software and drivers installation 24

2.2 Launching the VCCD software 25

Fig. 9 - VCCD 1.0 graphical user interface (GUI) 26

2.3 General settings 28

2.3.1 Data acquisition rate 28

2.3.2 Calibration 29

2.3.3 Automatic print screen 30

2.3.4 Manual control of pinch valves 31
2.3.5 Expand a graph 32
2.3.6 Help 33

2.4 Creating a new experiment 35
2.4.1 Creating new data files 35
2.4.2 Data file format 36
2.4.3 Type of experiment 37
2.4.4 Culture refresh modes 38
Fig. 10 - Illustration of available continuous culture modes (A) and Time interval specific options (B) used to maintain cell growth (Fig. 4). *39*
2.4.5 Expected signal curve 39
2.4.6 *Time interval* specific modes 40
2.4.7 Show experiment configuration 41
2.4.8 Acquire button 41

3. Advanced experiment parameters 42

3.1 Advanced graphical settings 42
3.1.1 Graphic range display 42
3.1.2 Other graphical options 43
3.1.3 Curve smoothing 45

3.2 Refresh settings common to all culture refresh modes 47
3.2.1 Enable refresh 47
3.2.2 Refresh and pinch counters 47
3.2.3 Maximal number of refreshes 48
3.2.4 Stop refresh program 49
3.2.5 Pinch pause 49

3.3 Refresh settings common to multiple culture refresh modes 51
3.3.1 Minimum and maximum transmittance thresholds 51
3.3.2 Pinch time 52
3.3.3 Number of pinch cycles 52
3.3.4 Maximal pinch time and pinch cycles 53

3.4 Refresh settings specific to the *Time interval* culture refresh mode 54
3.4.1 Interval time 54
3.4.2 Interval timer initialization 54
3.4.3 *Time interval* refresh inactivation modes 56

Appendix 1 - Frame machining details (S1 Appendix) 58

Appendix 2 – Example of a typical procedure 69

I. Preface

Welcome to the versatile continuous cultivation device (VCCD) 1.0 User Manual. Consider this manual as an extension of the main publication (Matteau D et al. A small-volume, low-cost, and versatile continuous culture device) in order to build and operate your own instrument. This manual provides a thorough description of the parts and material required to build the VCCD, as well as illustrations and schematics needed to assemble the frame, culture system, and electronics of the device. It also explains all available modes and options of the VCCD 1.0 software, and contains important details and notes that would be difficult to fully explain in the main publication. VCCD 1.0 software can be downloaded at:

http://lab-rodrigue.recherche.usherbrooke.ca/VCCD_en/#Software_Download.

To avoid confusion, figures appearing in the manual that are taken from the main publication have their original title indicated in parenthesis. All figures and tables taken from the supporting information of the publication can be downloaded at:

http://lab-rodrigue.recherche.usherbrooke.ca/vccd_en/.

Since the VCCD is an open source project, we encourage any modifications or enhancements of the system, and we hope that new versions of the device will soon be developed to provide the scientific community with more possibilities of low-cost and laboratory-adapted continuous culture instruments.

1. Hardware description

The VCCD hardware is composed of three major modules: the frame, the culture system, and the electronics system. Each of these modules is described in details in the current chapter to provide all the information required to build a complete system.

1.1 Required materials

Sections 1.1.1 to 1.1.3 provide lists of all components required to build a complete VCCD. For each component listed, the required number/amount and the approximate price per instrument are given. Reference codes are indicated for each item and are used for part identification on **Figs. 1-8** and to correctly assemble the VCCD.

1.1.1 Frame material list

Table 1 provides a list of all components required to build the frame of the VCCD. It is noteworthy that almost all items described in **Table 1** need to be machined before they can be assembled together. Machining measures and details are provided in **Appendix 1**. 3D CAD files of machined frame parts (**S1 File)** are available at: http://lab-rodrigue.recherche.usherbrooke.ca/VCCD_en/#Hardware. Also note that measures of machined pieces are only given as an example and could be changed to fulfill specific needs.

Table 1 - Frame material list (S1 Table)

Product description	Source	Approx. price (USD)	Order/Part number	required number or amount per system	Approx. price per system (USD)	Reference code in Fig. 1 and Appendix 1
Clear Plexiglass acrylic sheet, 0.708'' X 24'' X 48''	eplastics.com	174.60 / Sheet	ACRYCLR0.750PM24X48	<1 (*see* **Appendix 1** for machining details)	106.42	FR-A1; B1; G1/G2/G3; H1/H2/H3
Clear Plexiglass acrylic sheet, 0.354'' X 24'' X 48''	eplastics.com	102.40 / Sheet	ACRYCLR0.375PM24X48	<1 (*see* **Appendix 1** for machining details)	44.53	FR-C1; D1/D2; E1; F1
Clear Plexiglass acrylic rod, 1.5'' Diameter, 6' Length	eplastics.com	78.34 / Rod	ACREXR1.500	<1 (*see* **Appendix 1** for machining details)	6.07	FR-I1/I2/I3
Clear Plexiglass acrylic rod, 2.0'' Diameter, 6' Length	eplastics.com	180.47 / Rod	ACREXR2.00	<1 (*see* **Appendix 1** for machining details)	20.87	FR-J1/J2/J3

Ultra Machinable Brass rod (Alloy 360), 1" Diameter, 1' Length	McMaster-CARR	36.00 / Rod	8953K981	<1 (3X 0.706'', *see* **Appendix 1** for machining details)	6.35	FR-L1/L2/L3
VWR Spinbar Magnetic Stir Bars, Polygon, 1/4 x 1" (manually decapsulated)	VWR	8.50 each	74950-288	3	25.50	FR-M1/M2/M3

1.1.2 Culture system material list

Table 2 provides a list of all components required to build the culture system of the VCCD, which is constituted of three identical continuous culture units. Tubing must be cut to the indicated length to correctly assemble the culture system depicted in **Fig. 2**, but could be adjusted if necessary.

Table 2 - Culture system material list (S3 Table)

Product description	Source	Approx. price (USD)	Order/Part number	required number or amount per system	Approx. price per system (USD)	Reference code in Fig. 2
3W 120V/60Hz aquarium pump, 1.5L/min, single output	SunSun/ Perfect	6.99 each	YT-301C	3	20.97	CS-A1
Platinum-Cured Silicone Tubing, 1/8"ID x 1/4"OD, 25 ft/pack, autoclavable	Cole-Parmer	42.00 / Pack	RK-95802-05	3X 1.5''	0.63	CS-B1
PVDF barbed Y connector, 1/16" ID, 1/32", 1/2", 3/8"; Pack of 10, autoclavable	Cole-Parmer	21.50 / Pack	EW-30703-90	3	6.45	CS-J1
Barbed fittings, Reducing Connector, Kynar, 3/16" x 1/16" ID, 1/32", 1-1/16", 3/8"; 10/pack, autoclavable	Cole-Parmer	20.00 / Pack	EW-30703-46	12	24.00	CS-C1/C2/C3/C4
PTFE Tubing, 1/16"ID x 1/8"OD, 25 ft/pack, autoclavable	Cole-Parmer	24.00 / Pack	EW-06411-62	3X (24'', 13'', 8'', 18'', 15'', 22'', 18'', 10''), 6X 7.5'', 3X 3''	35.04	CS-D1 (24''); D2 (13''); D3/D7 (7.5''); D4 (8''); D5 (18''); D6 (15'') ; D8 (22''); D9 (18''); D10 (3''); D11 (10'')
Peroxide-Cured Silicone Tubing, 1/4"ID x 3/8"OD, 25 ft/pack, autoclavable	Cole-Parmer	56.00 / Pack	RK-06411-71	9X 3'', 3X 6''	8.40	CS-E1/E2/E3 (3''); E4 (6'')
Acro 50 0.2 µm filter, hose barb with PTFE Membrane, 50 mm filter, autoclavable, pack of 18	VWR	241.67 / Pack	4251	6	80.56	CS-F1/F2
500 ml Storage/Media Bottles, KG-35 Borosilicate Glass, Screw Thread, with Black Polypropylene Closure Welded to PTFE/Silicone Liner, pack of 24	Kimble Chase	208.12 / Pack	61110P-500	9	78.05	CS-H1/H2/H3

PYREX 55mL Screw Cap Culture Tubes with Phenolic Caps, 25x150mm, pack of 48	Corning	430.30 / Pack	9825-25	3	26.89	CS-L1
PTFE Tubing, 1/16"ID x 1/8"OD, 25 ft/pack, autoclavable	Cole-Parmer	40.00 / Pack	OF-06605-27	24X 2'', 3X 4.25''	8.10	CS-I1/I2/I3/I4/I5/I6/I7/I8 (2''); I9 (4.25'')
Fluorocarbon O-Rings, 75 Duro, 11/16'' I.D, 3/32 width, -20° to +400°F, autoclavable	Hercules Sealing Products	0.74 each	568-115V	3	2.22	CS-K1
Fluorocarbon O-Rings, 75 Duro, 1'' I.D, 3/32 width, -20° to +400°F, autoclavable	Hercules Sealing Products	0.98 each	568-120V	9	8.82	CS-G1/G2/G3
VWR Spinbar Stir Bars, Octagon, 5/16 x 1/2", pack of 6	VWR	49.85 / Pack	58949-036	3	24.93	CS-M1

1.1.3 Electronics material list

Table 3 provides a list of all components required to build the electronics controlling the VCCD. Note that the computer connected to the USB port of the NI-DAQ USB-6008 needed to execute the VCCD software is not included in this list.

Table 3 - Electronics material list (S3 Table)

Product description	Source	Approx. price (USD)	Order/Part number	required number or amount per system	Approx. price per system (USD)	Reference code in Figs. 1-8
CONNECTORS, HDR1X6	Digi-Key	3.89	796949-6-ND	3	11.67	J1 (digital output from EL-C1 [NI-USB-6008]), J2 and J3 (connected to FR-N1/N2/N3 [pinch valves] and to FR-K1/K2/K3 [mixers] via EL-F1/F2/F3)
MOS_3TEN, IRF620	Digi-Key	0.96	IRF620PBF-ND	6	5.76	Q1/Q2/Q3/Q4/Q5/Q6
NPN Transistor, TIP120	Digi-Key	0.66	TIP120-ND	1	0.66	Q7
74STD, 7474N	Digi-Key	0.62	296-1668-5-ND	1	0.62	U6
74HC, 74HC4040N	Digi-Key	0.71	296-8324-5-ND	1	0.71	U3
Variable resistor, 10K	Digi-Key	2.38	3299W-103LF-ND	1	2.38	U9
CLOCK OSCILLATOR 1.2288MHz	Digi-Key	2.98	XC230-ND	1	2.98	U4
CONNECTORS, HDR1X3(strip)	Digi-Key	0.95	609-2224-ND	3	2.85	J4/J5/J6 (connected to FR-O1/O2/O3 [Photo emitters] via EL-E1/E2/E3)
CONNECTORS, HDR1X8	Digi-Key	5.42	796949-8-ND	1	5.42	J7 (analog input to EL-C1 [NI-USB-6008])
CONNECTORS, HDR1X4(strip)	Digi-Key	0.95	609-2224-ND	3	2.85	J8/J9/J10 (connected to FR-P1/P2/P3 [Photo receivers] via EL-G1/G2/G3)

RESISTOR, 1kΩ	Digi-Key	0.08	CF14JT1K00CT-ND	9	0.72	R1/R2/R3/R4/R5/R6 and R12/R13/R14
RESISTOR, 6.8kΩ	Digi-Key	0.08	CF14JT6K80CT-ND	3	0.24	R9/R10/R11
CONNECTORS, HDR1X4	Digi-Key	2.30	796949-4-ND	1	2.3	J11
CAPACITOR, 100nF	Digi-Key	0.29	445-5303-ND	5	1.45	C1/C2/C3/C4/C5
DIODE, GP20D	Digi-Key	0.51	EGP20DFSCT-ND	3	1.53	D1/D2/D3
OPAMP, LM324N	Digi-Key	0.39	296-1391-5-ND	1	0.39	U5
74STD, 74LS04N	Digi-Key	0.74	296-1629-5-ND	1	0.74	U1
CONN DIN 3 PIN FEMALE PANEL MNT	Digi-Key	1.75	CP-1230-ND	3	5.25	EL-E1/E2/E3 (connected to J4/J5/J6; connected to J12)
CONN DIN 4 PIN FEMALE PANEL MNT	Digi-Key	1.84	CP-1240-ND	3	5.52	EL-F1/F2/F3 (connected to J2 and J3; connected to J13)
CONN DIN 5 PIN FEMALE PANEL MNT	Digi-Key	2.74	CP-1250-ND	3	8.22	EL-G1/G2/G3 (connected to J8/J9/J10; connected to J14)
CONN CIRCULAR DIN 3 PIN MALE	Digi-Key	1.66	CP-1030-ND	3	4.98	J12 (connected to EL-E1/E2/E3)
CONN CIRCULAR DIN 4 PIN MALE	Digi-Key	1.71	CP-1040-ND	3	5.13	J13 (connected to EL-F1/F2/F3)
CONN CIRCULAR DIN 5 PIN MALE	Digi-Key	1.79	CP-1050-ND	3	5.37	J14 (connected to EL-G1/G2/G3)
PCB COPPER CLAD POS 6X9" 2-SIDE	Digi-Key	21.35	473-1024-ND	1	21.35	EL-A1 (main board, ~3X4''), photo emitters (FR-O1/O2/O3, ~0.65X1'') and photo receivers (FR-P1/P2/P3, ~0.65X1'') printed circuit boards
BOX STEEL 8X8X3" BLACK	Digi-Key	40.51	HM338-ND	1	40.51	FR-Q1 (electronics box)
MODULE POWER ENTRY FLANGE 10A	Digi-Key	7.44	CCM1666-ND	1	7.44	EL-D1 (connected to EL-J1)
Power Supply +12V/-12V/+5V	Digi-Key	63.01	454-1314-ND	1	63.01	EL-B1
NPN Transistor PN2222A	Digi-Key	0.22	PN2222ATFCT-ND	3	0.66	Q8
RESISTOR, 5.6kΩ	Digi-Key	0.08	CF14JT5K60CT-ND	3	0.24	R16
RESISTOR, 100Ω	Digi-Key	0.08	CF14JT100RCT-ND	3	0.24	R15
RESISTOR, 470kΩ	Digi-Key	0.08	CF14JT470KCT-ND	6	0.48	R17 and R18
DAQ USB-6008	National Instruments	189.00	NI USB-6008	1	189,00	EL-C1 (connected to J1 and J7, and to a computer USB port by EL-I1)
CAP ALUM 3000UF 35V AXIAL	Digi-Key	19.32	WBR3000-35A-ND	1	19.32	EL-H1 (sticked and solded directly on EL-B1)
6 VDC, 2100 RPM DC MOTOR	Jameco electronics	3.74 each	2173044	3	11.22	Mixer (connected to J3 via J13 and EL-F1/F2/F3), part of FR-K1/K2/K3
5mm Green LED 300mcd/30° @20mA - Water Clear Lens, 573nm	Electron.co m	0.04 each	530AG7C	3	0.12	LED1 (connected to J4/J5/J6 via J12 and EL-E1/E2/E3), part of FR-O1/O2/O3

Photodiode Receiver, Fiber Optics, Plastic, 60nA, 100Mbps	Digi-Key	4.18 each	FB120-ND	3	12.54	PH1 (connected to J8/J9/J10 via J14 and EL-G1/G2/G3), part of FR-P1/P2/P3
Solenoid operated 3-way pinch valve for dual silicone tubing, 12V	BIO-CHEM FLUIDICS	130.77 each	100PD3MP12-02S	3	392.31	Pinch valve (connected to J2 via J13 and EL-F1/F2/F3), part of FR-N1/N2/N3
USB cable A-B male 3M 2.0	Digi-Key	3.30	AE1463-ND	1	3.30	EL-I1
Power cord 6 feet AWG 18/3 SVT Black	Jameco electronics	4.49	161761	1	4.49	EL-J1

1.2 VCCD assembly

Sections 1.2.1 to 1.2.3 provide the information needed to assemble the VCCD from parts and components listed in **Tables 1-3**. Every part displayed on assembly figures is identified with the reference code given in **Tables 1-3**. Most frame parts (*see* **Table 1**) must be machined before being assembled (*see* **Appendix 1**). An example of the assembled VCCD is available in 3D CAD file format at http://lab-rodrigue.recherche.usherbrooke.ca/VCCD_en/#Hardware (S2 **File**).

1.2.1 Frame assembly

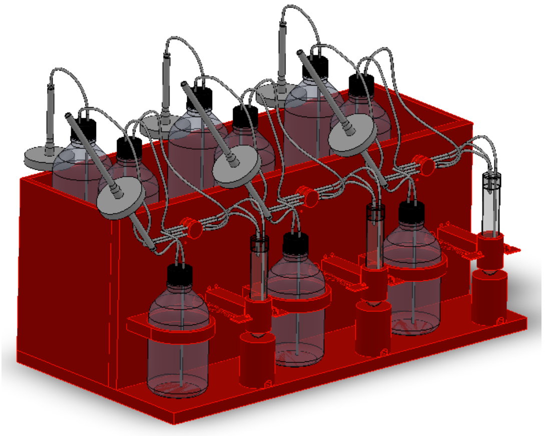

Fig. 1 shows how the components listed in **Table 1**, and some listed in **Table 3**, are assembled together to build the complete frame of the VCCD system. Note that screws and glue used to assemble the different components are not represented on the figure and they are left to the discretion of the user. To keep the schematic clear and concise, wires of pinch valves and electric motors are also not displayed. For the same reason, measures of the machined pieces are not represented here and are available in **Appendix 1**. The VCCD frame is mainly used to support the culture system and to ensure the proper operation of electronics components. Briefly, the front plexiglass panel (FR-B1) supports three pinch valves (FR-N1/N2/N3) used to control culture refreshes by pinching silicone tubing (CS-D5; D6; D8; D9 in **Fig. 2**). This panel also supports three identical plexiglass montages, each of them constituted of two plexiglass pieces (FR-H1/H2/H3; I1/I2/I3) that hold a photo emitter (FR-O1/O3/O3) and a photo receiver (FR-P1/P2/P3). Culture vessels (CS-L1 in **Fig. 2**) are held by three identical mixing motor assemblies that are fixed on the frame base (FR-A1). Because these assemblies are identical, only one is illustrated in exploded view on **Fig. 1**. Each of them comprises a 6 volts rotating motor (FR-K1/K2/K3) mounted by a machined brass piece (FR-L1/L2/L3), both located inside a machined plexiglass rod (FR-J1/J2/J3). To mix cultures, the rotating brass piece supports a fixed decapsulated magnetic stir bar (FR-M1/M2/M3) used to rotate another magnetic stir bar (CS-M1 in **Fig. 2**) located inside the culture vessel. The mixing speed can be adjusted according to experimental needs (*see* **section 1.3.1**). The frame base (FR-A1) also supports an electronics box (FR-Q1) as well as three air pumps (CS-A1 in

Fig. 1 - Frame assembly (S1 Fig.)

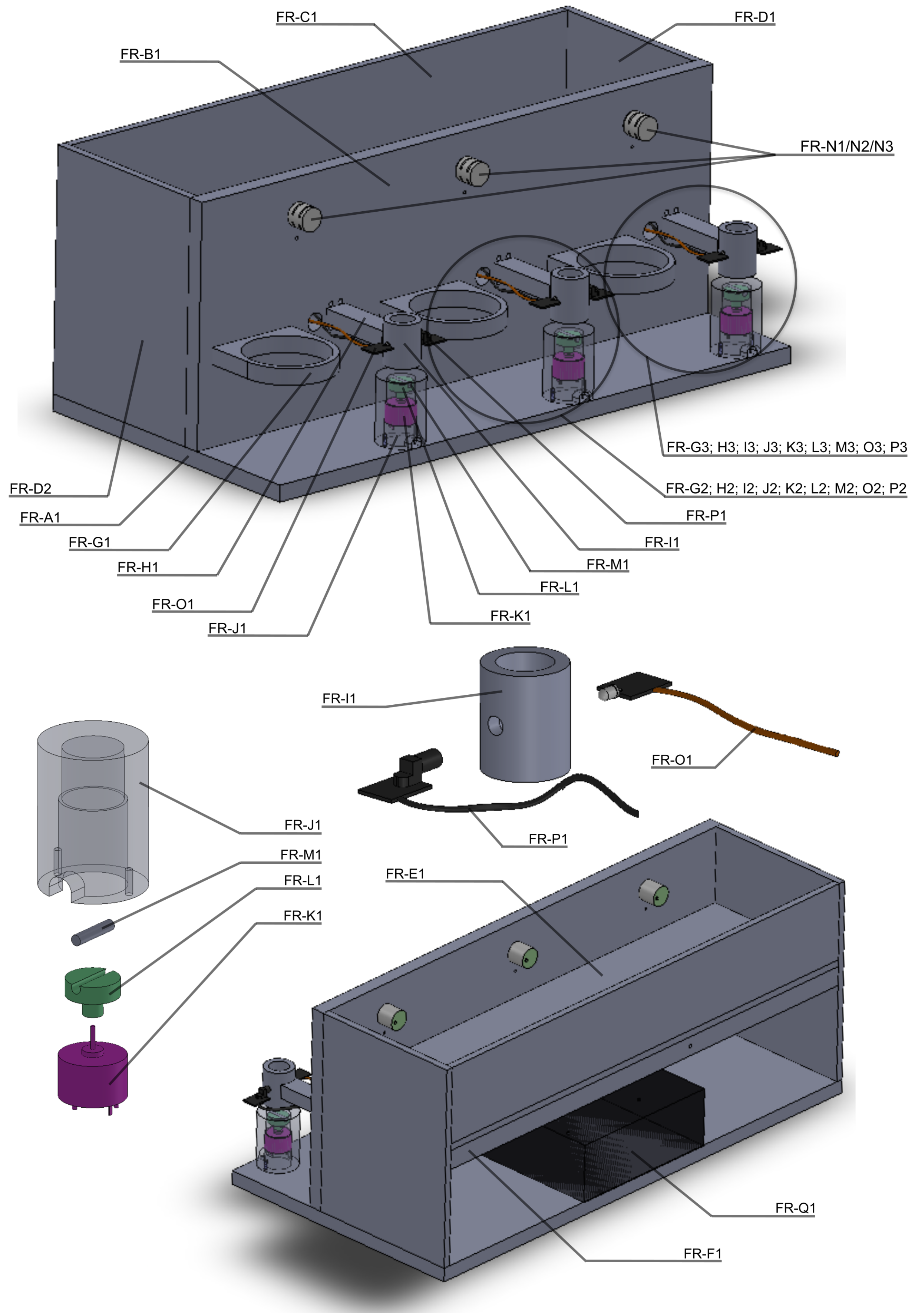

Fig. 2) required to perform culture refreshes. The plexiglass piece FR-G1 holds a trash bottle (CS-H3 in **Fig. 2**), while the plexiglass piece FR-E1 supports two other bottles that are part of the culture system (CS-H1/H2 in **Fig. 2**) that respectively contain water and fresh medium.

1.2.2 Culture system assembly

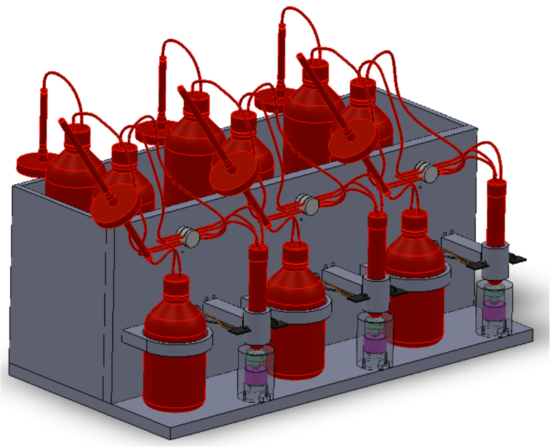

Fig. 2 shows how the components listed in **Table 2** are assembled together to build the culture system of the VCCD. Because the culture system is constituted of three identical continuous culture units, only one is displayed in **Fig. 2**. Silicone sealant used to seal tubing on fittings, as well as epoxy adhesive used to seal polytetrafluoroethylene (PTFE) tubing on bottle caps are not represented on the figure. To keep the schematic clear, tubing measures suggested in **Table 2** are also not displayed. Briefly, an aquarium air pump (CS-A1) is connected to a bottle containing water (CS-H1) through flexible silicone tubing (CS-B1; D1; D2; E1; E2), rigid fittings (CS-C1/C2/C3), and a 0.2 µm filter (CS-F1) to avoid medium contamination. This air pump provides an adjustable air flow (*see* **section 1.3.2**) inside the culture system that enables liquid displacement and culture dilution. Connection of the CS-D2 silicone tube to the water bottle (CS-H1) is provided by a rigid PTFE tube (CS-I1) that passes through a pierced bottle cap containing a sealing fluorocarbon O-ring (CS-G1). This tube (CS-I1) is then connected to a silicone tube (CS-D3) that extends to the bottom of the water bottle to ensure air humidification. Humidified air then passes through another PTFE tube (CS-I2) also inserted into the pierced bottle cap of CS-H1. Because the other bottle cap assemblies part of the culture system (medium and trash bottles) are identical to the water bottle cap assembly, only one is depicted in **Fig. 2**. Another silicone tube (CS-D4) links the CS-I2 tube to a rigid Y connector (CS-J1) that splits the air flow in the system. The Y connector is connected to two other silicone tubes: CS-D5 that is connected to a bottle containing fresh medium (CS-H2), and CS-D6 that is connected to a culture vessel (CS-L1) containing a magnetic stirring bar (CS-M1). These connections are provided by two tubes (CS-I3 and I7) that are part of the culture vessel and medium bottle cap assemblies, respectively. To be able to deliver fresh medium into the culture vessel, a silicone tube (CS-D7) extends from the bottom of medium bottle (CS-H2) to a second PTFE tube (CS-I4) that

Fig. 2 - Culture system assembly (S2 Fig.)

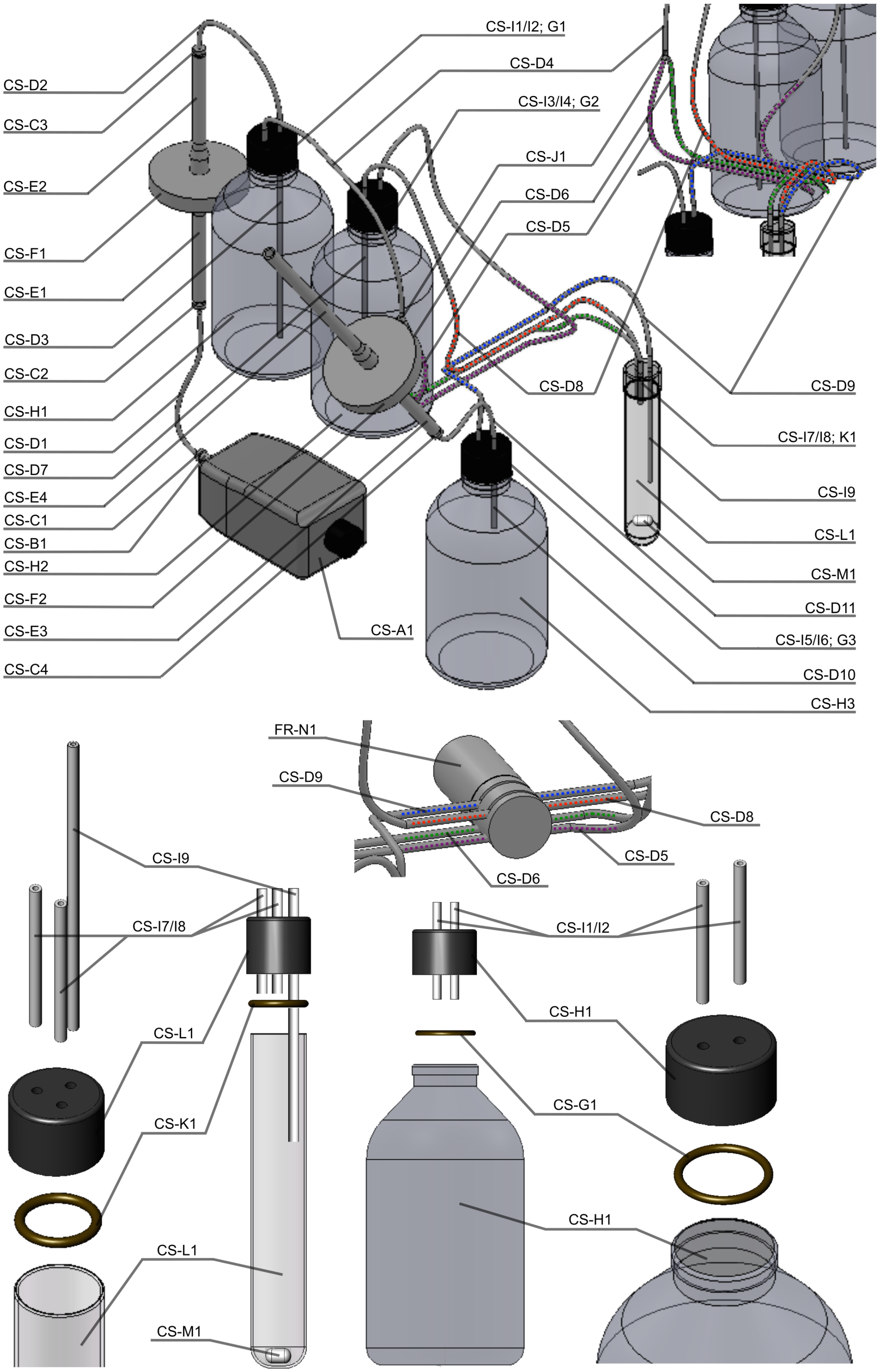

passes through the medium bottle cap. The CS-I4 tube is next connected to the culture vessel (CS-L1) by a silicone tube (CS-D8) and a second PTFE tube (CS-I8) inserted into the culture vessel cap. A third PTFE tube (CS-I9) also passes through the culture vessel cap to evacuate the exceeding volume of culture when culture refresh is performed. With its proposed length (4.25"), this tube restrains the volume of the culture vessel to 20 mL, but could easily be adjusted to support continuous cultures of different volumes. This tube is next connected to a trash bottle (CS-H3) via a silicone tube (CS-D9) and a PTFE tube (CS-I5) part of another bottle cap assembly. The other PTFE tube (CS-I6) of the trash bottle is finally connected to silicone tubing (CS-D10; D11; E3; E4), a rigid fitting (CS-C4), and a second 0.2 µm filter (CS-F2), which allows the evacuation of the air circulating through the culture system. To obtain a functional continuous culture system, the depicted silicone tubing CS-D5, D6, D8, and D9 must pass through a four-way pinch valve (FR-N1) as depicted in **Fig. 2**. In the normal state (no refresh), the pinch valve pinches CS-D5 and CS-D8 tubing, so that the air flow is evacuated through CS-D6 and CS-D9 and no pressure is present inside the culture tube. When a refresh cycle begins, the pinch valve changes position and now pinches CS-D6 and CS-D9 tubing, which generates a low pressure inside the culture system. This pressure causes a liquid displacement from the medium bottle (CS-H2) to the culture vessel (CS-L1) and therefore a culture dilution. After a refresh, the pinch valve returns to its original position, and the excess of culture volume is aspirated into the trash bottle (CS-H3). All bottle caps must contain fluorocarbon O-rings and have to be screwed tightly on bottles to ensure culture system sealing. Although the system must be sealed to generate enough pressure required to displace liquid during a culture refresh event, the culture vessel cap should not be screwed too tightly on the culture vessel (CS-L1) in order to evacuate the excess of pressure. If the culture vessel is completely sealed, the amount of liquid added during a refresh will not be proportional to the pinch valve activation time. Also, this excess of pressure could damage the air pump if maintained over long periods.

1.2.3 Electronics assembly

Figs. 3-8 illustrate how to assemble to components listed in **Table 3** to build the electronics system controlling the VCCD. **Fig. 3** is an overview of the main electronics components included in the electronics box (FR-Q1). **Fig. 4** illustrates how these components are connected with each other. **Fig. 5** is a schematic diagram of the main printed circuit board (PCB) (EL-A1). **Fig. 6**

shows schematic diagrams of the photo emitter and photo receiver PCBs (EL-O1/O2/O3, EL-P1/P2/P3, respectively), as well as diagrams of the pinch valves and mixers electrical connections (FR-N1/N2/N3, FR-K1/K2/K3, respectively). Because the VCCD system comprises three identical photo emitter PCBs, photo receiver PCBs, pinch valve and mixer systems, only one of each is depicted in **Fig. 6**. This figure also illustrates how these components are connected to the main PCB. **Figs. 7 and 8** illustrate the details needed for PCBs fabrication. Electronics schematic diagrams (**Figs. 5 and 6**) and PCB layouts (**Figs. 7 and 8**) were designed using the National Instruments Circuit Design Suite 11.0 software. PCBs were created using double-sided presensitized copper clad boards and a Kinsten KVB-30 UV exposure box according to the manufacturer's specifications. Note that screws needed to fix the different electronics components inside the electronics box (FR-Q1), as well as electrical wiring connecting the different electronics

Fig. 3 - Electronics box assembly (S3 Fig.)

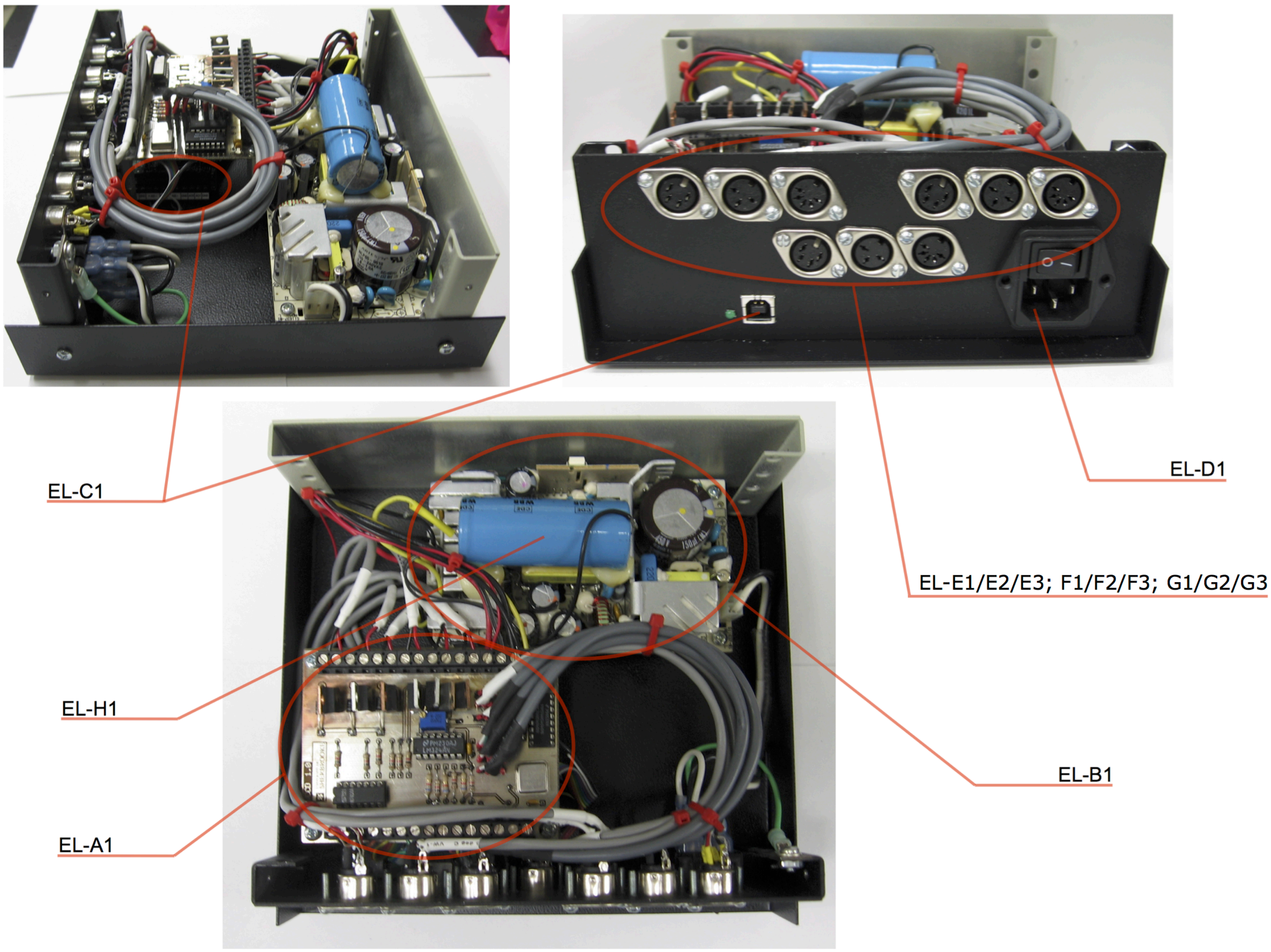

Fig. 4 - Assembly of the main electronics components (S4 Fig.)

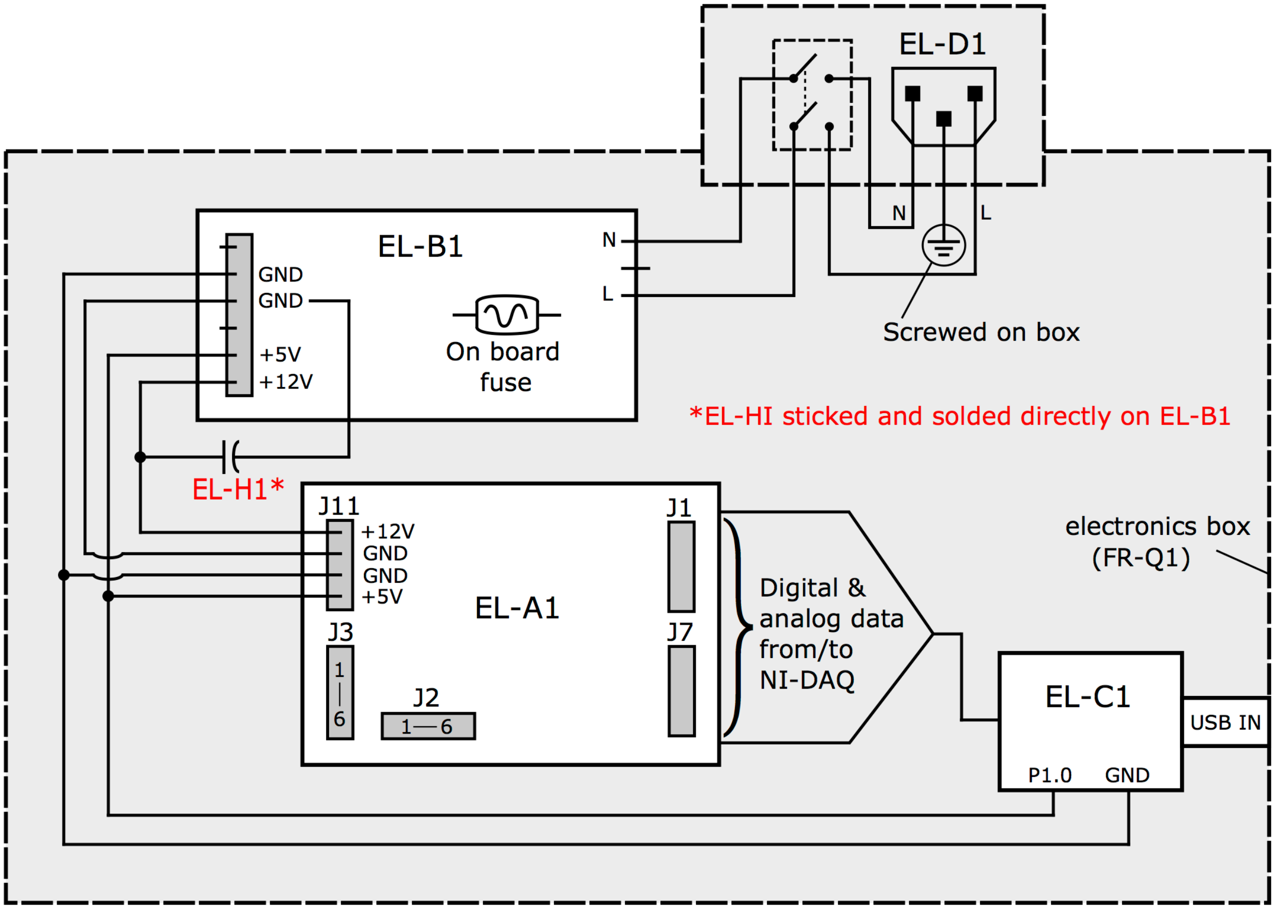

parts are not listed in **Table 3** and are left to the discretion of the user. Furthermore, soldering paste required to fix electronics parts on PCBs is not illustrated on **Figs. 3-8**. This step was done by hand using a Weller EC1002 soldering iron and Kester 44 Rosin Sn63Pb37 0.031'' diameter solder wire (Digi-Key KE1102-ND).

The VCCD electronics are composed of five major parts: the 5V/12V power supply (EL-B1), the main PCB (EL-A1), the NI-DAQ USB-6008 (EL-C1), the photo emitter PCB (FR-O1/O2/O3), and the photo receiver PCB (FR-P1/P2/P3). The power supply is connected to a 10A power entry connector (EL-D1) equipped with an on/off switch, which is then connected to a regular 120V power outlet by a standard 125V computer power cord (EL-J1). The power supply provides 5V and 12V power to the main PCB. The main PCB then supplies 5V power to the LEDs (LED1) of the photo emitter PCBs, as well as to the photodiode receivers (PH1) of the photo emitters PCBs, whereas it supplies 12V power to the pinch valves (FR-N1/N2/N3) and the mixing motors (FR-K1/K2/K3). Photo emitters, pinch valves and mixers, and photo receivers are connected through

three different circular connectors to the main PCB to avoid any chance of misconnection (EL-E1/E2/E3, F1/F2/F3, G1/G2/G3, respectively). The LEDs pulse frequency (150 Hz) is provided by a 1.2288 MHz clock oscillator (U4) mounted on the main PCB. The photo receivers are also synchronized to the same frequency to eliminate possible noise from ambient light. Change of light detected by the photo receivers, i.e. change of transmittance caused by bacterial growth, is converted to a change of voltage in the circuit. The change of voltage is independently monitored by the NI-DAQ USB-6008 card (EL-C1) for each transmittance-measuring unit, and transmitted to a computer by a USB cable (EL-I1). Acquired voltage data of each channel, as well as the corresponding transmittance values are displayed on the computer using the VCCD software. The software is also used to activate pinch valves in specific conditions (*see* **chapters 2 and 3** for more details).

Fig. 5 - Schematic diagram of the main board electronics (S5 Fig.)

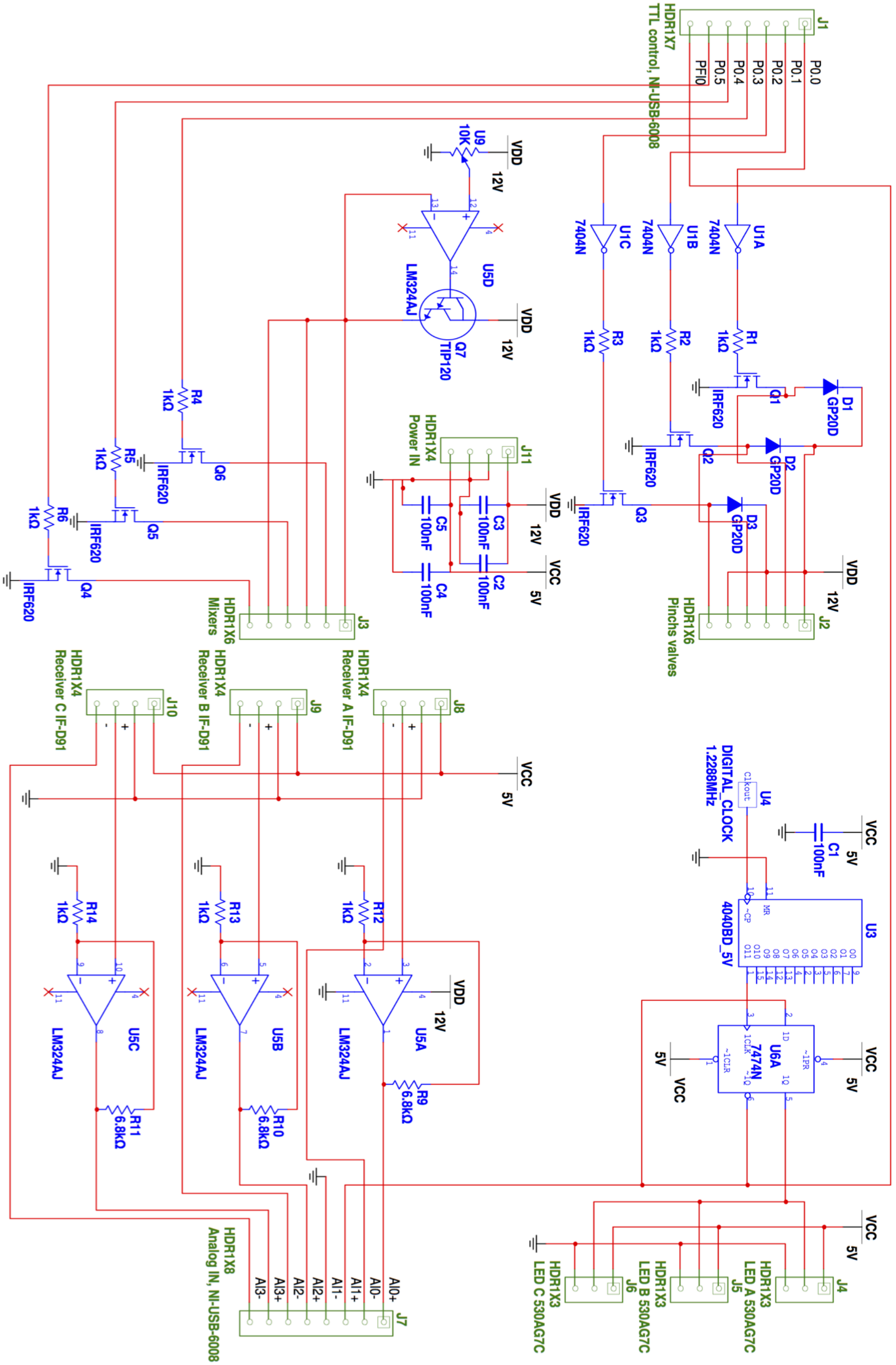

Fig. 6 - Schematic diagrams of the photo emitter, photo receiver, mixer, and pinch valve. A: electronics diagram; B: connectors diagram (S6 Fig.)

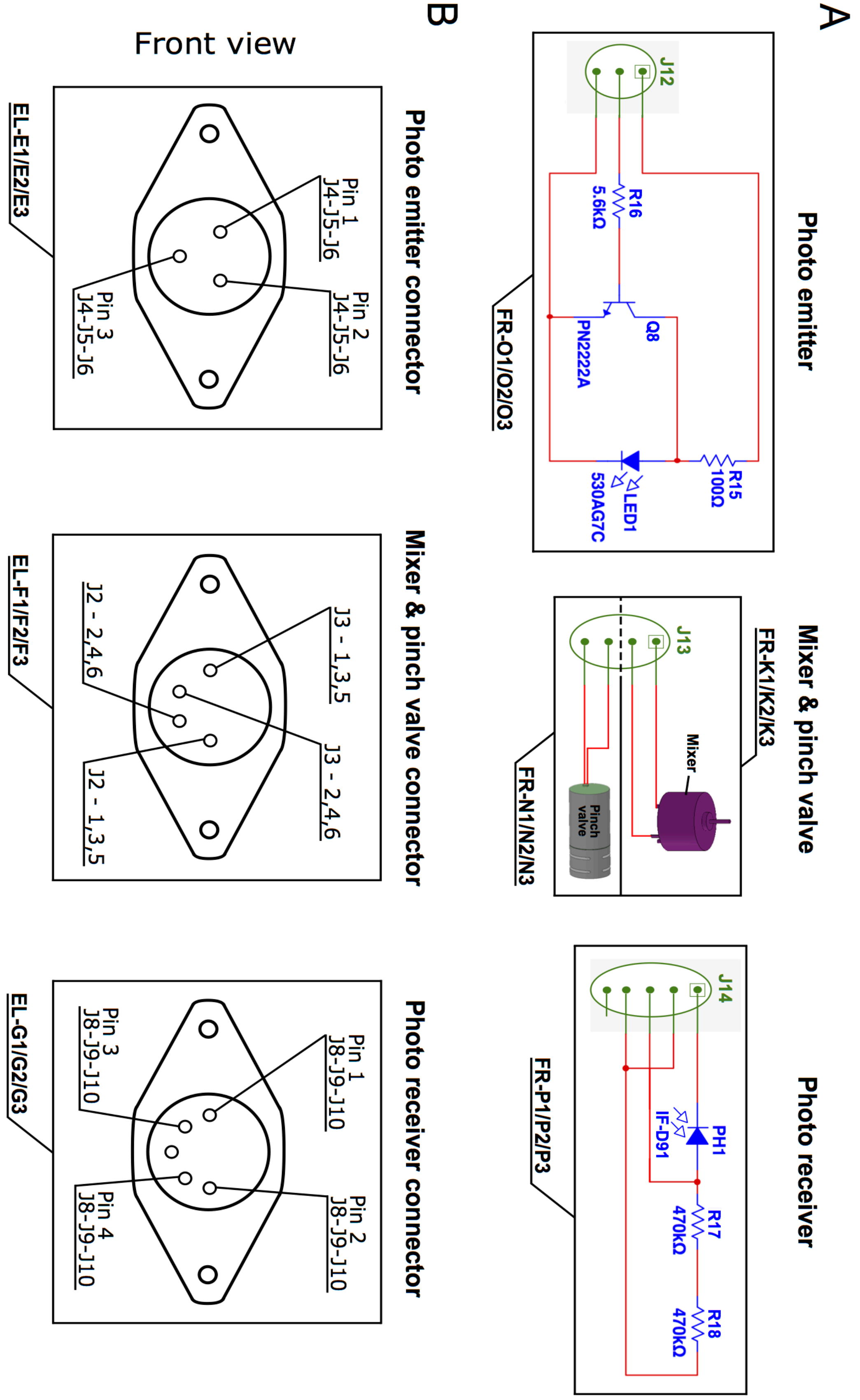

Fig. 7 - Details of the main PCB (S7 Fig.)

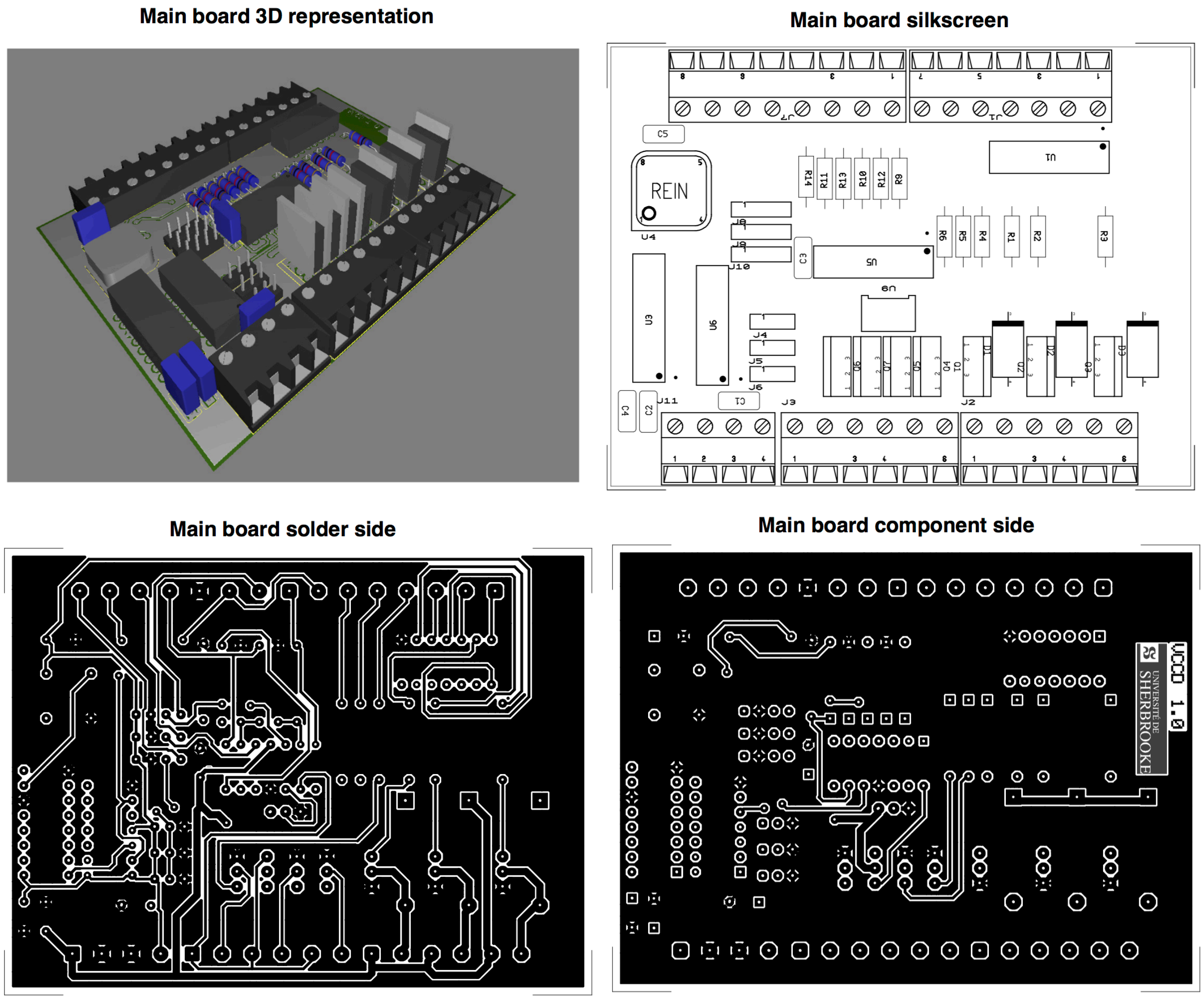

Fig. 8 - Details of the photo emitter and receiver PCBs (S8 Fig.)

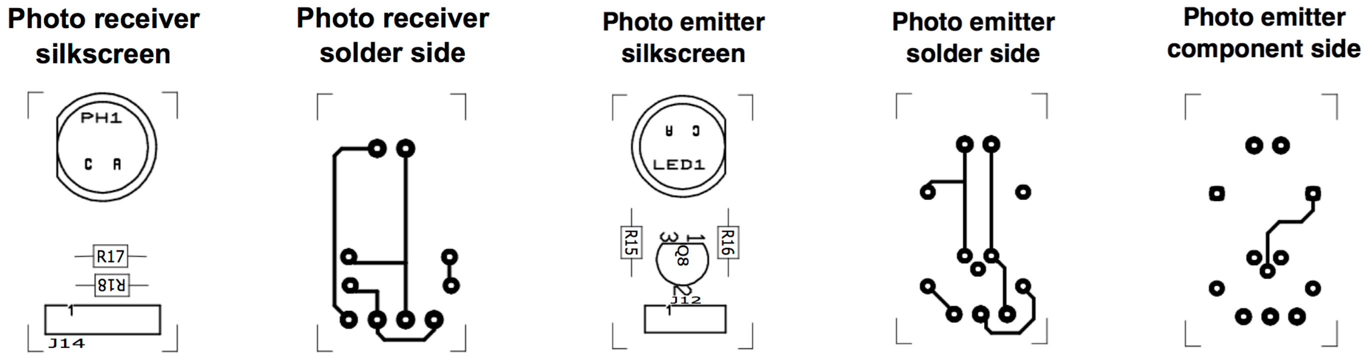

1.3 VCCD adjustments and maintenance

1.3.1 Changing culture mixing speed

To ensure the proper aeration, culture homogeneity, and nutrients availability, the VCCD includes mixing motors that rotate magnetic stir bars located inside the culture vessels. However, depending on experimental needs and the growing microorganism, the mixing speed sometimes needs to be adjusted or even turned off. The mixing speed can be adjusted by changing the voltage provided to the mixing motors by the main PCB. To do so, simply adjust the potentiometer (U9) located on the main PCB by turning the small screw clockwise or counterclockwise using a small screwdriver. A clockwise rotation will cause the mixing motors to rotate faster, while the opposite rotation will slow down the motors.

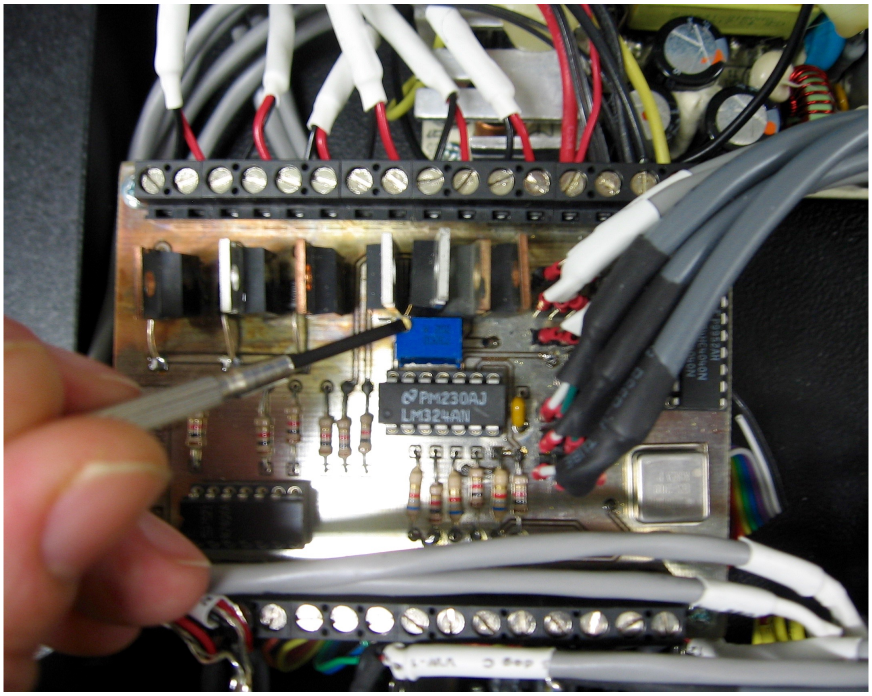

U9 Potentiometer

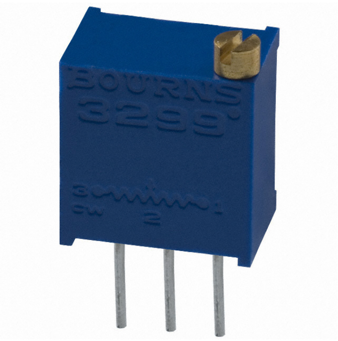

1.3.2 Adjusting pumps air flow

To perform culture dilution events and establish continuous cultures, the proposed culture system must be provided with a constant air flow. This air flow is used to create a low pressure when the pinch valve is activated, which causes a liquid displacement inside the culture vessel. The air flow is generated by a small air pump (CS-A1) that is connected to the culture system using silicone tubing. If more or less pressure is needed inside the culture system, the pump air flow can be adjusted using the small control located at the rear of the pump. To increase the air flow, simply

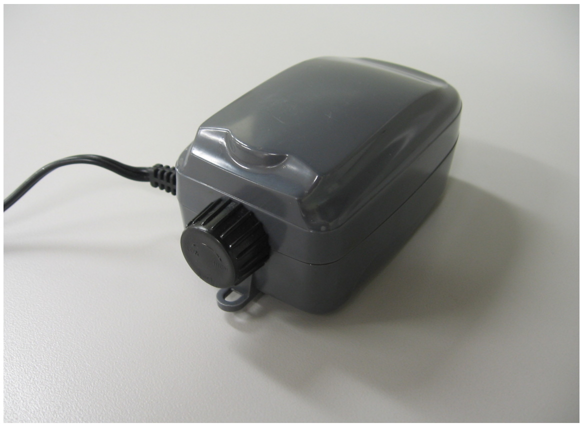

turn the control clockwise. To decrease the air flow, turn the control in the opposite direction. The maximal air flow provided by the pump is however limited to 1.5 L/min, which is sufficient for its current utilization.

1.3.3 Adjusting height of the turbidity-measuring unit support

With the proposed configuration of the front plexiglass panel (FR-B1), it is possible to adjust the height of the plexiglass assembly that supports the turbidity-measuring unit (FR-H1/H2/H3; I1/I2/I3). This adjustment may be needed if one desires to use a different type of culture vessel than the one proposed (CS-L1) or to accommodate cultures of different volumes. To adjust the turbidity-measuring unit support, simply change the position of the screws used to fix the assembly on the front panel (FR-B1).

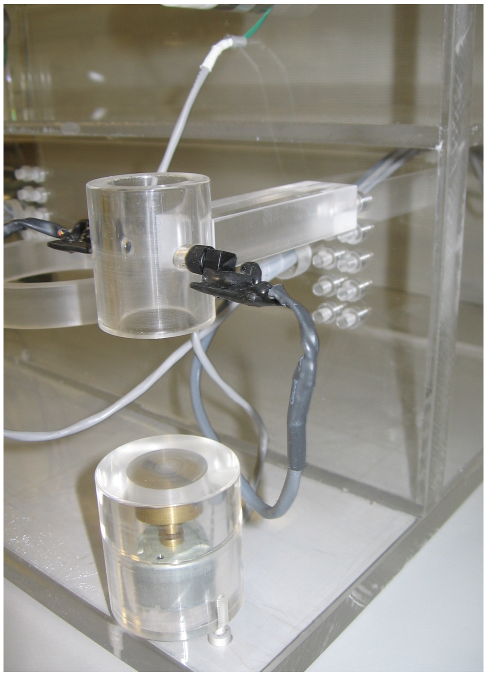

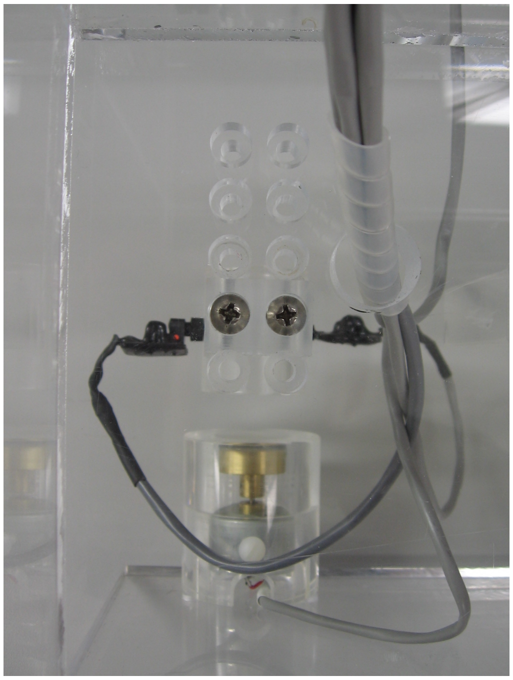

1.3.4 Washing culture system

Before autoclaving the culture system (*see* **section 1.3.5**) and beginning a new experiment, it is recommended to wash the culture system to eliminate possible contaminant. Because some components of the culture system are not supposed to be in contact with liquids, only specific parts need to be washed. For parts identification *see* **Fig. 2 and section 1.2.2**. To proceed, start by unplugging the CS-E2 tube from the 0.2 µm filter (CS-F1) and the CS-E3 tube from the other 0.2 µm filter (CS-F2). Put aside the portion of the culture system assembly that starts at CS-A1 and ends at CS-F1, as well as the one that starts at CS-F2 and ends at CS-E4 since they do not need to be washed. Unscrew the culture vessel (CS-L1) and bottles (CS-H1/H2/H3) from cap assemblies and wash them according to standard procedures. Wash tubing and cap assemblies that start at CS-E2 and ends at CS-E3 by immersing the whole assembly in a diluted bleach solution (1:49 diluted household bleach is usually recommended) and by using a standard vacuum pump. Repeat the procedure at least twice with distilled water to remove any traces of bleach inside the culture system. Remove the remaining water with a vacuum pump before reassembling the culture system to avoid the obstruction of 0.2 µm filters. The culture system is now ready to be sterilized.

1.3.5 Sterilizing the culture system

The culture system needs to be sterilized before starting a continuous culture experiment to avoid any contamination of the culture. This can be done using standard dry autoclaving procedures. However, we recommend tying a knot in the tubing located at the culture system extremities (CS-D1 and CS-E4) to make sure that water does not penetrate inside the 0.2 µm filters. Note that the proposed 0.2 µm filters are suitable for 10 autoclaving cycles and thus need to be periodically changed to ensure proper air sterilization.

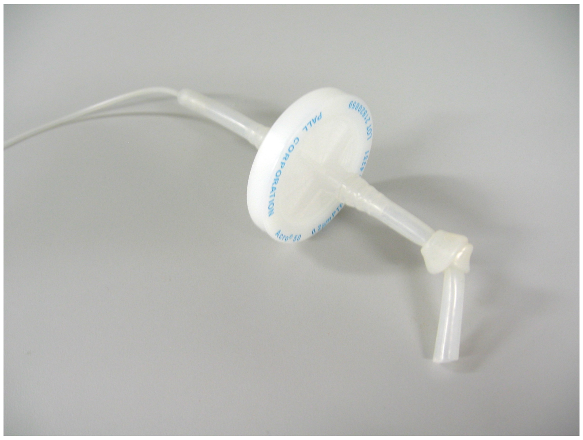

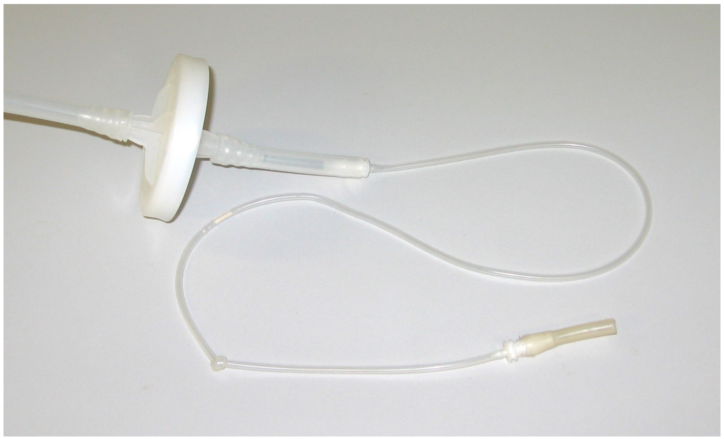

2. Software overview

2.1 Software and drivers installation

VCCD 1.0 software was designed using the NI LabVIEW Professional Development System 2009 SP1 software. LabVIEW is a graphical programming language specifically designed for hardware automation. Executable version of the software (VCCD 1.0.exe) and its dependencies were compiled in an installation package using the LabVIEW 2009 application builder. The installation package and the source code of the software are available at:
http://lab-rodrigue.recherche.usherbrooke.ca/VCCD_en/#Software_Download

To download the executable installation package, click on the link and save the attached folder (VCCD 1.0 Installation Program.zip) at the desired location on your computer. Unzip the downloaded folder, go in the Volume folder and open the setup.exe file. Then, the installation wizard will guide you through the installation process and will install all the required National Instruments drivers. After the installation, the VCCD 1.0 application folder will be created and the software is ready to be launched. The VCCD 1.0 application folder contains two important files: the VCCD 1.0.exe and the Calibration.ini. To launch the software, simply double click on the .exe file. The Calibration file contains reference voltage values printed during the calibration procedure (*see* **section 2.3.2**). The application folder also contains the National Instruments license agreement. All other files present in the folder do not need to be considered by the user for routine usage but are essential for the proper operation of the VCCD program.

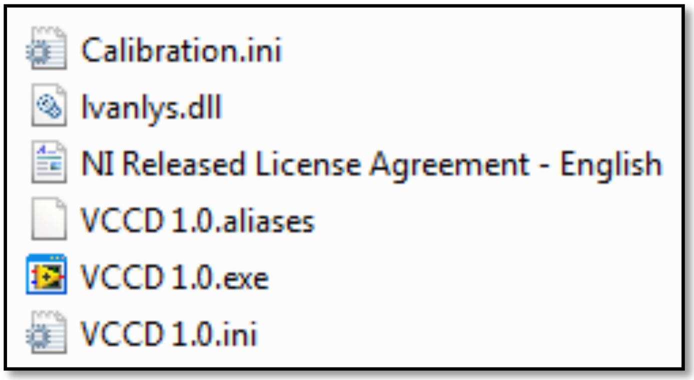

2.2 Launching the VCCD software

Once the VCCD is correctly assembled and the VCCD 1.0 software has been installed on a computer, a new experiment can be performed. To do so, the following steps must be executed in the correct order to start the VCCD software:

1- Connect the VCCD (power entry connector EL-D1) to a regular 120V power outlet with a standard 125V computer power cord (EL-J1).
2- Connect the VCCD (NI-DAQ USB-6008 EL-C1) to a USB port (2.0 or higher) of the software-installed computer using a USB cable (EL-I1).
3- Turn the power entry switch to the on position and preheat the system for at least 20 min. This preheating period is highly recommended in order to reach the thermal equilibrium of the LEDs and reduce possible signal noise.
4- Open the VCCD 1.0 application folder and double click on the VCCD 1.0.exe file to start the VCCD 1.0 software.

If the VCCD is not turned ON or not properly connected to a computer USB port, the following error message will appear:

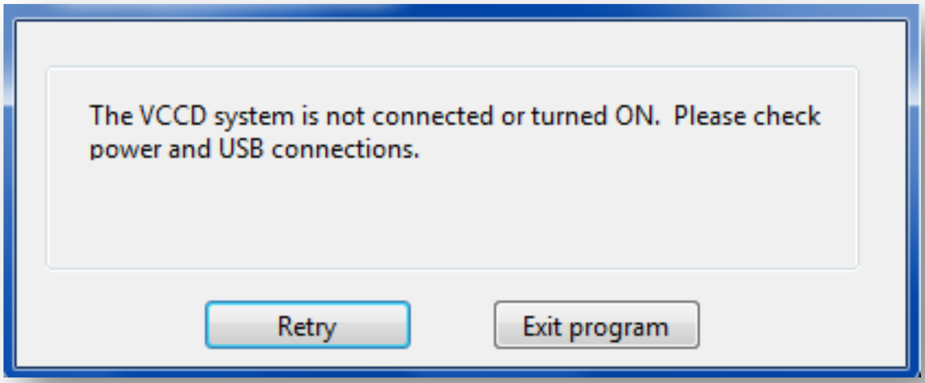

In such case, you can either verify the power and USB connections and click on the *Retry* button, or simply exit the program by clicking on the *Exit program* button. When the VCCD is properly connected, the VCCD graphical user interface (GUI) window will appear on the screen (**Fig. 9**). This window is separated in three sections (*A*, *B*, and *C*), one section per data acquisition channel,

Fig. 9 - VCCD 1.0 graphical user interface (GUI)

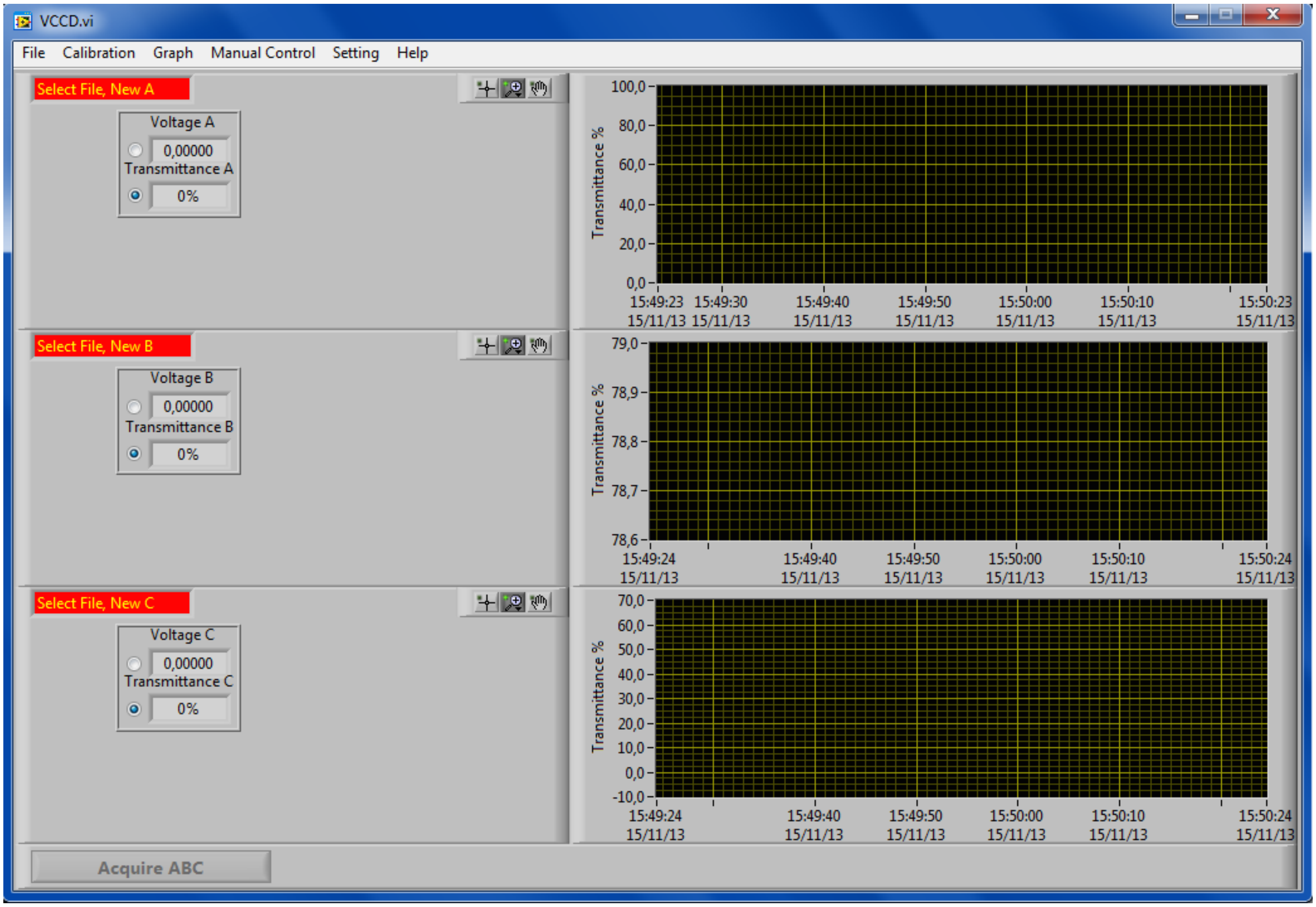

i.e. transmittance-measuring unit connected to the VCCD. Each channel section possesses two main areas: the data graph area and the experiment settings area. The experiment settings area is used to display different parameters and buttons available when a new experiment is created (*see* **sections 2.4.7, 2.4.8, and chapter 3**). This area also includes a signal box that constantly shows the acquired raw voltage signal (*Voltage A, B,* or *C*) and transmittance values (*Transmittance A, B,* or *C*). The data graph area displays in real-time the monitored voltage signal or the transmittance values. The type of signal displayed on the data graph can be switched using the selector located inside the signal box.

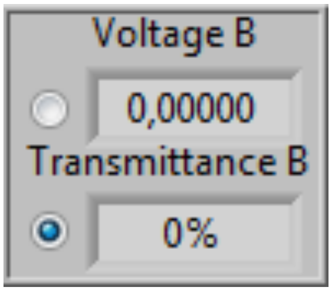

The type of signal selected inside the signal box does not affect the data monitoring procedure; meaning that both raw voltage and transmittance values are always acquired and saved (*see* **section 2.4.2**) no matter what type of signal is displayed on the graph. Also, choosing to display raw voltage values does not affect modes or settings loaded in the experiment settings area that depend on the monitored transmittance signal. These settings are not linked to the data displayed on graphs, but rather depend on the acquired and saved data.

Six different tabs are available at the top-left corner of the VCCD GUI: *File, Calibration, Graph, Manual Control, Setting,* and *Help*. To start transmittance acquisition, new data files must be

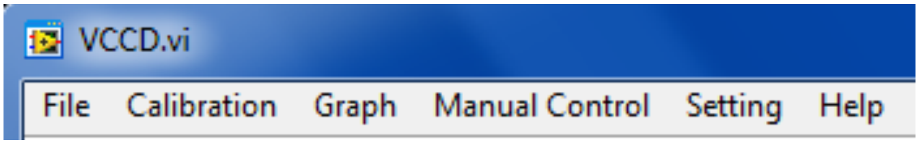

created using the *File* tab (*see* **section 2.4.1**). However, before creating new data files, some important general parameters should be configured using the other available tabs. Each of these tabs is explained in **section 2.3**. Finally, three other buttons also appears just besides each graph before and after creating a new experiment. These buttons allow rapid graphical manipulations and are explained in **section 3.1.4**.

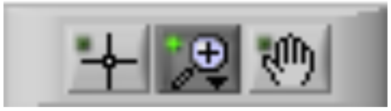

2.3 General settings

2.3.1 Data acquisition rate

The first setting to adjust prior to start a new experiment is the data acquisition rate. This parameter defines how much time separates each transmittance acquisition point or in other words how many transmittance data points are acquired per second. This setting can be modified by clicking on the *Setting* tab, and then choosing the *Acquisition rate* element. The default value (1 data point per sec) can then be changed using the small arrows and simply by entering a numerical value and pressing *OK*. It is important to note that the acquisition rate units are in seconds, so that an entered value of 60.0 means an acquisition rate of 1 data point per min. Also, the data

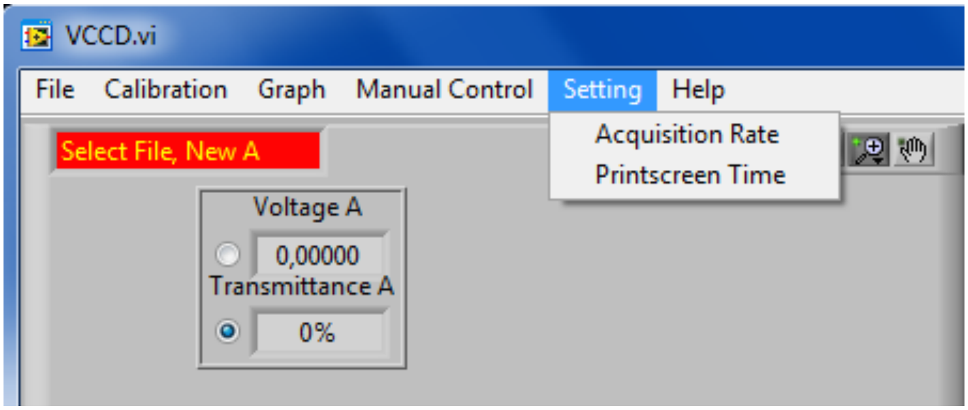

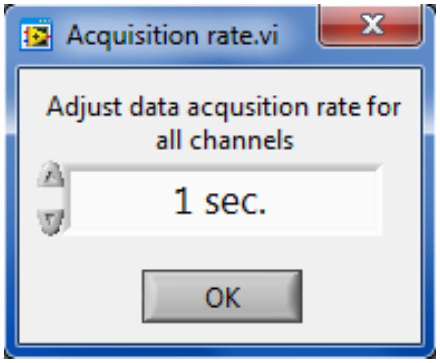

acquisition rate minimal value is restricted to 0.1 sec, and the selected value always applies to all transmittance-measuring channels. The selected data acquisition rate is however not considered when the system is performing a culture refresh. When a refresh is initiated, the data acquisition rate is automatically changed to 0.2 (5 data points per sec) to accurately record any changes in the culture transmittance, and then returns to the previously selected value. In some cases, the data acquisition rate can be very critical for the success of an experiment, e.g. experiments that require a very high transmittance resolution over a short period of time. However, choosing a high

acquisition rate (i.e. entering a low numerical value) generates large data files, especially for long period experiments, so always try to choose the acquisition rate that best fits your experiment. The acquisition data rate can be modified before and after creating a new data file, as well as in the course of an experiment, but only the last value selected before creating the data file will be displayed in the data file header (*see* **section 2.4.2**).

2.3.2 Calibration

Before creating a new data files and acquire transmittance data, it is highly recommended to calibrate the system with the appropriate solutions. This procedure does not affect the raw voltage signals detected by the system, but is used to normalize the relative % of transmittance displayed on graphs according to the reference voltage values measured with the calibration solutions. To calibrate the system, click on the *Calibration* tab, and then choose the transmittance channel to

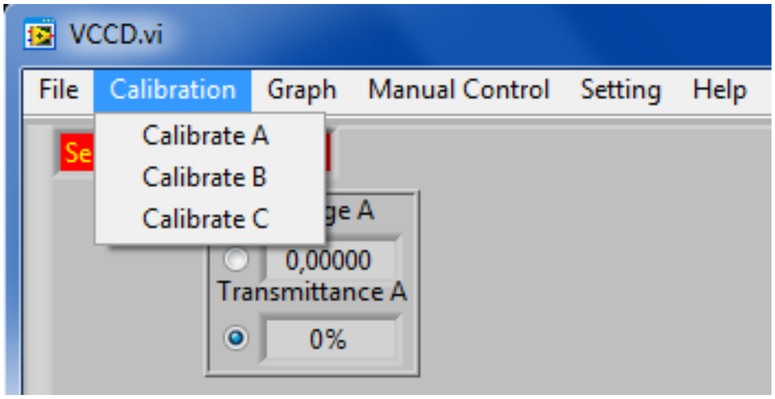

calibrate (*Calibrate A, B,* or *C*). Then, a new window will appear. Place a culture tube (must be the same type of tube as CS-L1, *see* **Table 2**) containing the solution that represents a 100% transmittance signal according to your experiment (generally fresh medium or water). Press *OK*. A

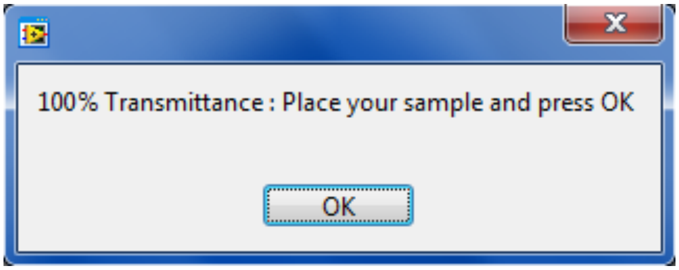

new window will then appear. Repeat the same procedure, but with the 0% transmittance signal solution (generally something that completely blocks light from reaching the photo receiver) and press *OK*. Repeat the procedure for the remaining channels. Since the three transmittance-

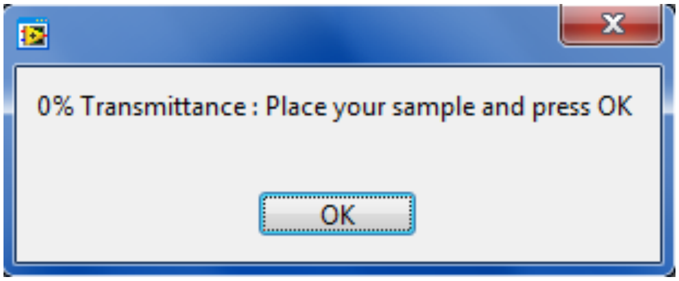

measuring units are independent, different calibration solutions can be used to calibrate each data acquisition channel. The calibration voltage values are saved in the Calibration file, which is a standard text file (.txt). The saved calibration values can be manually edited if desired and serve only as default values when the software is launched. Nonetheless, this feature can be convenient

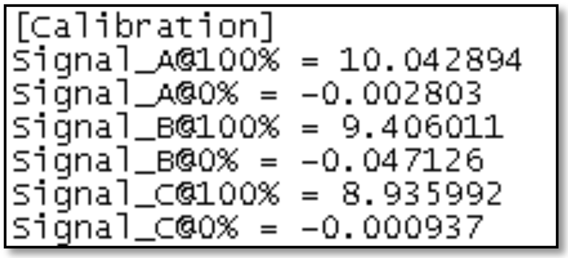

```
[Calibration]
Signal_A@100% = 10.042894
Signal_A@0% = -0.002803
Signal_B@100% = 9.406011
Signal_B@0% = -0.047126
Signal_C@100% = 8.935992
Signal_C@0% = -0.000937
```

when the user wants to execute the same experiment multiple times, since only a single calibration is necessary to perform all successive experiments. The instrument can be calibrated before and after creating the experiment data files, but only the last reference voltage values recorded before creating the data file will be displayed in the data file header (*see* **section 2.4.2**). If needed, the system can also be recalibrated during an experiment by pausing the data acquisition (*see* **section 2.4.8**) and by repeating the same calibration procedure described previously.

2.3.3 Automatic print screen

The VCCD software includes an automatic print screen parameter that generates jpeg pictures of the GUI. These pictures are automatically saved in the same folder than the created experiment data files (*see* **section 2.4.1**). The automatic print screen parameter can be set by clicking on the *Setting* tab, and then choosing the *Printscreen time* element. The default value (6 hours) can then be changed using the small arrows or simply by entering a numerical value and pressing *OK*. The value entered must be in hours. The minimal and maximal values that can be set are restricted to 1 and 24 hours, respectively. More importantly, this setting is also used to set how much time the

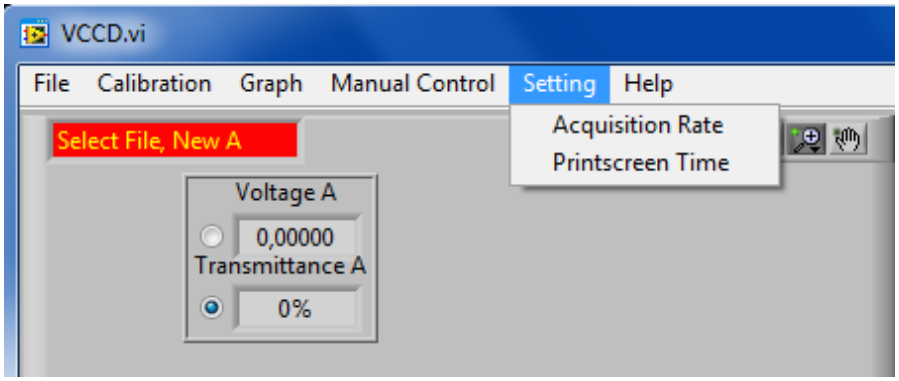

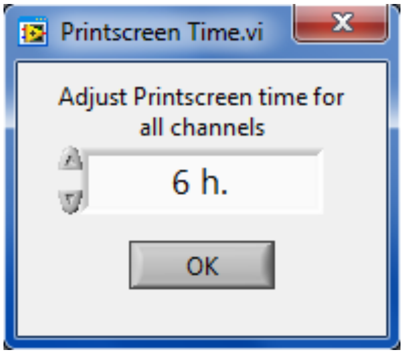

acquired data stay in the computer memory and defines the maximum time scale that can be displayed on the GUI graphs. If a small value is entered, e.g. one hour, the computer quickly clears the data from its memory, and the GUI is restrained to only display data recorded in the past hour. Because the available memory of a computer can vary, we suggest testing the maximal print screen parameter value tolerated by your computer, otherwise memory overflow could cause the software to crash.

2.3.4 Manual control of pinch valves

Pinch valves can be manually activated using the VCCD software. To activate a pinch valve, simply click on the *Manual Control* Tab, and select the desired pinch valve (*Pinch valve A, B,* or *C*). A checkmark will then be displayed on the left of the selected pinch valve in the *Manual*

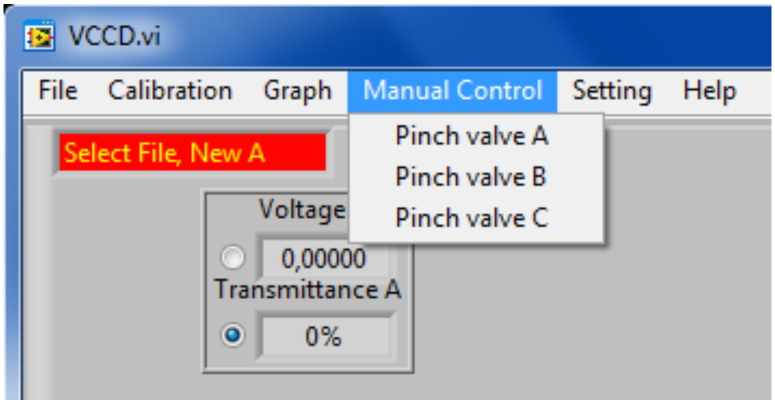

Control tab, indicating that this valve is currently activated. More than one pinch valve can be activated simultaneously, and this action can be maintained for an unlimited time. To release the

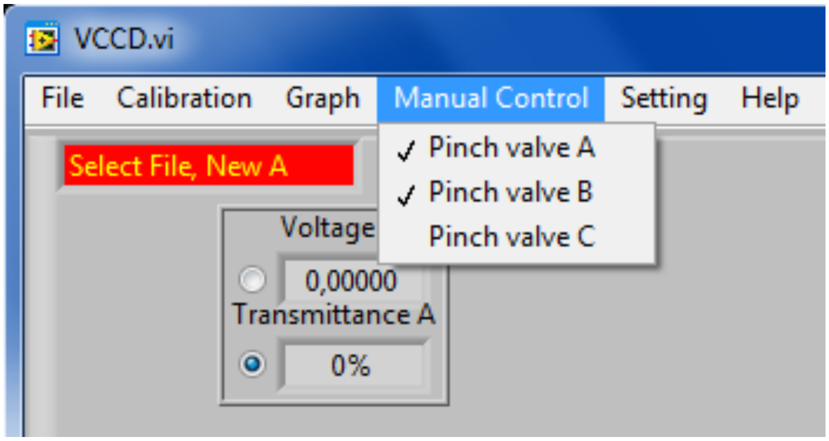

activated pinch valves, repeat the procedure to remove checkmarks beside each pinch valve name. Pinch valves can be manually activated before creating data files and beginning to acquire transmittance data, as well as in the course of an experiment. The manual control of pinch valves can be useful in different situations. For example, it can be used to verify that silicone tubing is inserted correctly in the pinch valve as depicted in **Fig. 2**. Also, it can be used to wash the culture system with sterile water before exchanging the medium bottle used for culture dilution for a different type of medium.

2.3.5 Expand a graph

By default, the VCCD software displays transmittance graphs of the three acquisition channels. However, it is possible to expand the graphical window of one channel at a time, thereby masking graphs of the other two channels. To expand a graph, click on the *Graph* tab, and then select the channel wanted to be visualized in expanded view (*Zoom IN -- A, B,* or *C*). A checkmark will

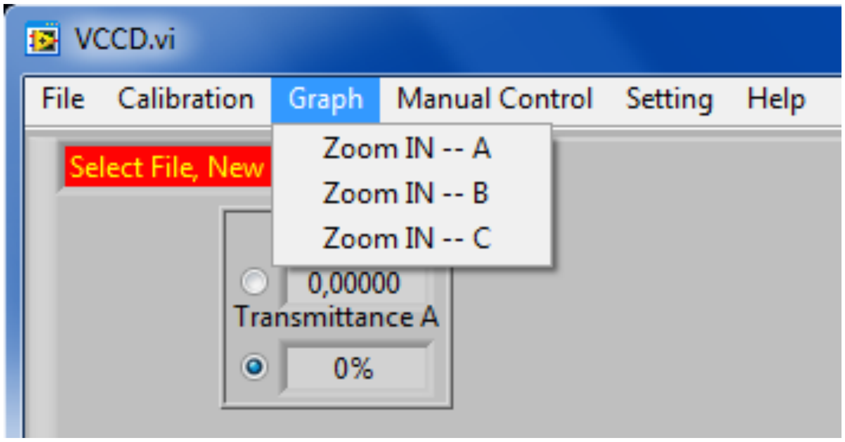

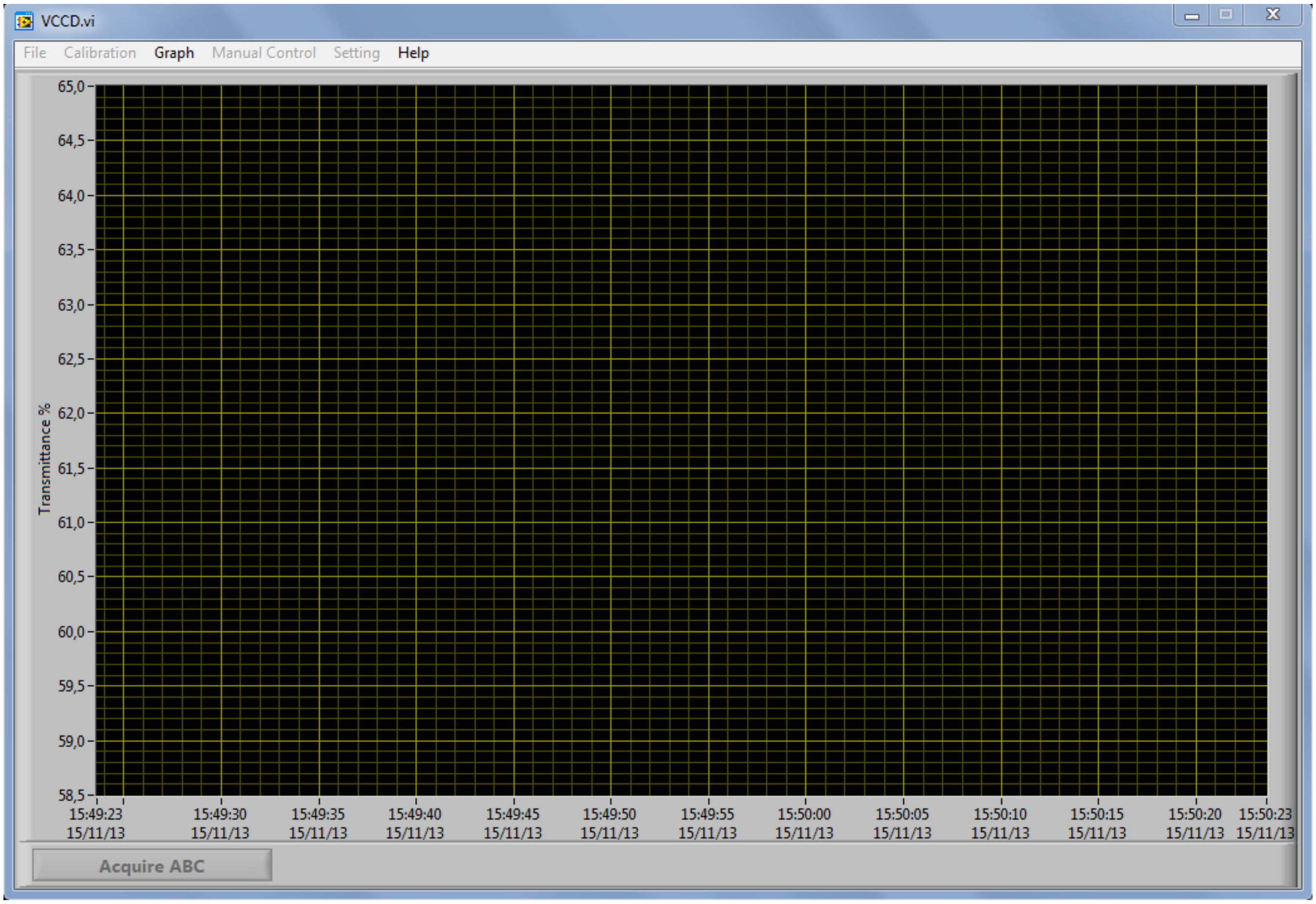

then be displayed at the left of the expanded channel in the *Graph* tab. Once a graph is expanded, the *File, Calibration, Manual Control*, and the *Setting* tabs are no longer available. To resume the expanded view, click again on the *Graph* tab, and select the check-marked channel. The expanded

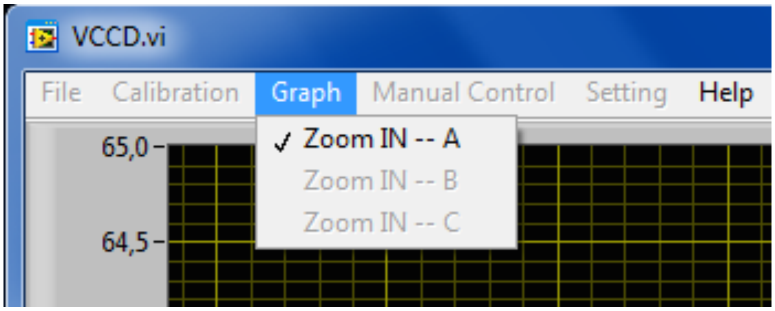

view feature can be quite convenient when a given experiment requires only one active monitoring channel, or simply if one desires an increased graphical resolution of one of the three data curves.

2.3.6 Help

The *Help* tab is used to view the National Instruments software license agreement and the authors'

information, update the VCCD software, as well as downloading the user manual. To view the license agreement, author information, and download current updates, click on the *Help* tab, and select *About...* A new window will appear, containing all information cited above. The current

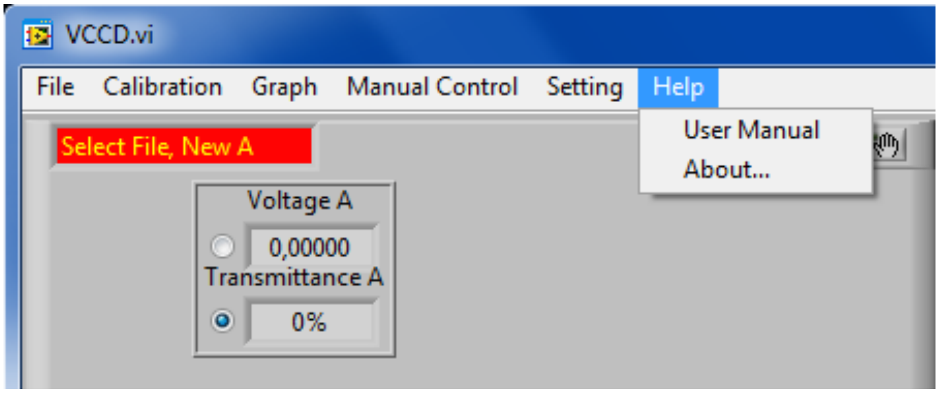

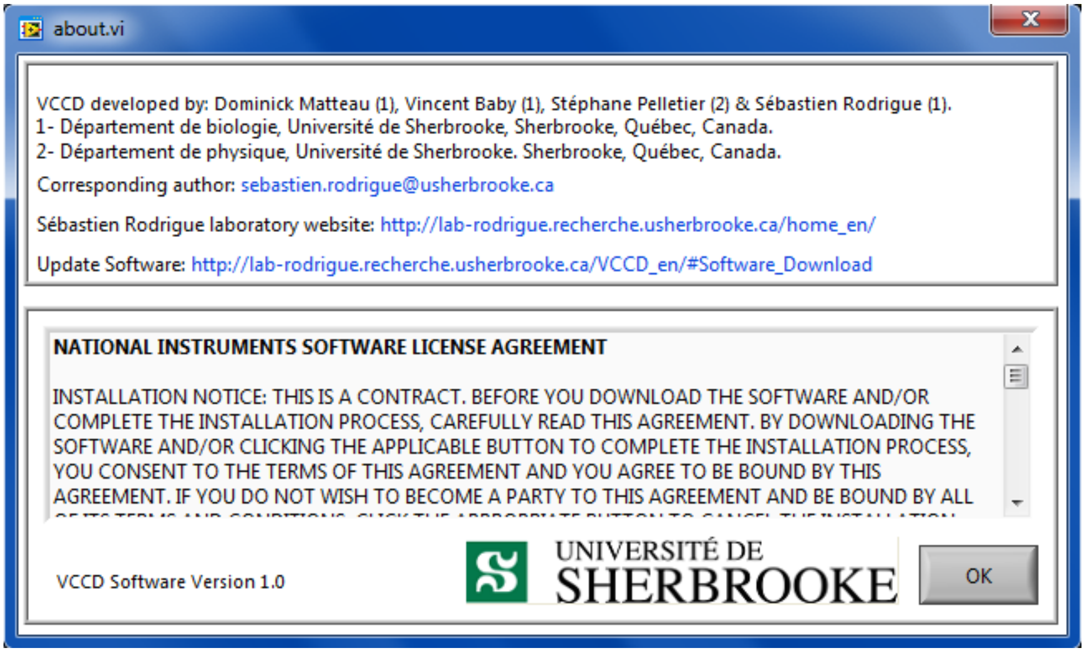

version of the software is displayed at the bottom-left of the about window. To close the window, simply click on the *OK* button located at the bottom-right. To verify if your VCCD software is up to date, click on the *Update Software* link, and check if there are available updates on the VCCD website. To view the user manual corresponding to the version of your VCCD software, click on the *Help* tab, and select *User manual*. This will open the VCCD website in which the user manual corresponding to the version of your VCCD software can be downloaded in PDF format.

2.4 Creating a new experiment

2.4.1 Creating new data files

Now that the general settings have been explored, it is time to create new data files in order to start a new experiment. To create a new data file, click on the *File* tab, and select the channel in which you want to create a new experiment (*New A, B,* or *C*). Then, a new window will appear which

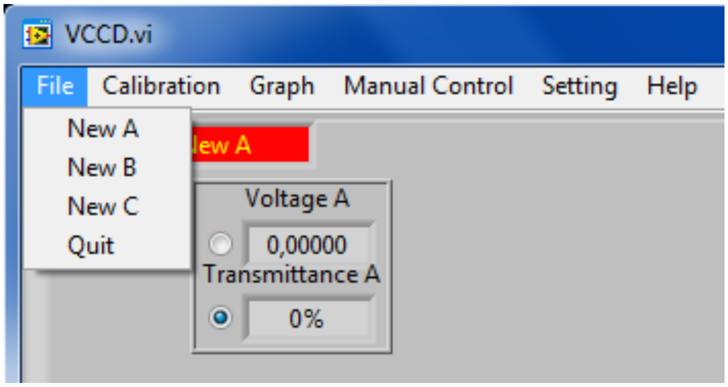

allows to select the destination path of the data file. We recommend saving the data files in the VCCD application folder, albeit data files can be saved at any location on the computer. Once a channel is selected (*New A, B,* or *C*), a configuration window will appear on the screen. This

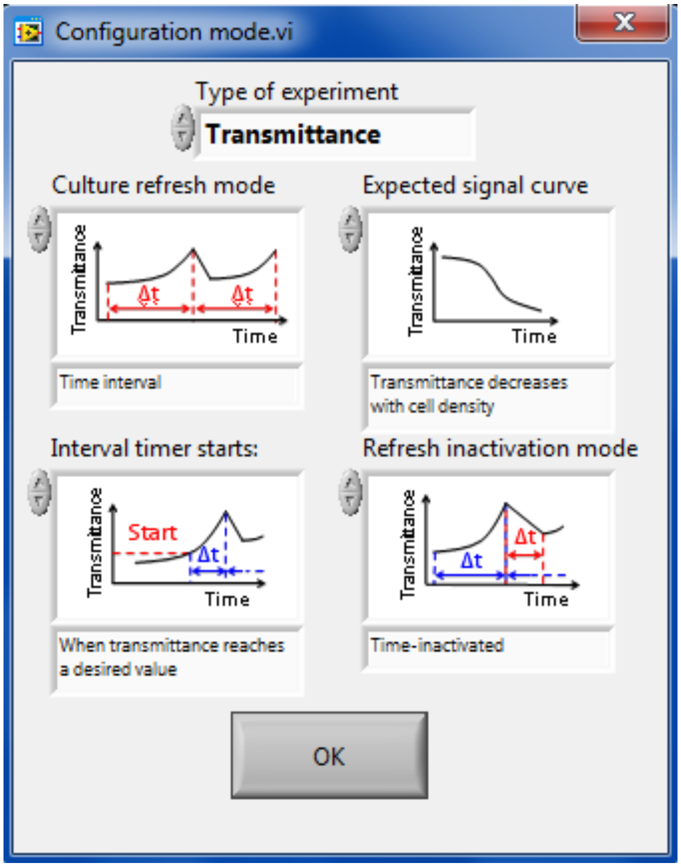

window displays the experiment modes and parameters currently available with the VCCD software. The meaning of each of these modes, as well as how to select them is explained in details in **sections 2.4.3 to 2.4.6.** After that the desired modes and parameters have been selected for a given channel, the appropriate settings and action buttons are loaded in the corresponding experiment settings area (*see* **sections 2.4.7, 2.4.8 and chapter 3**). A new data file must be created

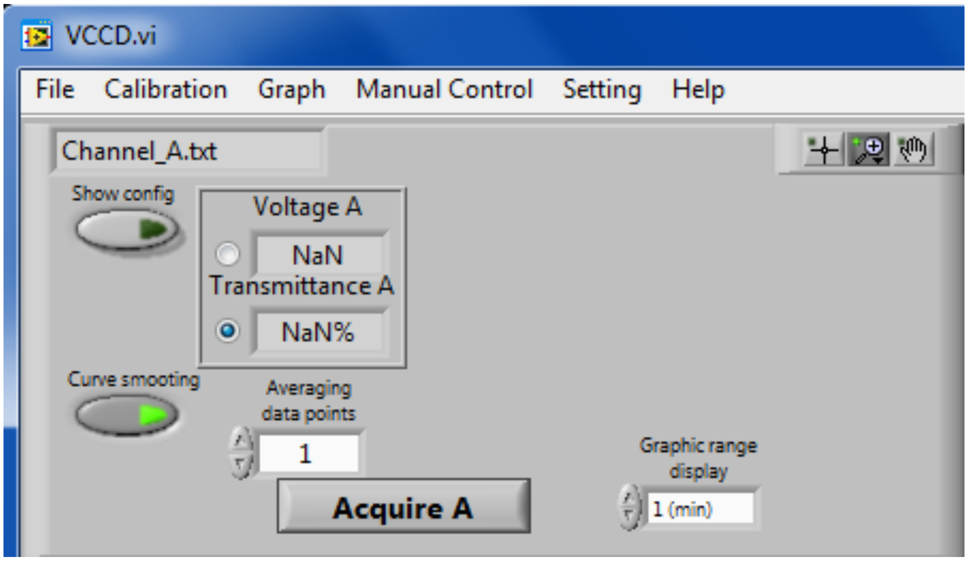

for every channel in which the user wants to perform an experiment. The data file name is displayed at the top-left of the corresponding experiment settings area. Since the pinch valves are controlled independently, different culture refresh modes can be loaded simultaneously. This feature allows one to perform different types of experiment at the same time, and with different options and parameters for each channel. If the loaded modes of one channel need to be modified after or in the course of an experiment, it is always possible to create a new date file with different options and restart data acquisition file (*see* **section 2.4.8**) without resetting the VCCD software.

2.4.2 Data file format

The data file consists of a standard text file (.txt) that includes two important parts: the header and the monitoring data section. The header comprises all information regarding the associated experiment: the file creation date, calibration voltage values, data acquisition rate, print screen parameter value, and the selected experiment modes and parameters. It is important to note that values displayed for the data acquisition rate, the print screen parameter, and the calibration signals are those entered before creating the data file. In other words, once the data file header is created, it cannot be modified using the VCCD software, but could easily be modified after the

experiment using a text editor. Every minute, the monitored data section prints the recorded transmittance and voltage signal values, as well as other pertinent information regarding the ongoing experiment. Each record is represented by one line, and the number of record per second

```
[Header]
Created file date = "2015 April 30"
Signal_A@0% = 0,000000
Signal_A@100% = 6.000000
Acquisition rate (msec) = 1000
Printscreen time (h) = 6
Type of experience = "Transmittance"
Culture refresh mode = "Real-time feedback loop"
Expected signal curve = "Transmittance decreases with cell density"

[Data]
2015-04-30	10:46:18	86.40	4.85	0	0	0
2015-04-30	10:46:19	86.41	4.85	0	0	0
2015-04-30	10:46:20	86.40	4.85	0	0	0
2015-04-30	10:46:21	86.38	4.85	0	0	0
2015-04-30	10:46:22	86.45	4.85	0	0	0
2015-04-30	10:46:23	86.37	4.85	0	0	0
2015-04-30	10:46:24	86.43	4.85	0	0	0
2015-04-30	10:46:25	86.41	4.85	0	0	0
```

is determined by the data acquisition rate. Each printed line is composed of 7 tab-separated fields. The first two fields represent the date and the time of the record, respectively. The third and the fourth fields indicate the recorded relative transmittance value and the raw voltage signal, respectively. The last three fields are related to culture refreshes executed during an experiment. These fields represent respectively the number of culture refreshes executed in the experiment, the number of times the pinch valves was activated during the current refresh, and the total number of pinches performed in the experiment (*see* **section 3.2.2**).

2.4.3 Type of experiment

With the present photo emitter and photo receiver configuration, the VCCD is only adapted to monitor transmittance signals. Nevertheless, the VCCD software was designed to also support fluorescence signal acquisition, a mode that would however require some modifications in the photo emitter and receivers units in order to detect fluorescence signals. The type of signal monitored by the VCCD can be modified in the *type of experiment* field, which is the first option available in the configuration window when a new data file is created (*see* **section 2.4.1**). In the *Transmittance* mode, only light emitted by the photo emitter is detected by the system, whereas in the *Fluorescence* mode any possible light source is detected since the photo receiver is no longer synchronized with the LED frequency. To select the type of experiment in the configuration

window, simply click on the arrows at the left of the *type of experiment* field and select the appropriate mode. By default, the type of experiment is set to *Transmittance*.

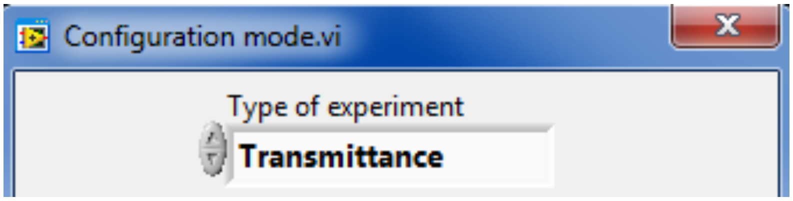

2.4.4 Culture refresh modes

The second available option when a new data file is created is the culture refresh mode. This option defines the general behavior of the VCCD (**Fig. 10A**), or more precisely what causes the activation of pinch valves. With the current software version, four modes are available. The simplest mode is the *None* culture refresh mode. This mode is used to measure transmittance of a batch culture without performing any culture refresh. With this mode, there is no need to assemble the pump and culture refresh apparatus on the VCCD frame (*see* **section 1.2.2**) since no culture refresh is performed. The second available culture refresh mode is the *Real-time feedback loop* mode. Using this mode, the culture is refreshed at a desired transmittance value until a second value is reached. The third available mode, the *Threshold-activated* mode, is similar to the *Real-time feedback loop*, except that the system keeps refreshing the culture for a specified time instead of refreshing until a specific transmittance value is detected. The last culture refresh mode, the *Time interval* mode, is used to refresh the culture periodically and make the VCCD behave like a chemostat system. When this mode is selected, two other options are available in the configuration window (**Fig. 10B**). These options are explained in **section 2.4.6**. To select the culture refresh mode, simply click on the arrows at the left of the *Culture refresh mode* box in the configuration window and select the appropriate mode. By default, the culture refresh mode is set to *Real-time feedback loop*.

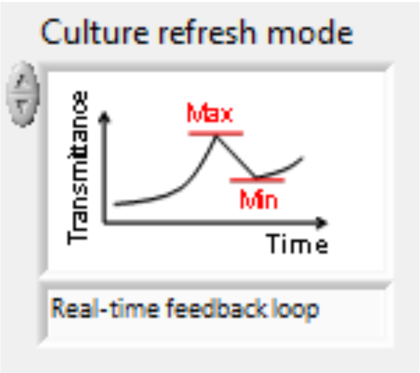

Fig. 10 - Illustration of available continuous culture modes (A) and *Time interval* specific options (B) used to maintain cell growth (Fig. 4).

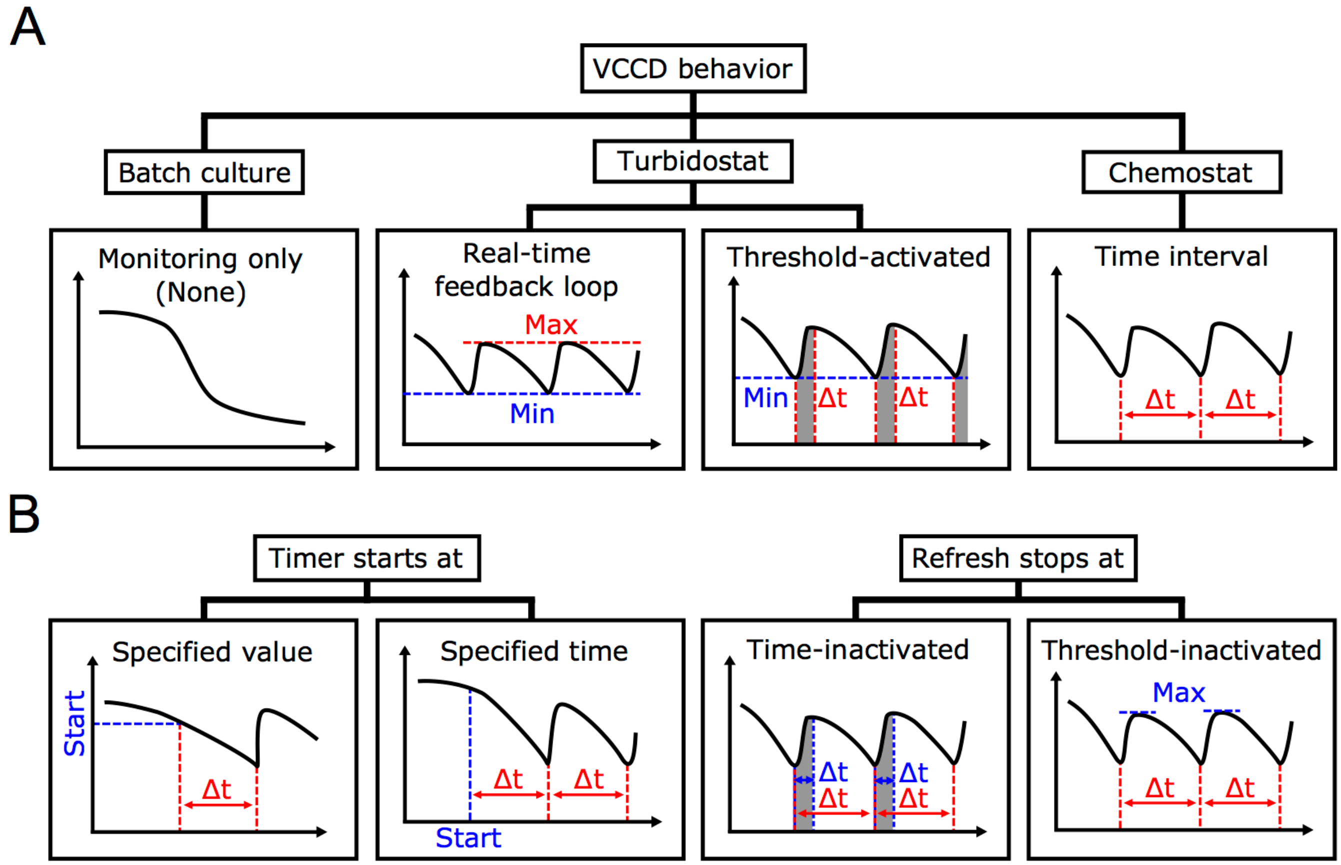

2.4.5 Expected signal curve

With every culture refresh mode selected, except of the *None* mode, an expected signal curve parameter needs to be defined in the configuration window. This parameter indicates to the software what type of signal curve is expected to be recorded as the microorganisms grow inside the culture vessel, and slightly modifies the logic of the chosen culture refresh mode. In general, culture transmittance declines as the cell population grows and gradually blocks the incident light (e.g. *E. coli* growing in LB broth). However, some microorganisms do not increase culture opacity when growing (e.g. *Mycoplasma sp* and *Mesoplasma sp*). Under these circumstances, our selected transmittance-measuring units allow using phenol red as a growth medium pH indicator to monitor the acidification caused by metabolic activity and proliferation of the cells. As the pH decreases, the absorbance of phenol red at 560 nm drops, which results in a concomitant increase of the 560nm transmittance. To set the expected signal curve parameter, simply click on the arrows at the

left of the *Expected signal curve* box in the configuration window and select if the *Transmittance increases with cell density* or *decreases with cell density* according to the studied microorganism.

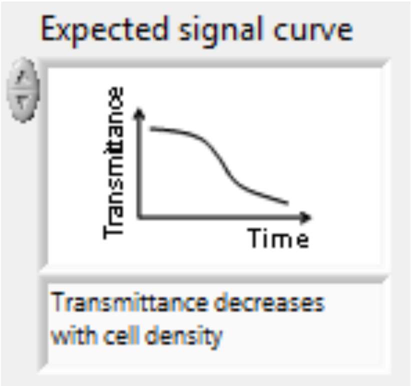

2.4.6 *Time interval* specific modes

When the *Time interval* mode is selected in the configuration window, two additional options must be selected to start a new experiment (**Fig. 10B**). The first one is the interval timer start parameter. At the beginning of an experiment, the interval timer that counts the time separating two culture refreshes is not yet activated and needs a specified signal to be initialized. This signal can be a transmittance value (*Transmittance reaches a desired value*), a specific time of the day (*At a user specified time*), or simply a manual activation (*Manually*). The second option to choose is the refresh inactivation mode. This mode decides what kind of signal releases the pinch valve during a culture refresh. This signal can either be a transmittance threshold (*Threshold-inactivated*) like in the *Real-time feedback loop* mode or a maximum amount of time elapsed during the refresh procedure (*Time-inactivated*) like in the *Threshold-activated* mode. To select the *Time interval* specific modes, click on the arrows at the left of the *Interval timer starts:* and *refresh inactivation mode* boxes, select the appropriate parameters, and press *OK*.

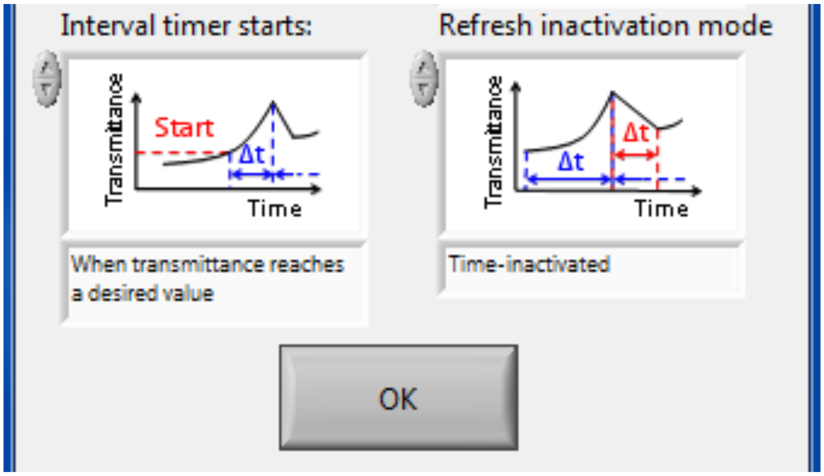

2.4.7 Show experiment configuration

Once the data file has been created for a given channel, it is possible to review the experiment modes and parameters selected inside the configuration window using the *Show config* button. This button is always displayed immediately below each file name in the experiment settings area. To review the selected experiment modes and parameters, simply click on the *Show config* button and a new window will appear on the screen. Click again on the button to remove this window from the screen.

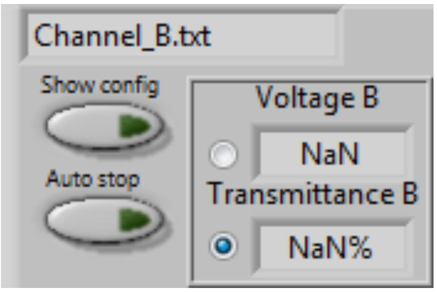

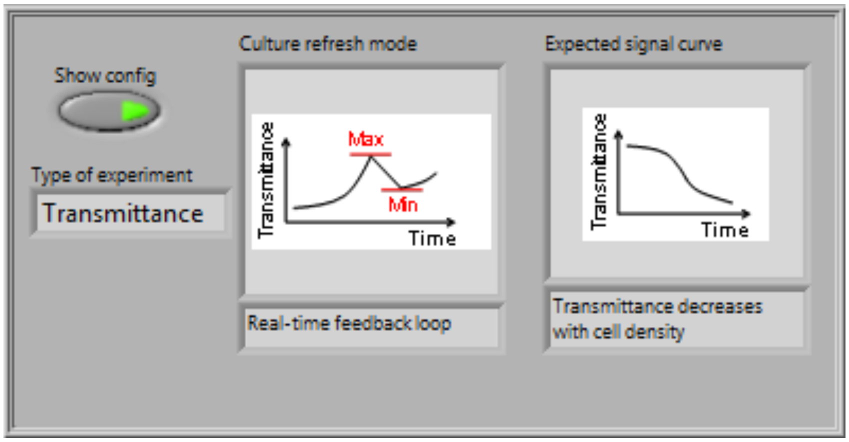

2.4.8 Acquire button

Once the data file has been created for a given channel, a new experiment is ready to be launched and transmittance data are ready to be acquired. To start data acquisition for a given channel, click on the corresponding *Acquire* button (*A, B or C*) located at the bottom of the experiment settings area. Then, the monitored data will instantly be displayed on the associated graph. To stop or pause data acquisition, click again on the button. It is also possible to simultaneously start data acquisition for the three channels by clicking on the *Acquire ABC* button located at the bottommost region of the program window.

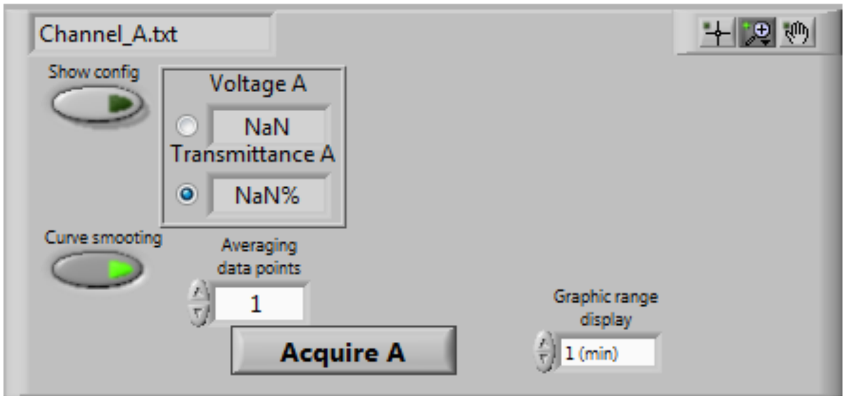

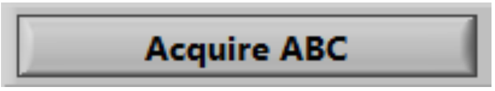

3. Advanced experiment parameters

3.1 Advanced graphical settings

The advanced graphical settings allow the user to modify graphs x-axis and y-axis resolution (*see* **section 3.1.1 and 3.1.2**), as well as to smooth data curves (*see* **section 3.1.3**). These features are available in all culture refresh modes.

3.1.1 Graphic range display

The graphic range display setting sets the x-axis resolution of a given data graph during data acquisition. In other words, it defines what is the timescale displayed, or for how long the acquired data are still visible on the graph. The default graphic range display value is set to 1 min, which is also the minimal value accepted by the program. This setting is located immediately at the right of the *Acquire* button. To modify the x-axis resolution of a given channel, enter the desired numerical

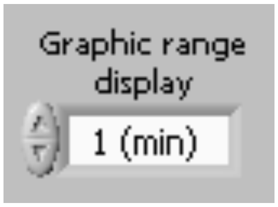

value (in minutes) in the *Graphic range display* box or simply use the arrows. If the entered value is above the amount of time elapsed since the beginning of data acquisition, right click on the data graph in order to open a new option window. This window contains some of the LabVIEW generic graphical options (*see* **section 3.1.2**). Select *AutoScale X* to check this element and the data graph will automatically be resized according to the entered graphic range display value. If the entered value is below the amount of time elapsed, the *AutoScale X* element will be automatically unchecked and the x-axis will be resized to this value. It is very important to note that the maximum time scale displayed on the graph can never exceed the print screen time parameter (*see* **section 2.3.3**), which is used to define how much time the acquired data stay in memory. The maximal and minimal x-axis represented values can also be manually modified by double clicking on them and changing their respective number, provided that the *AutoScale X* option is unchecked.

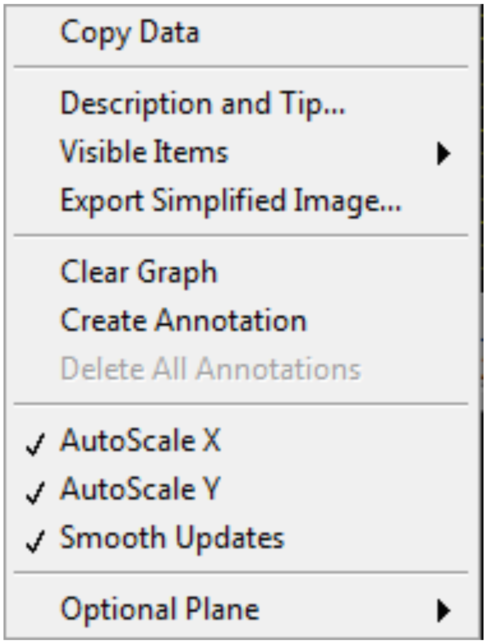

3.1.2 Other graphical options

By default, the LabVIEW platform includes different parameters available for every graph present in a developed program. One of those is the *AutoScale X* option (*see* **section 3.1.1**). The same option is also available for the y-axis (*AutoScale Y*), and is activated by default to restrain the y-axis to the range of data acquired since the beginning of an experiment and thus show the most appropriate graphical resolution. Nonetheless, the y-axis resolution can be changed in a way similar to the x-axis. To do so, right click on the desired graph and uncheck the *AutoScale Y*

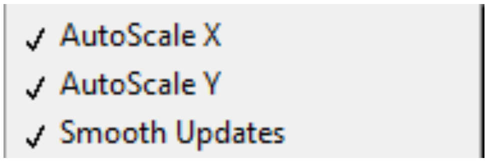

element. Then, the maximal and minimal y-axis represented values can be modified by double clicking on them and changing their respective number. This will cause the y-axis to scale up or down according to the new values. Using the same graph option window, it is also possible to remove the x- and y-axis from the data graph area. This can be done by unchecking the *X Scale* or *Y Scale* elements in the *Visible Items* tab. Additionally, one can export an simplified image of the

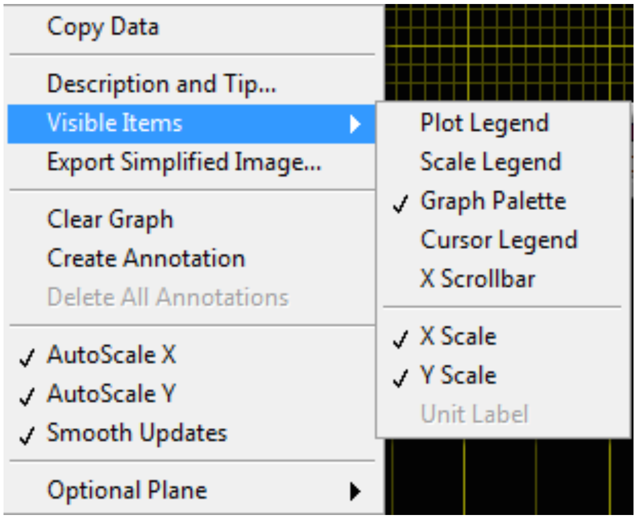

data graph by clicking on the *Export Simplified Image* tab. This will open a new window in which format of the exported image as well as exportation path must be defined. To remove the grid from

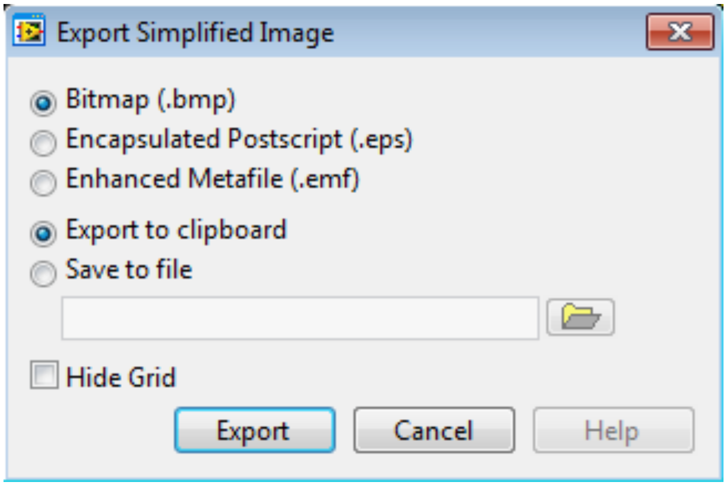

the exported graph image, simply check the *Hide Grid* option on the Export Simplified Image window. Finally, three other buttons can be useful for data visualization. These buttons are located at the top-right of each experiment settings area. When activated by a click, the first of these

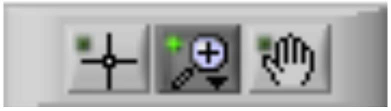

buttons (from left to right) transforms the normal cursor into a pointer, which can be useful to point specific regions on the data graph. The second one opens a new window when pushed, and allows the user to manually change the visible portion of a data graph without entering any numerical values in the x- or y- axis. This feature can be useful to quickly zoom on interesting

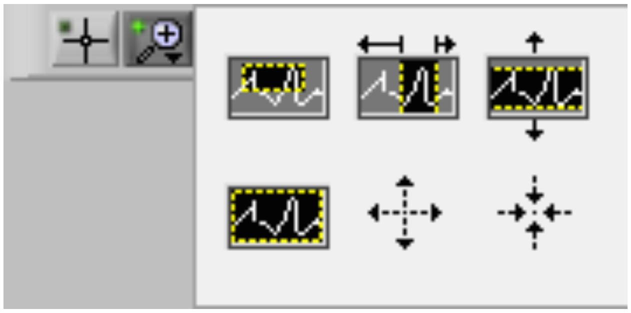

portions of the graph and is available either during or after data acquisition (*Acquire* button), but the *Autoscale X* and *Y* options must be deactivated (unchecked) when used in the former situation. The last button changes the normal cursor to a grabbing hand when activated, which enables the user to scroll throughout the data graph. Once again, this feature can be used during or after data acquisition, provided that the *Autoscale X* and *Y* elements are deactivated during data acquisition.

3.1.3 Curve smoothing

Most of data acquisition systems can be less or more affected by noise, and the source of such noise can be either natural (bacterial flocculation or heterogeneous medium for instance) or artificial (thermal noise, static noise, power source noise…). One of the most frequent ways to reduce potential noise affecting data acquisition is the data smoothing method. While many different algorithms exist to smooth data points, one simple way is by averaging each data point by a desired number of preceding points. This method reduces local signal fluctuations generated by noise sources and therefore creates a more smooth data curve. The curve smoothing feature can be activated by clicking on the *Curve smoothing* button, and by choosing the number of data points

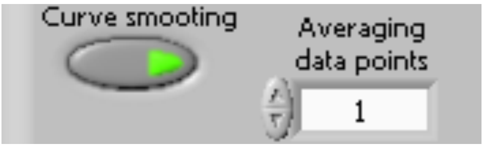

included in the averaging calculation. This value can be entered manually inside the *Averaging data points* box (minimal value is 1) or changed using the arrows. The curve smoothing feature can be activated or deactivated during an experiment without any problem. While this feature can be useful in many situations, always remember that the smoothing effect greatly depends on the data acquisition rate (*see* **section 2.3.1**). For example, choosing to average on 20 data points when using a data acquisition rate of 2 min can be a risky choice since every data point will be affected by the data acquired during the previous 40 min. Depending on the experiment, this could mask

interesting signal fluctuations that would normally be seen without smoothing data points. It is therefore strongly recommended to use the data smoothing feature in combination with a low data acquisition rate. Moreover, testing the impact of this option on a test solution prior to the real experiment would be a good idea.

3.2 Refresh settings common to all culture refresh modes

Depending on what modes and parameters are selected in the configuration window for a given acquisition channel (*see* **sections 2.4.4 to 2.4.6**), different settings and buttons controlling culture refreshing can be loaded in the corresponding experiment settings area. While some of these elements are shared between all culture refresh modes, some are unique to certain modes. This section of the manual explains the purpose of settings associated with culture refreshing that are common to all culture refresh modes, with the exception of the *None* mode that does not support culture refreshing.

3.2.1 Enable refresh

When a new experiment is created, the *Enable refresh* button appears under the rapid graphical manipulation buttons. When activated, this button signals to the program that all available parameters have been correctly set and that the system is ready to perform culture refreshes. When

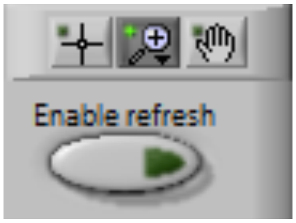

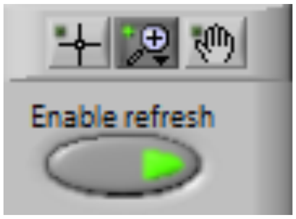

starting data acquisition, this button is set to off by default, which allows the user to adjust culture refresh settings or other parameters before allowing the software to execute culture refreshes. When the *Time interval* culture refresh mode is loaded, this button is automatically activated upon manual interval timer initialization (*see* **section 3.4.2**).

3.2.2 Refresh and pinch counters

To follow the status of the pinch valve and the evolution of the continuous culture, three counters are visible underneath the *Enable refresh* button. The first of these counters, the *Total refresh* counter, indicates the number of culture refreshes executed since the creation of the data file and the beginning of the experiment. Because certain refresh options allow the pinch valve to perform multiple pinches in a single refresh cycle, the second counter (*Pinch in current refresh*) displays the number of valve pinches executed in the current refresh cycle. The last counter (*Total pinch*) shows the total number of pinches performed since the start of an experiment. The values of these

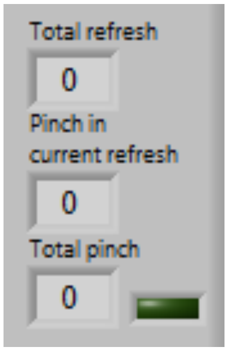

counters are constantly saved in the data portion of the data text file throughout and experiment, and represent fields number 5, 6, and 7, respectively (*see* **section 2.4.2**). Finally, when the specified conditions are met by the program to perform a culture refresh cycle, a small green LED indicator located besides the *Total pinch* counter switches-on to warn the user that culture dilution is imminent.

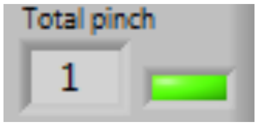

3.2.3 Maximal number of refreshes

If a certain number of refresh cycles must not be exceeded, the program includes the *Max refresh* button that can automatically inactivate the *Enable refresh* button (*see* **section 3.2.1**). This button

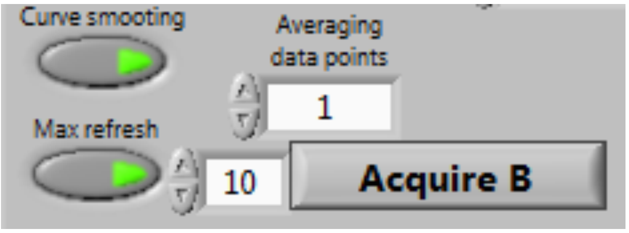

is located immediately at the left of the *Acquire* button. When activated, it opens a small field in which the maximal number of refreshes tolerated by the program can be specified. When this number of refreshes is exceeded, the *Enable refresh* button is inactivated. In other words, if the maximal number of refreshes is set to 5 and the number of refreshes performed since the start is equal to this number, the system can still perform one more refresh. The default value is set to 10 refreshes. Note that this button can only block the refreshing capacity of the system, therefore data acquisition is not affected and continues even after the maximal refresh threshold is reached.

3.2.4 Stop refresh program

Another feature common to all culture refresh modes, with the exception of the *None* mode, is the stop refresh program. Like the *Max refresh* button, this setting enables the user to program the inactivation of the *Enable refresh* button, but at a specific time and date instead of a certain number of refreshes. To activate the stop refresh program for a given channel, click on the *Auto stop* button located just under the *Show config* button in the experiment settings area. This will open a new window at the top-right of the corresponding data graph. Then, enter the desired date and time, or click on the small calendar icon to open a calendar containing more options. When activated, the stop refresh program is by default set to current time plus one day.

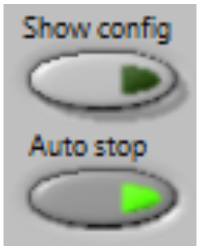

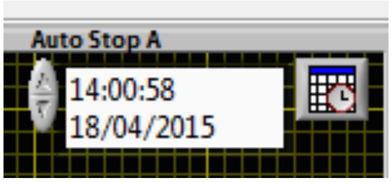

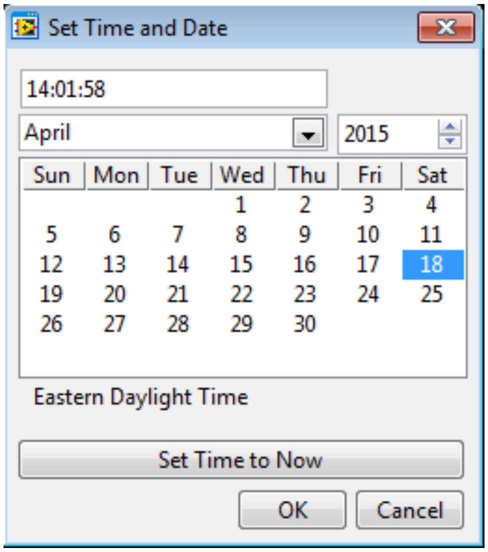

3.2.5 Pinch pause

The pinch pause parameter sets the amount of time that separates each pinch event if multiple pinch events are programmed to happen or need to be executed within a refresh cycle. The pinch pause setting can be set in the *Pinch pause* box that appears just over de *Graphic range display*

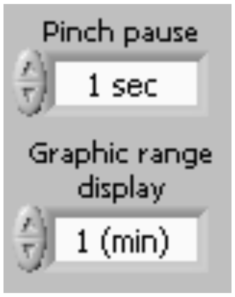

box when a new data file is created. This can be done by entering the desired number (in sec) or using the arrows. The default value is set to 1 sec, and the minimal allowed one is fixed to 0.1 sec. The pinch pause may be quite important in certain circumstances since it ensures a time delay in which the newly added fresh medium can properly mix with the culture before allowing a new pinch event to happen.

3.3 Refresh settings common to multiple culture refresh modes

This section of the manual explains the purpose of settings displayed in the experiment settings area that are associated with culture refreshing and common to some but not all culture refresh modes (*see* **sections 2.4.3 to 2.4.6**).

3.3.1 Minimum and maximum transmittance thresholds

When the VCCD is in *Real-time feedback loop* or *Threshold-activated* mode, transmittance threshold(s) must be set to program automatic culture refreshes. In the former mode, two different thresholds must be set since the culture is refreshed at a desired transmittance value until another value is reached. These thresholds are respectively named the minimum and the maximum transmittance thresholds. Depending on which of the two *Expected signal curves* was previously selected (*see* **section 2.4.5**), these thresholds can interchange their respective role. For example, if the *Transmittance increases with cell density* parameter is chosen, the maximum transmittance threshold signals the beginning of a refresh cycle if reached, whereas the minimum threshold releases the pinch valve and stops the culture dilution event. On the contrary, if the *Transmittance decreases with cell density* parameter is selected, the minimum transmittance threshold starts the refresh cycle when exceeded, whereas the maximum threshold stops it. If the VCCD is set to the *Threshold-activated* culture refresh mode, only one of the transmittance thresholds is used because the culture is refreshed for a specified time instead of until a specific transmittance value is detected. This threshold can either be the minimum or the maximum transmittance threshold depending on the previously selected *Expected signal curve*. The minimum and maximum transmittance thresholds can be adjusted by entering the desired percentage in the *Transmittance Min* and *Transmittance Max* boxes, respectively. These boxes are located at the right of the type of signal box when the *Real-time feedback loop* or the *Threshold-activated* culture refresh mode is selected. Remember that these settings are active only when the *Enable refresh* button is activated (*see* **section 3.2.1**), otherwise no culture refresh will occur during the experiment.

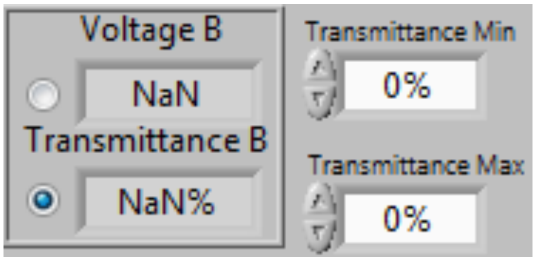

3.3.2 Pinch time

When a new experiment is initiated using the *Threshold-activated* mode, the user must define the duration of pinch cycles that dilute the culture when the specified transmittance threshold is reached. This can be done by adjusting the pinch time setting in the *Pinch time* box located at the

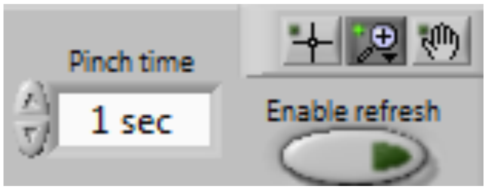

left of the *Enable refresh* button. By default, the pinch time is set to 1 sec. The minimal value allowed by the program is fixed to 0.1 sec. Always verify the maximal pinch time tolerated by your culture system before increasing it in the context of a real continuous culture experiment because extended pinches could cause a culture overflow. Instead of executing an extended single pinch cycle, it is strongly suggested to perform multiple short pinch events to avoid this problem (*see* **section 3.3.3**). The pinch time setting is also used in the context of the *Time interval* mode, but only when the refresh inactivation mode is set to *Time-inactivated* (*see* **sections 2.4.6 and 3.4.3**).

3.3.3 Number of pinch cycles

When the *Threshold-activated* mode or the *Time interval* mode (with the refresh inactivation mode set to *Time-inactivated, see* **section 3.4.3**) is selected, the user needs to specify to the program the number of pinch cycles that have to be executed within each culture refresh cycle. While in some cases only one pinch event may be sufficient to properly dilute the growing culture, some experiments may require higher culture dilutions and thus more pinch cycles. The number of pinch cycles can be set in the *Pinch cycles* field located just below the *Pinch time* box. The default value is fixed to 1 cycle, meaning that one pinch event of the desired pinch time length happens in each culture refresh cycle.

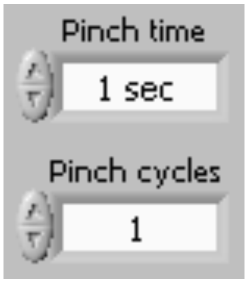

3.3.4 Maximal pinch time and pinch cycles

While the number of pinches and their duration must be defined in the *Threshold-activated* culture refresh mode, these parameters do not exist in the *Real-time feedback loop* mode since the system automatically stops the culture dilution when a certain transmittance threshold is reached. However, it is possible to set a maximal pinch time, as well as a maximal number of pinch events allowed to reach that threshold. If the system is not able to reach the transmittance threshold within the maximal pinch time, it will proceed to another pinch cycle until the maximal number of pinch cycles is reached. These settings are only available in the *Real-time feedback loop* mode, as well as in the *Time interval* mode set to the *threshold-inactivated* refresh inactivation mode (*see* **section 3.4.3**). The maximal pinch time and pinch cycles can be set in the *Max pinch time* and in the *Max pinch cycles* fields, respectively. The default value of the maximal pinch time is fixed to 5

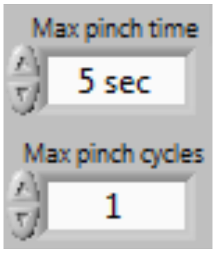

sec, while the minimal value is restricted to 0.1 sec. The default value of the maximal number of pinch cycles is set to 1, which is also the minimal value that can be selected. It is always a good idea to verify the maximal pinch time tolerated by your system and set the maximal pinch time accordingly to avoid any culture overflow. If the system is not able to reach the transmittance threshold within the specified maximal pinch time and pinch cycles, this may be due to a culture system malfunction or culture contamination. If this happen, the culture refresh cycle and the data acquisition will be stopped for the concerned channel, and an error message will appear on the screen suggesting culture system inspection.

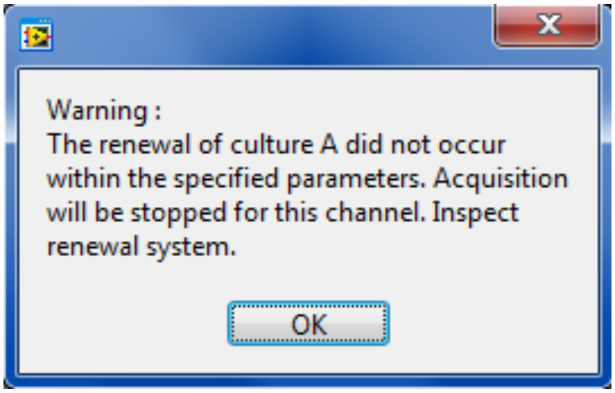

3.4 Refresh settings specific to the *Time interval* culture refresh mode

This section of the manual explains the purpose of settings and action buttons displayed in the experiment settings area that are specific to the *Time interval* culture refresh mode (*see* **section 2.4.6**).

3.4.1 Interval time

The *Time interval* culture refresh mode is used to start culture refreshing every time that a certain lapse of time has elapsed. When this mode is selected, this lapse of time called the interval time must be defined, no matter what *Time interval* specific modes are chosen (*see* **section 2.4.6**). By

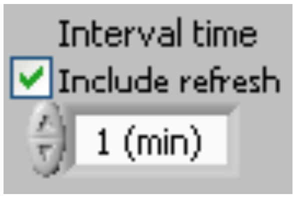

default, the interval time setting is set to 60 min, but can be modified by entering the desired value in the *Interval time* field or using the arrows. The Interval time minimal value is restricted to 1 min. This parameter is functional only if the interval timer is initialized and if the *Enable refresh* button is activated (*see* **section 3.2.1**). The interval timer can be initialized through different types of signal (*see* **section 3.4.2**). The interval time setting can either include the culture refresh time (i.e. the time needed to execute a refresh cycle), or exclude that period. By default, the interval time includes the refresh time, but this period of time can be excluded by removing the checkmark from the *Include refresh* setting box. For instance, if the interval time is set to 60 min, and the include refresh setting is activated, all refresh cycles will be exactly separated by 60 min. On the contrary, if the include refresh setting is disabled, each refresh cycle will be separated by 60 min plus the time needed to execute the previous culture refresh cycle. If the include refresh setting is abled, it is important to not set an interval time lower or equal to the time needed to refresh your culture, otherwise the system will constantly be refreshing the culture vessel.

3.4.2 Interval timer initialization

In the *Time interval* culture refresh mode, the VCCD initiates culture refreshing periodically according to the time interval parameter (*see* **section 3.4.1**). However, the interval timer, i.e. the program that counts the amount of time elapsed since the last refresh, needs to be initialized to

trigger the periodic activation of the pinch valve. The interval timer is initialized when a certain signal is provided to the VCCD software, and when the *Enable refresh* button is also activated (*see* **section 3.2.1**). Depending on which interval timer start parameter was selected during the data file creation (*see* **section 2.4.6**), this signal can be a transmittance value (*Transmittance reaches a desired value*), a specific time of the day (*At a user specified time*), or simply a manual activation (*Manually)*. If the manual activation was selected, a *Start interval timer* button appears over the *Interval time* field in the experiment setting area. Simply click on this button to initialize the interval timer, which also automatically activates the *Enable refresh* button (*see* **section 3.2.1**) that is manually inaccessible in that mode. Click again on the *Start interval timer* button to inactivate the interval timer and by the same occasion the culture refreshing capacity (*Enable refresh* button).

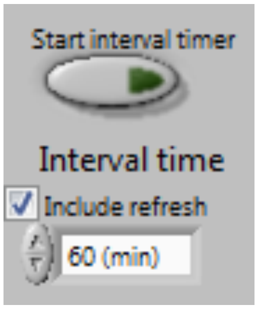

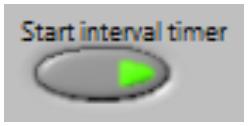

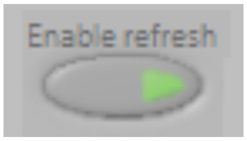

However, if the user selected the *Transmittance reaches a desired value* option to initialize the interval timer, a transmittance selector will instead be displayed (*Interval timer starts at x% of transmittance*). When the monitored transmittance reaches the chosen transmittance value, the interval timer is initialized and the pinch valve is activated periodically according to the interval time setting. To reset the interval timer initialization, simply inactivate and then reactivate the

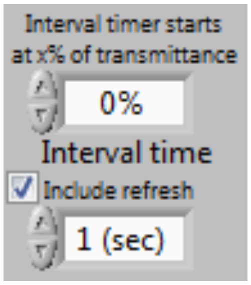

Enable refresh button. Finally, if the user chose to activate the interval timer at a specific time of the day, a small window is displayed over the interval time setting in which the desired date and time can be set (*Interval timer starts at:*). This date can also be configured by clicking on the small calendar icon that opens a calendar containing more options. Remember that the interval timer can

only be initialized if the *Enable refresh* button is also activated (*see* **section 3.2.1**). To reset the interval timer initialization, select another date in the *Interval timer starts at:* field and reset the *Enable refresh* button.

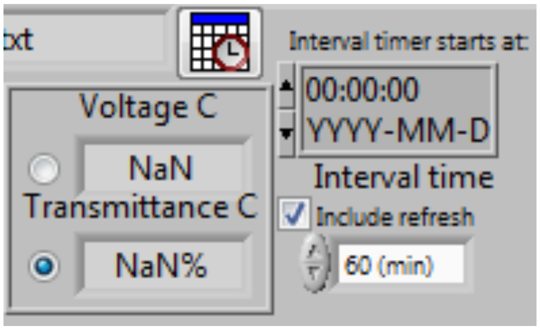

3.4.3 *Time interval* refresh inactivation modes

In the *Time interval* culture refresh mode, refreshes occur according to a user-specified period of time (*see* **section 3.4.1**). However, the signal that triggers the release of the pinch valve and stops culture dilution needs to be defined. This signal can either be a transmittance threshold (*Threshold-inactivated*) or a maximum amount of time elapsed (*Time-inactivated*) during a refresh cycle (*see* **section 2.4.6**). When a time period is selected to stop the refresh cycles, the same settings used in the *Threshold-activated* culture refresh mode are loaded in the experiment settings area, i.e. the pinch time and pinch cycles settings (*see* **sections 3.3.2** and **3.3.3**). On the other hand, if a transmittance threshold is selected, the max pinch time and max pinch cycles settings (*see* **section 2.7.4**) used in the *Real-time feedback loop* mode are displayed in the experiment settings

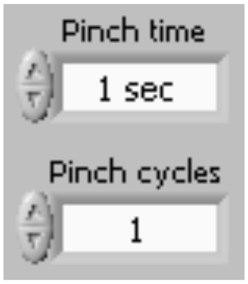

area. In addition to the max pinch time and max pinch cycles settings, one of the two available transmittance thresholds (*see* **section 3.3.1**) is also be loaded in order to signal the release of the pinch valve and stop culture dilution. This threshold can either be the minimum or the maximum transmittance threshold depending on the previously selected *Expected signal curve* (*see* **section 2.4.5**). If the *Transmittance increases with cell density* parameter is chosen, the minimum transmittance threshold is loaded in the experiment settings area and is used to signal the release of

the pinch valve. On the contrary, if the *Transmittance decreases with cell density* parameter is selected, the maximum transmittance threshold is loaded and is used to stop the culture dilution event.

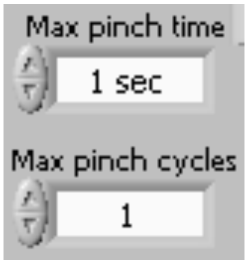

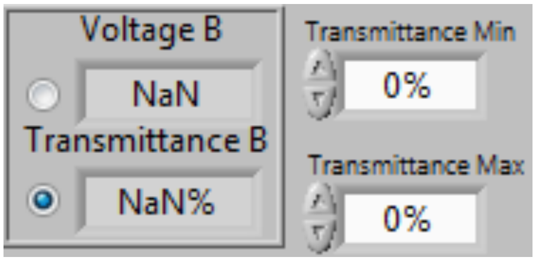

Appendix 1 - Frame machining details (S1 Appendix)

FR-A1

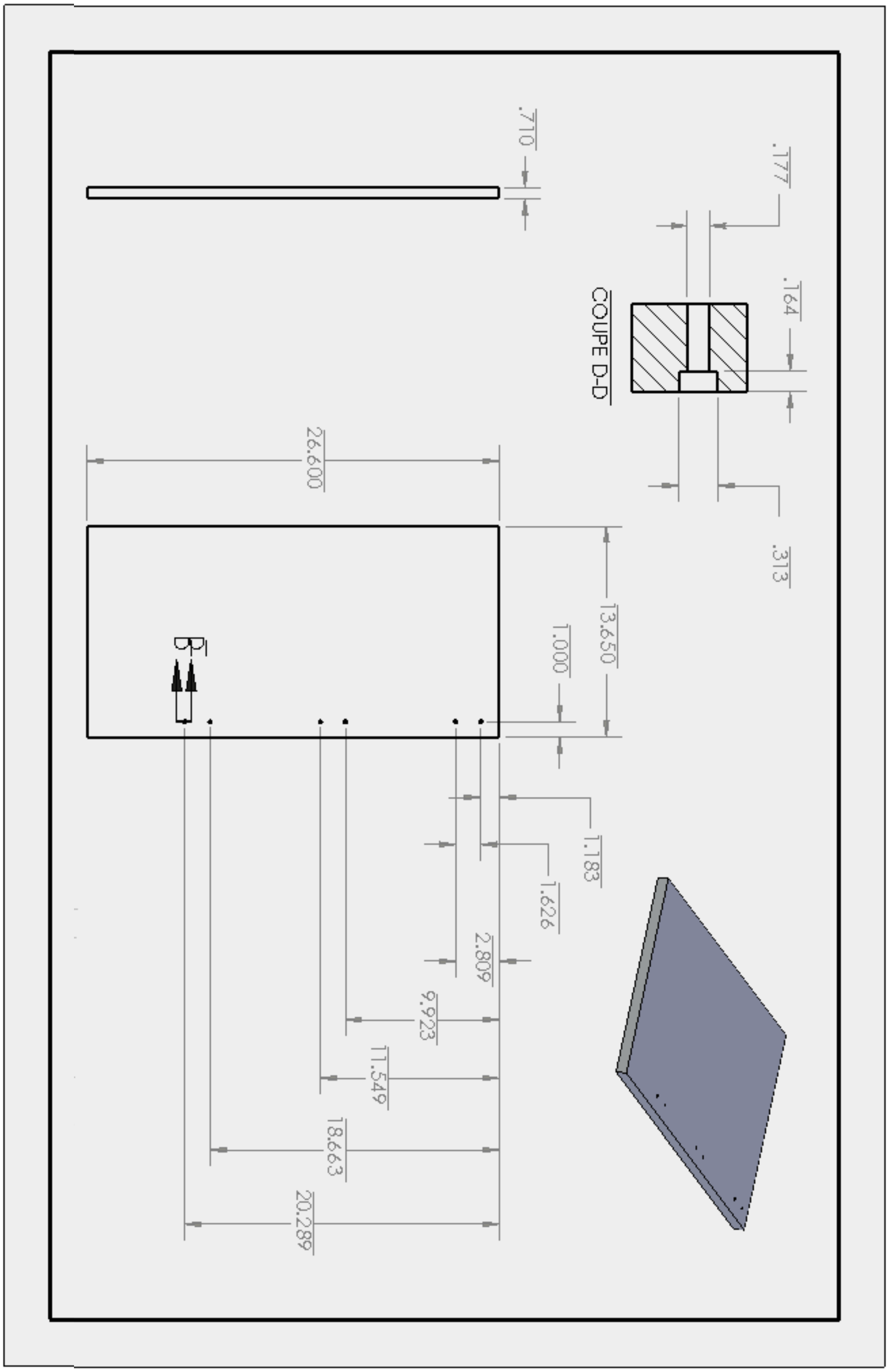

FR-B1

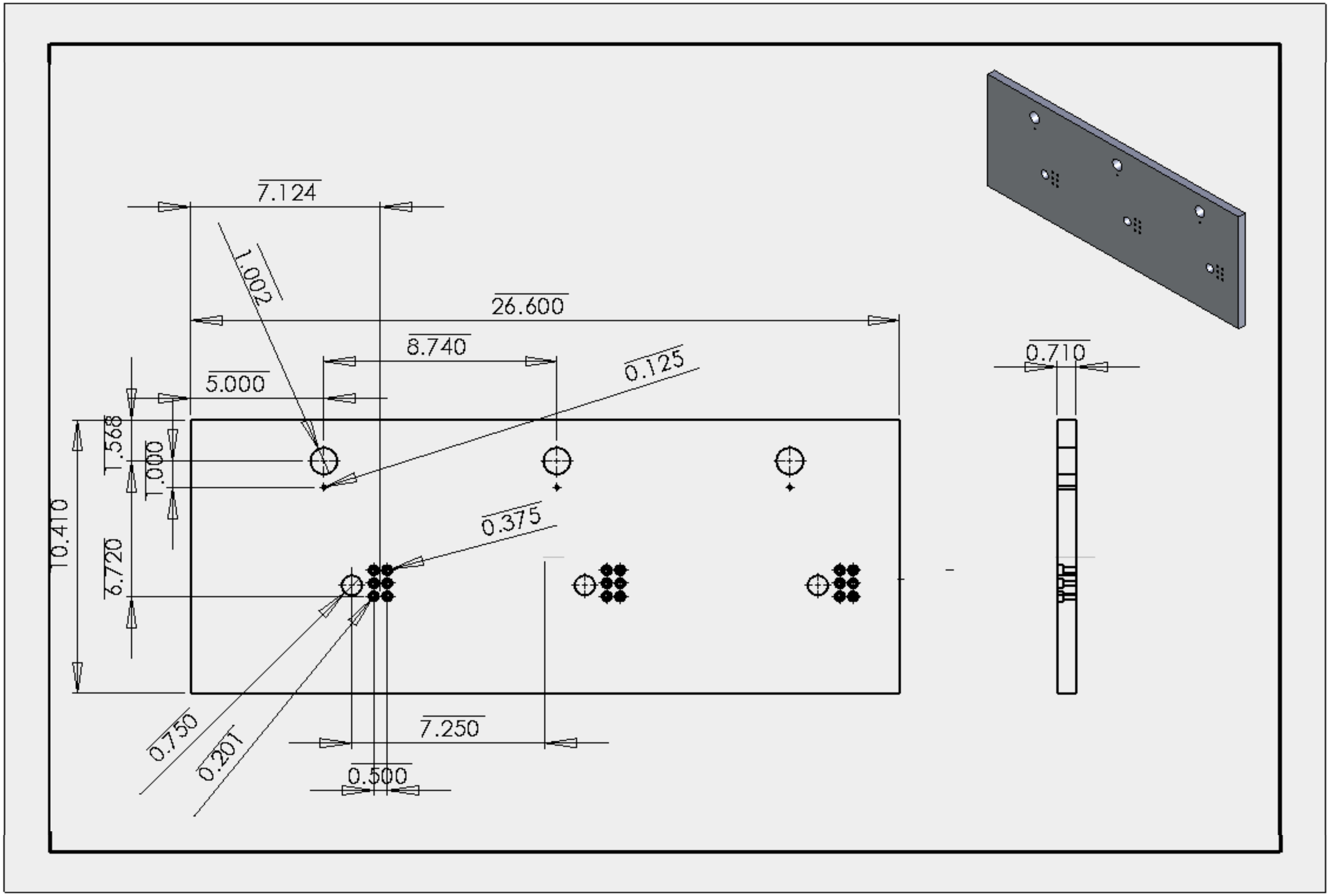

7.124
1.002
26.600
8.740
0.125
5.000
1.568
1.000
10.410
6.720
0.375
0.750
0.201
7.250
0.500
0.710

FR-C1

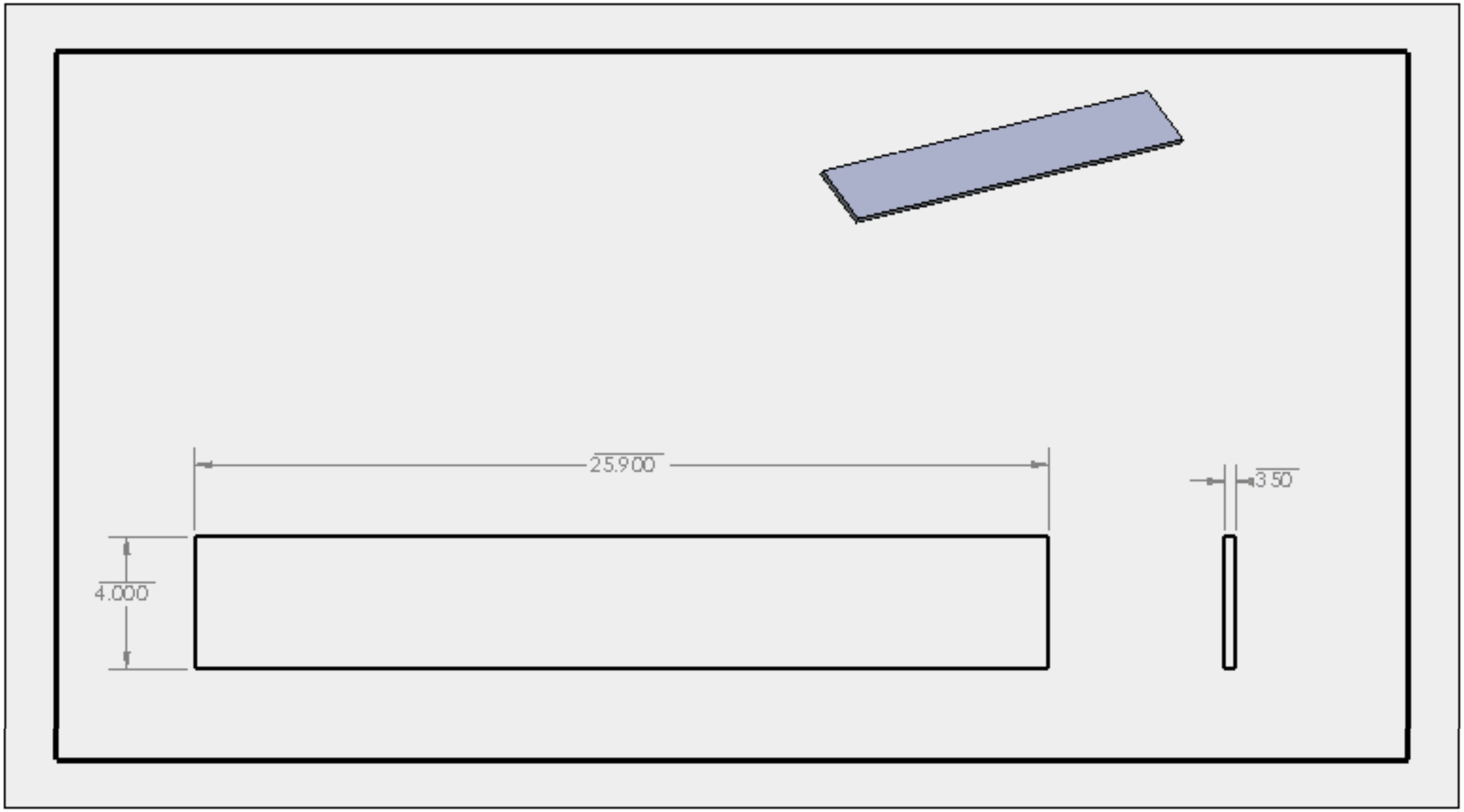

FR-D1; FR-D2

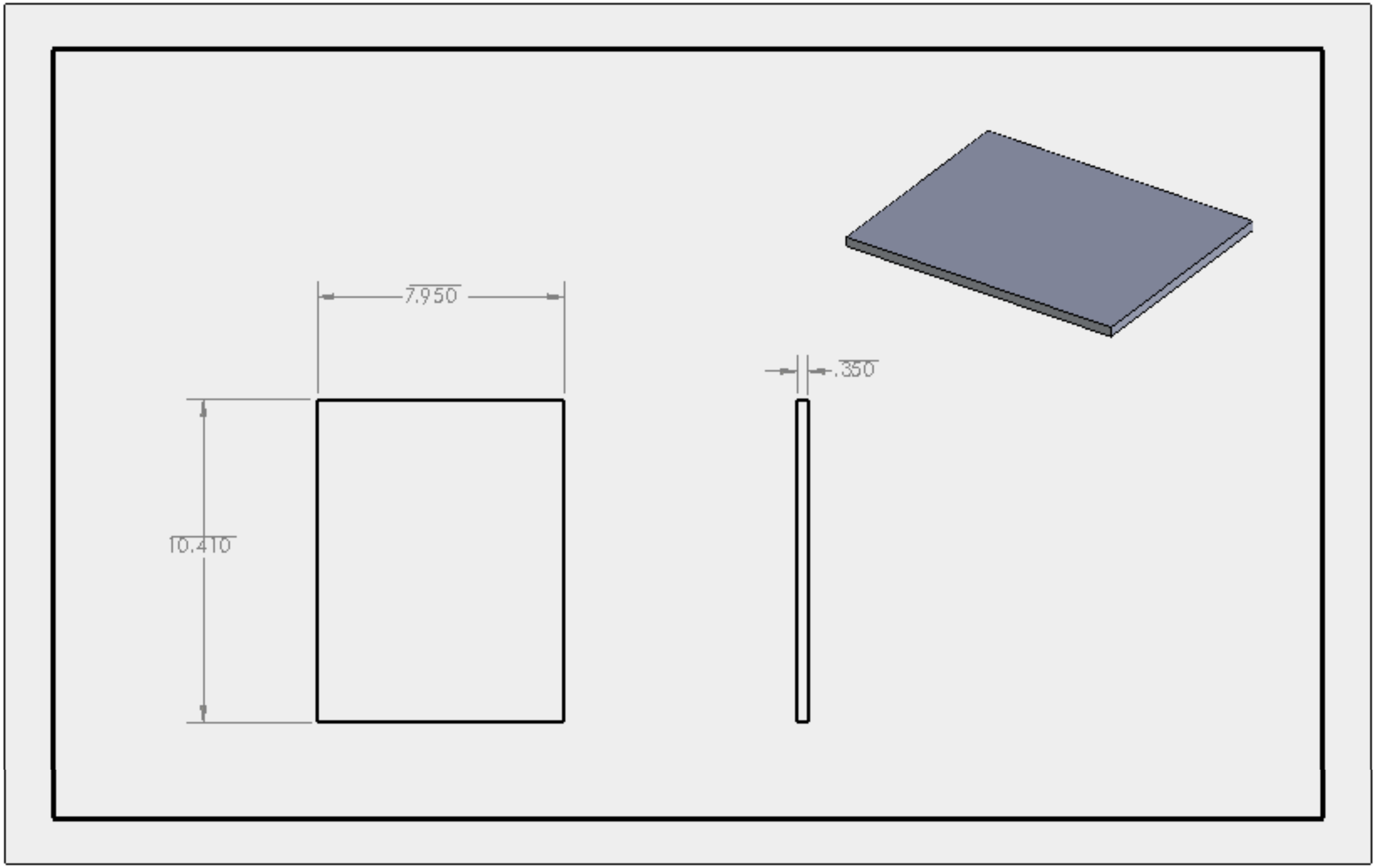

FR-E1

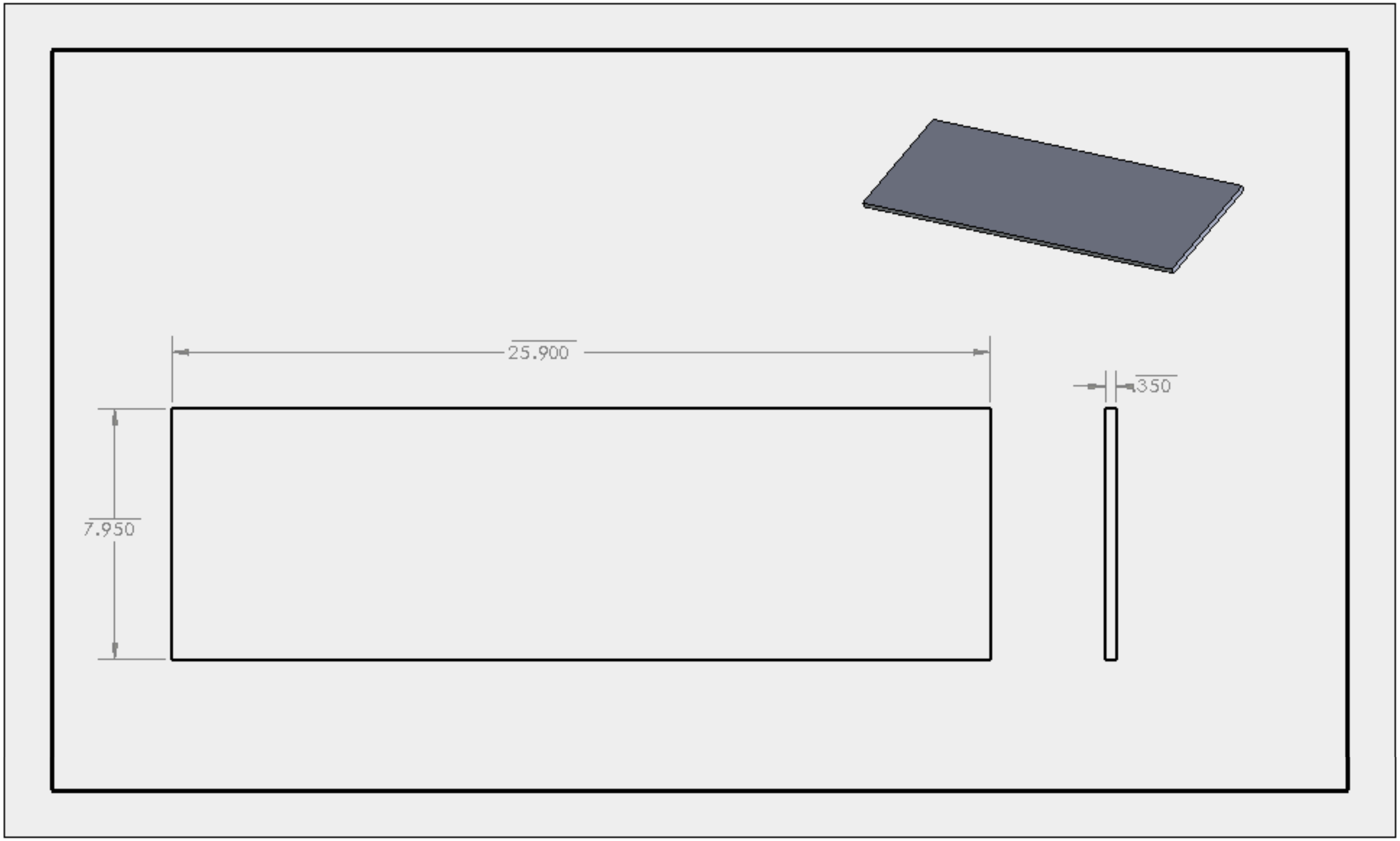

FR-F1

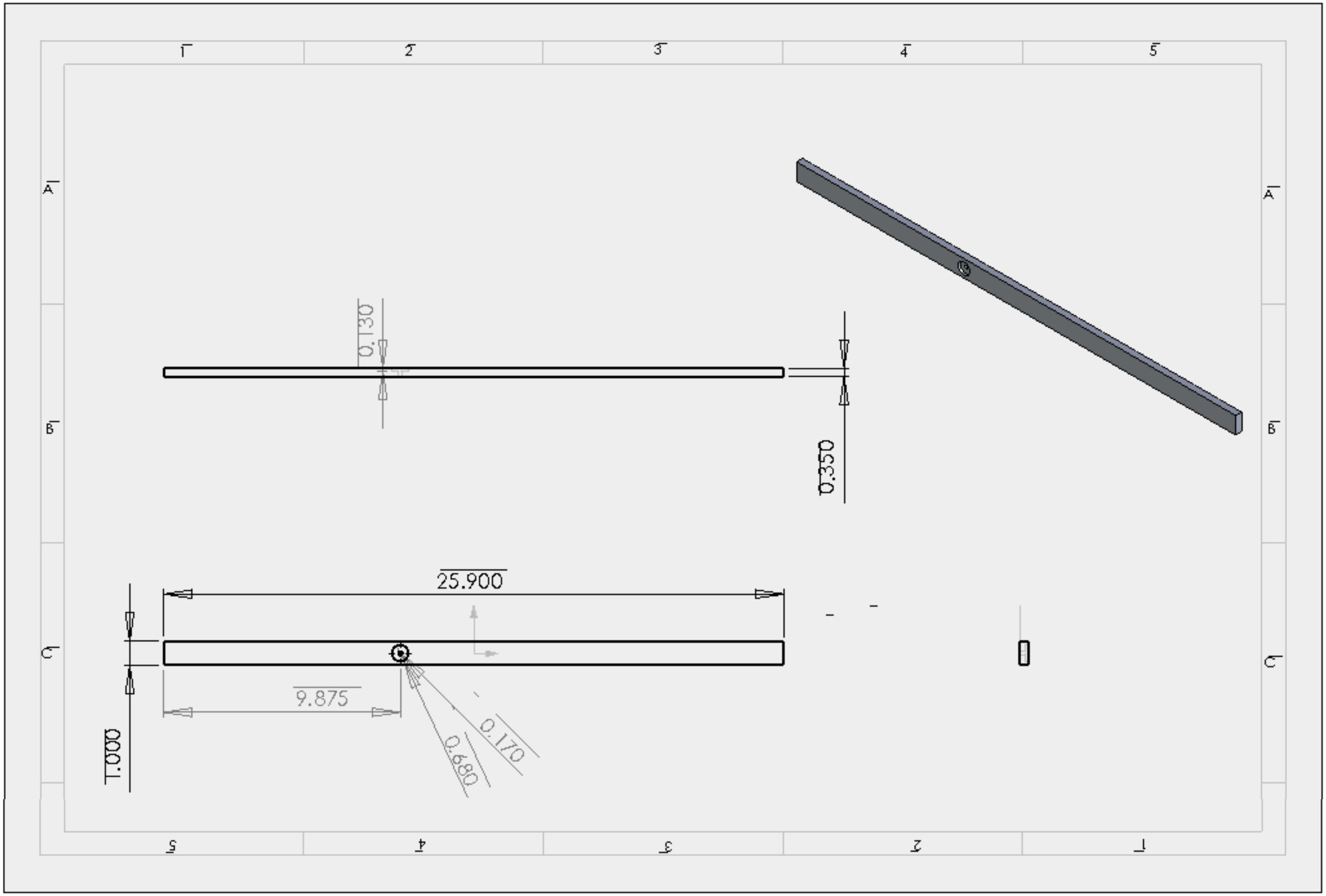

FR-G1; FR-G2; FR-G3

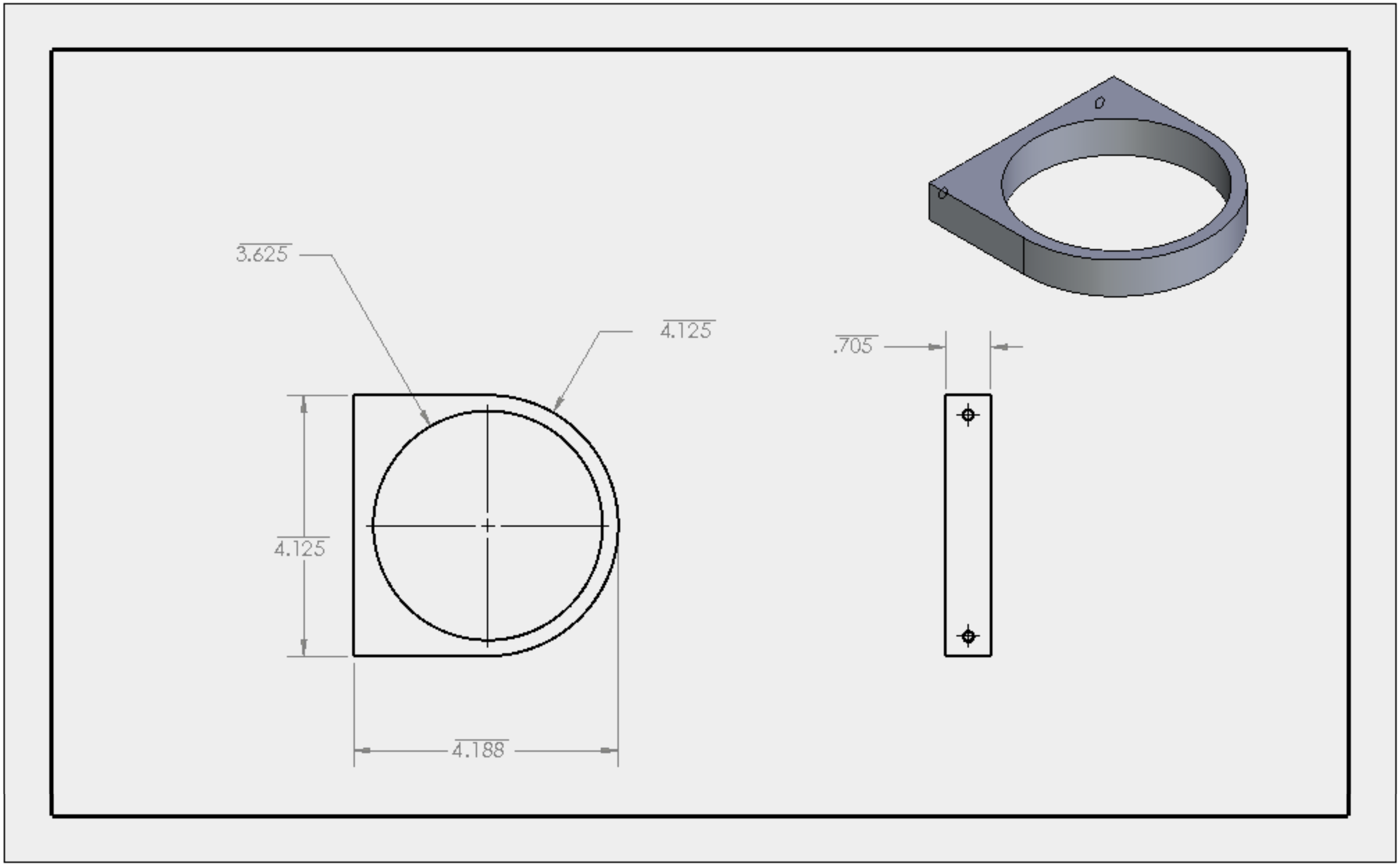

FR-H1; FR-H2; FR-H3

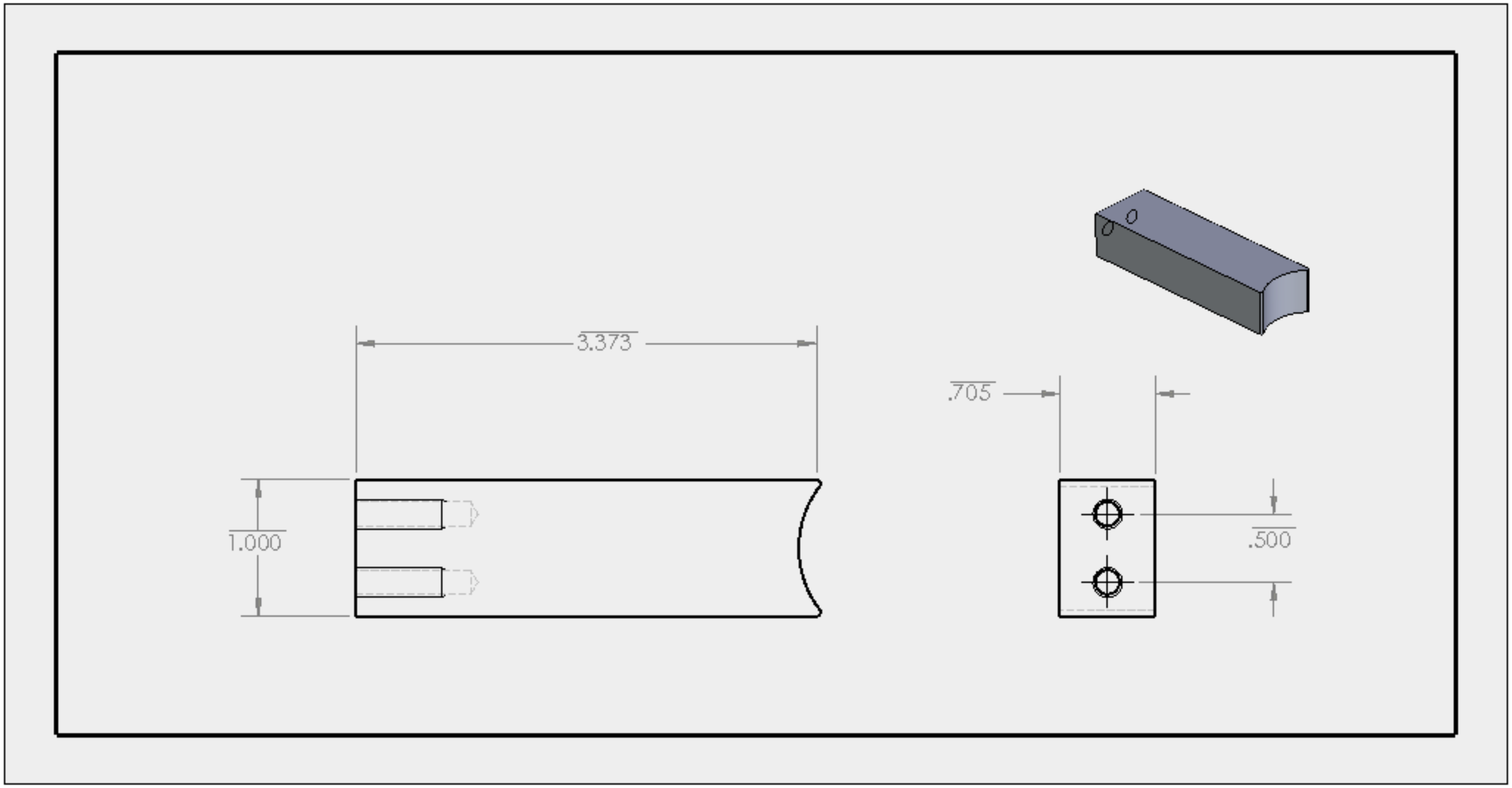

FR-I1; FR-I2; FR-I3

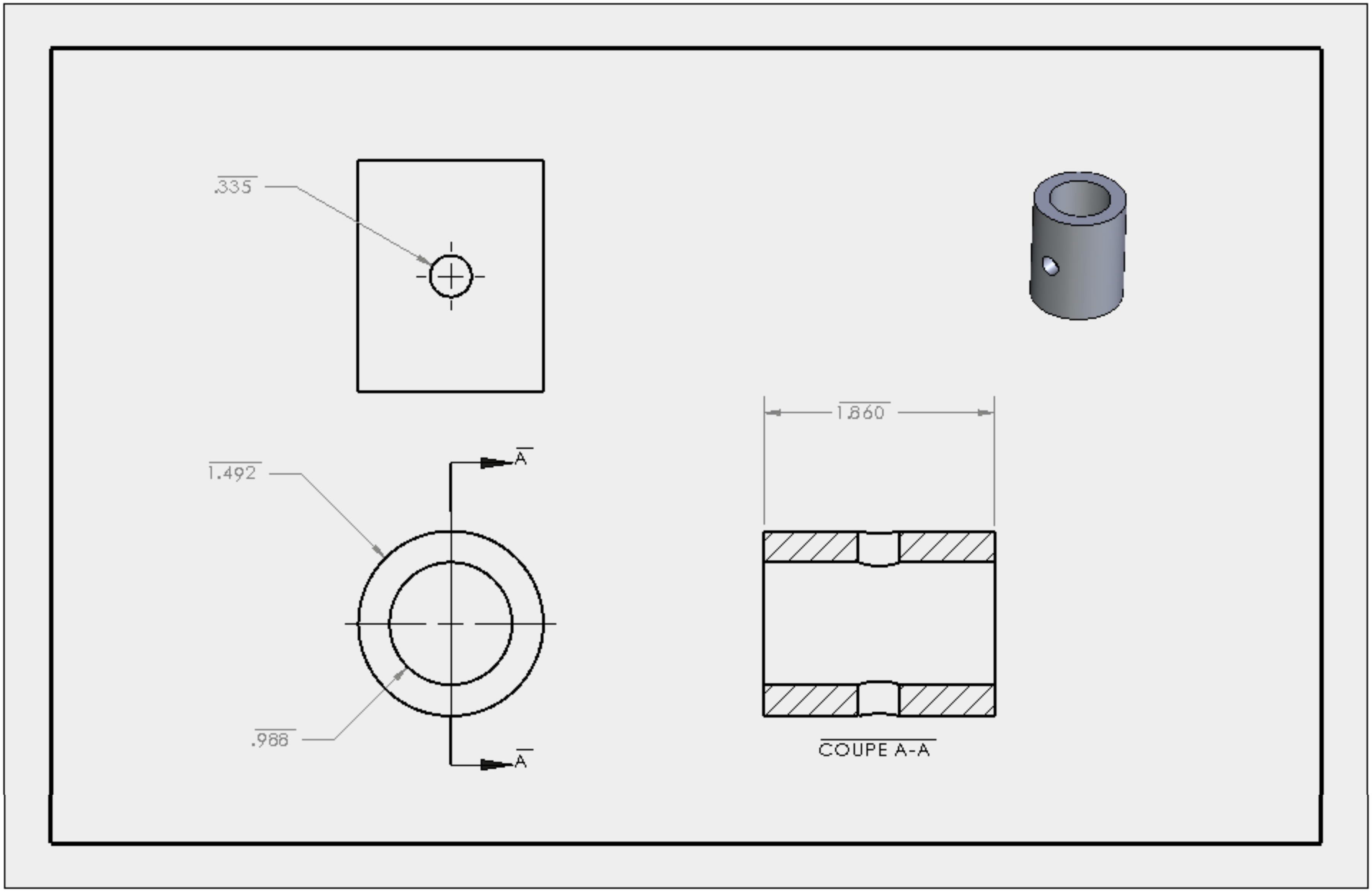

FR-J1; FR-J2; FR-J3

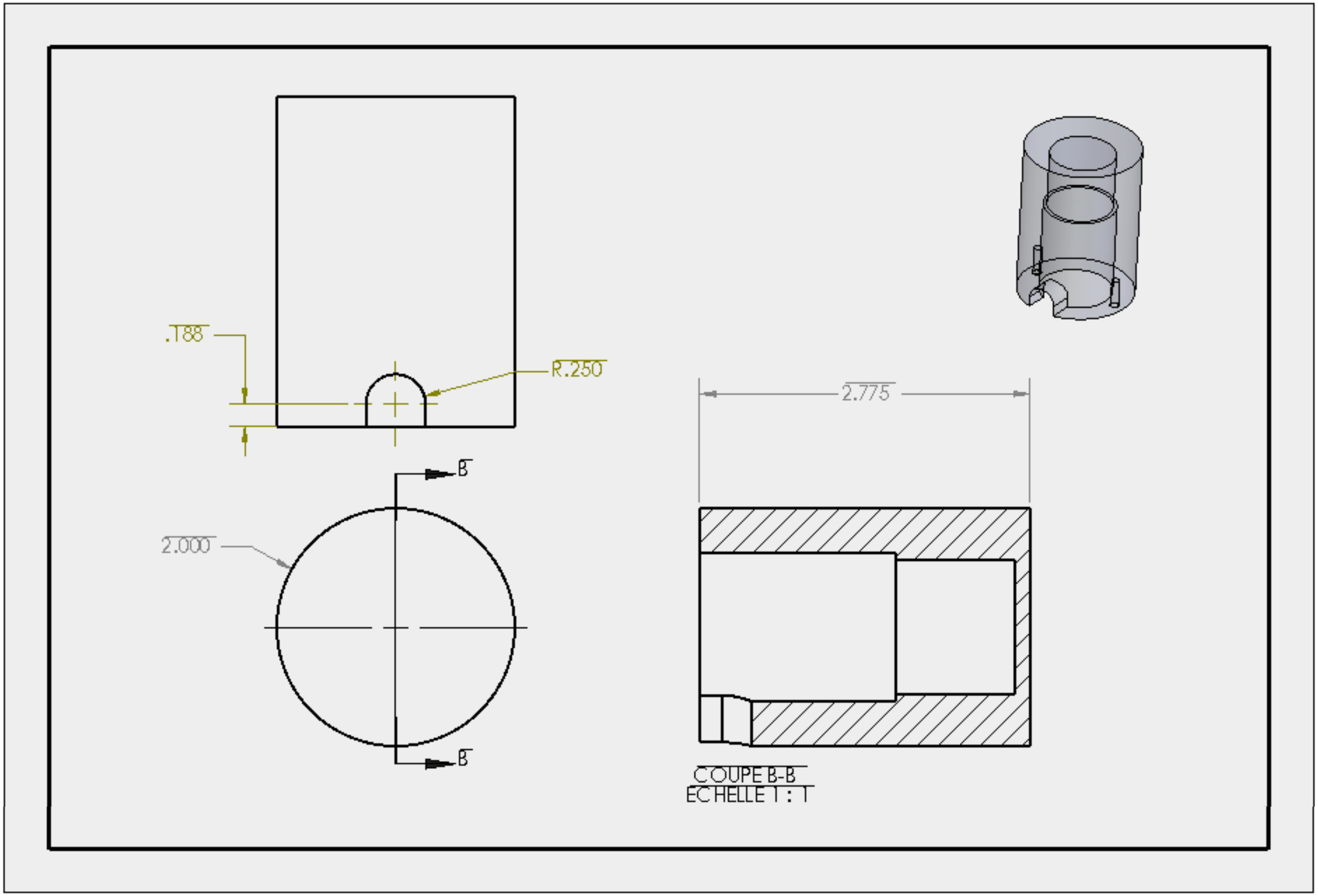

FR-L1; FR-L2; FR-L3

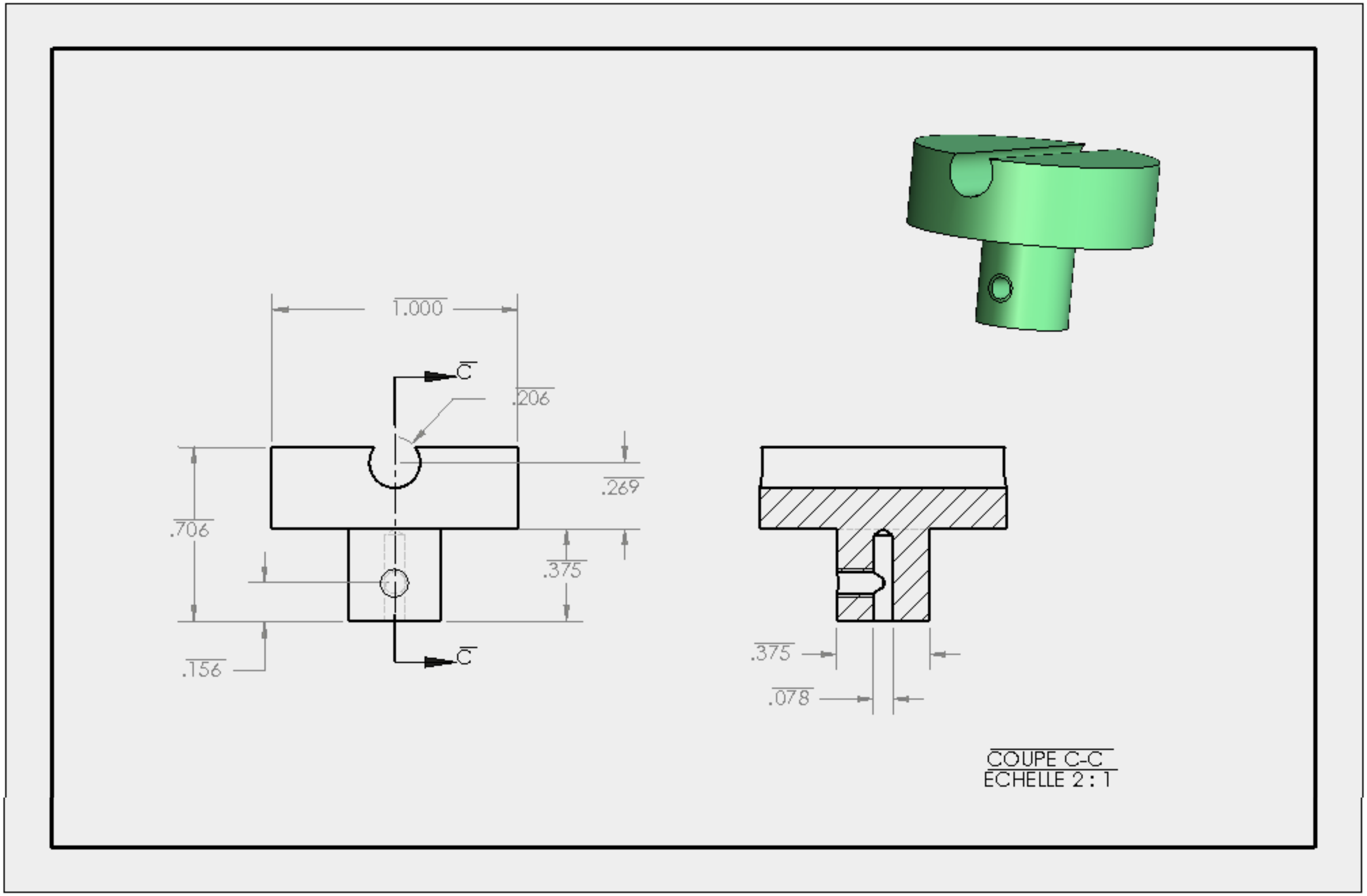

Appendix 2 – Example of a typical procedure

Here is an example a typical procedure for conducting an *E. coli* continuous culture in LB broth using the VCCD. This step-by-step quick guide provides sufficient information to allow unexperimented users to easily perform a continuous culture of *E. coli* using the *Real-time feedback loop* mode and standard parameters. For more information about available options and modes, refer to the different sections of this manual.

1. **Culture system installation and VCCD initialization**

   1.1. Sterilize the previously assembled culture system by autoclaving (*see* **section 1.3.5**).

   1.2. Exchange the empty bottles (CS-H1/H2) of the autoclaved culture system by bottles containing sterile water and LB broth, respectively (this step should be done inside a biological safety cabinet to avoid any contamination).

   1.3. Replace the empty culture vessel (CS-L1) by a vessel containing LB broth inoculated with the desired *E. coli* strain (this step should be done inside a biological safety cabinet to avoid any contamination).

   1.4. Screw tightly all bottle caps on bottles to ensure culture system sealing, with the exception of the culture vessel cap that should not be tighten too tightly in order to evacuate the excess of pressure generated during culture refreshes (*see* **section 1.2.2**).

   1.5. Dispose the culture system on the VCCD frame, insert CS-D5; D6; D8; D9 silicone tubing in a pinch valve (FR-N1/N2/N3) as depicted on **Fig. 2** and untie CS-B1 and CS-E4 tubes (these tubes were tied for the autoclave sterilization, *see* **section 1.3.5**).

   1.6. Place the VCCD system inside a temperature-controlled room or an incubator set at the desired temperature.

   1.7. Connect the CS-B1 tube to the air pump (CS-A1) and turn the pump on to provide the culture system with the air flow needed to execute culture refreshes.

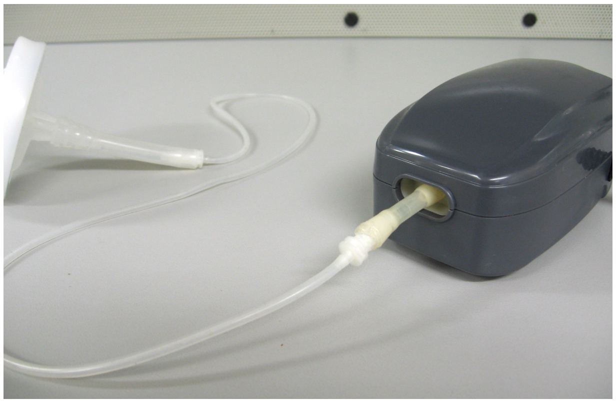

1.8. Connect the VCCD to a 120V power outlet using a standard power cord (EL-J1), connect one or more acquisition channel to the VCCD electronics box (FR-Q1), and connect the NI acquisition card of the electronics box to a USB port of the computer in which the VCCD software was previously installed (using EL-I1).

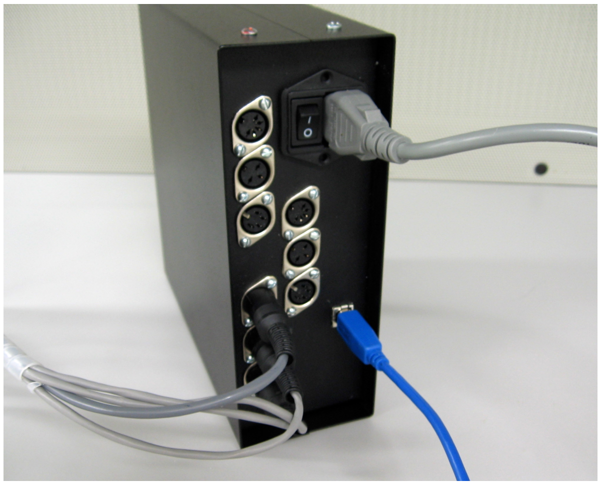

1.9. Turn on the VCCD and preheat the system for at least 20 min.

2. System calibration

2.1. Open the VCCD application folder and double click on the VCCD 1.0.exe file to start the VCCD software.

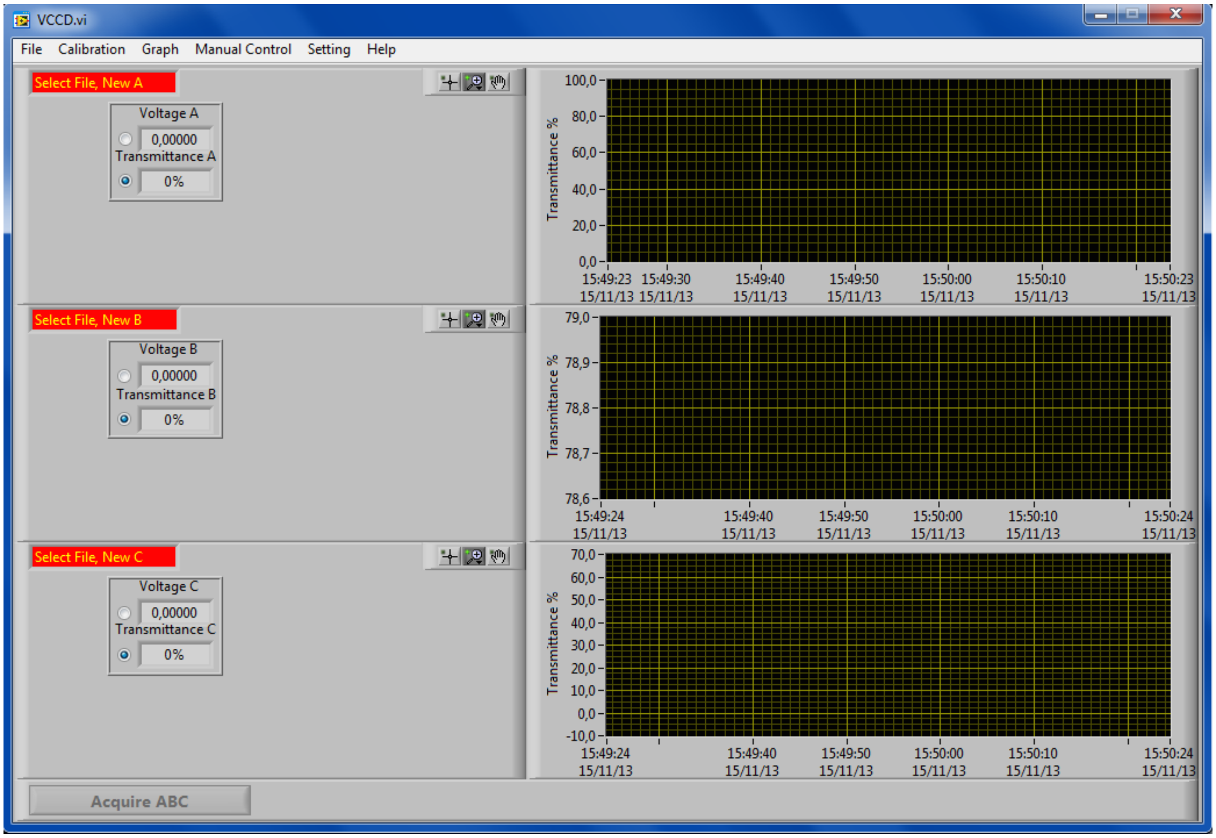

2.2. Click on the *Calibration* tab and select the channel to calibrate (*Calibrate A, B, or C*).

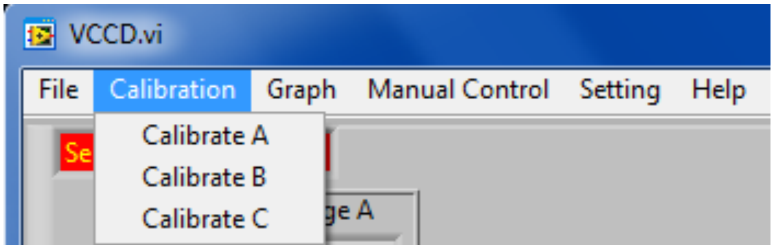

2.3. Completely cover the photo receiver with a finger to set the 0% transmittance reference value and press *OK*.

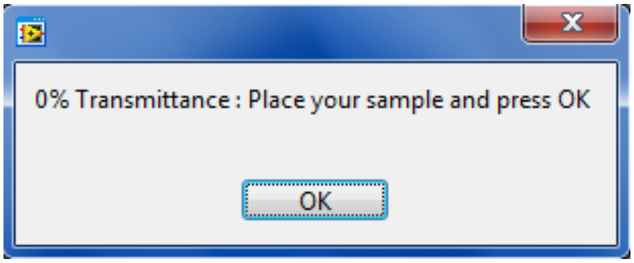

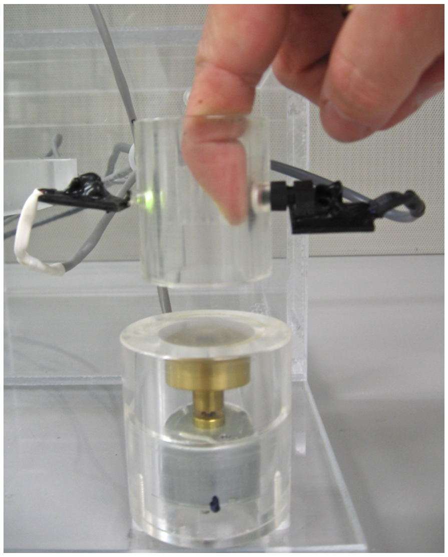

2.4. Place a culture vessel containing fresh LB broth (non-inoculated) inside the plexiglass support that holds the turbidity-measuring unit (FR-I1/I2/I3) and press *OK*. This represents the 100% transmittance reference solution.

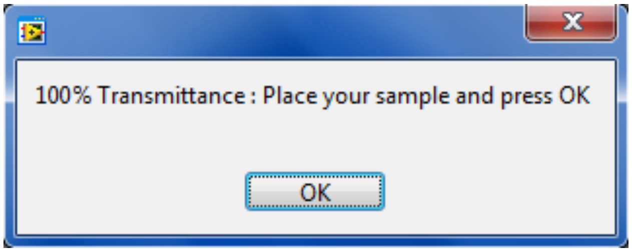

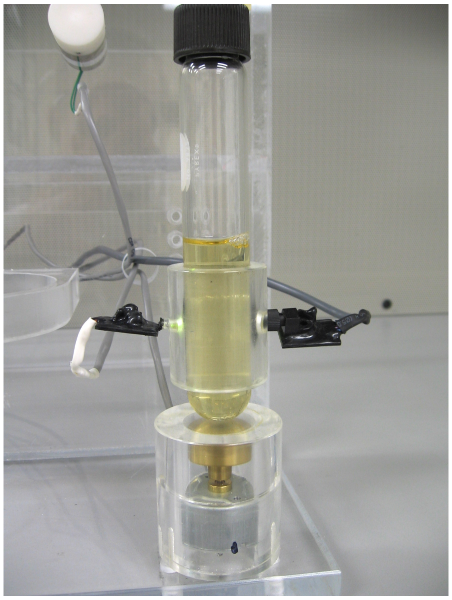

3. Choosing experiment modes

3.1. Click on the *File* tab and select a channel to start a new experiment (*New A, B, or C*).

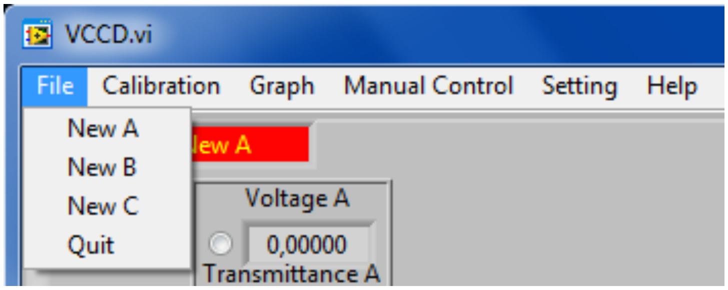

3.2. Choose the destination path as well as the name of the data file.

3.3. In the configuration window, select *Transmittance* inside the *Type of experiment* field, *Real-time feedback loop* inside the *Culture refresh mode* field, as well as *Transmittance decreases with cell density* inside the *Expected signal curve* field and press *OK*.

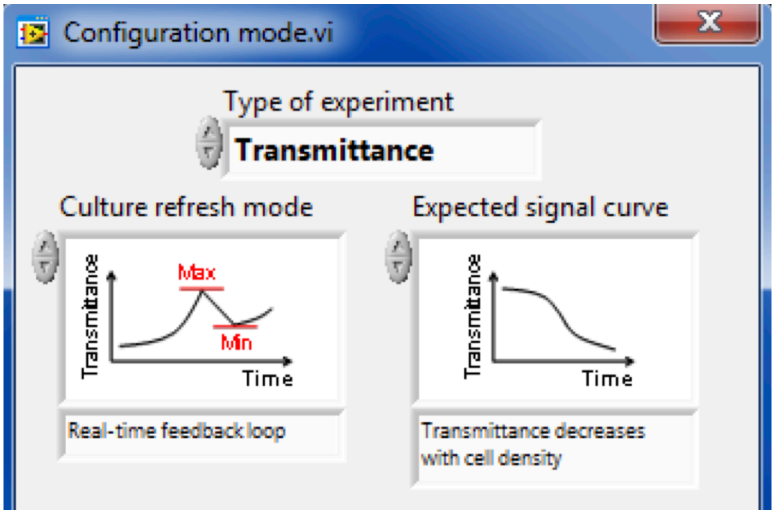

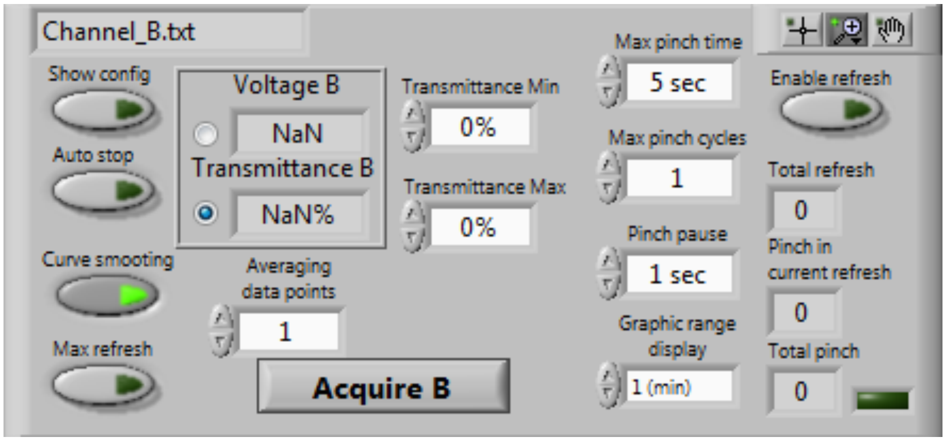

4. Setting the graphical parameters

4.1. To increase the x-axis resolution of the data graph and display a broader view of the monitored transmittance, select 60 min in the *Graphic range display* field.

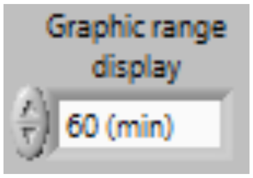

4.2. To slightly smooth data curve, change the value inside the *Averaging data points* box to 5 data points.

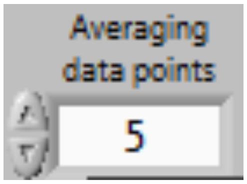

5. **Selecting refresh thresholds**
   5.1. Enter 50% inside the *Transmittance min* field to restrain the *E. coli* culture to the exponential growth phase.

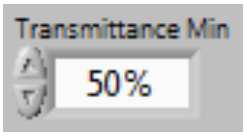

   5.2. Enter 55% inside the *Transmittance max* field to limit culture dilution to a narrow range of cell concentration.

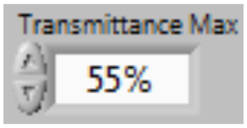

   5.3. Change the maximal number of pinches allowed within one refresh cycle to 2 pinches by entering this value inside the *Max pinch cycles* box.

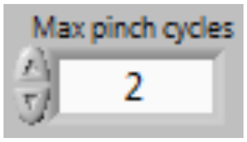

6. **Starting the experiment**
   6.1. Click on the *Acquire button* to launch data acquisition and monitor culture growth on the data graph.

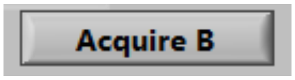

   6.2. Activate the *Enable refresh* button to allow the VCCD to perform refreshes when the transmittance reaches the minimal transmittance threshold.

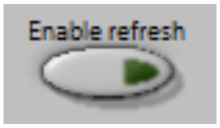

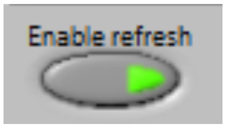

6.3. When the conditions are met to perform a refresh cycle, the green LED indicator turns on. As the experiment goes on and refreshes are executed, the refresh and pinch counters display the current refresh statistics (*see* **section 3.2.2**).

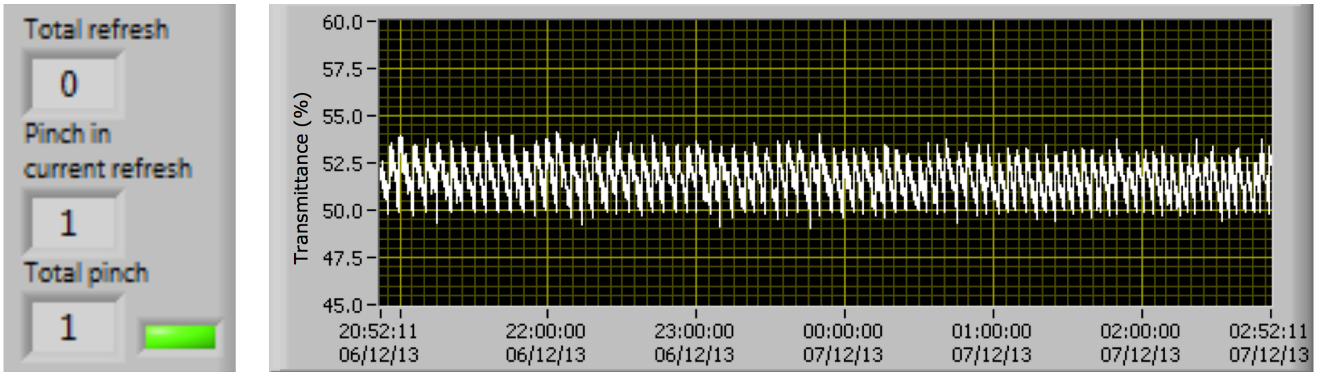

6.4. To perform a continuous culture for an extended period of time, regularly change the fresh medium and the trash bottles for new ones.
